# Metabolomics analysis reveals the differences between *Abrus cantoniensis* Hance and *Abrus mollis* Hance

**DOI:** 10.1186/s12870-023-04372-y

**Published:** 2023-08-01

**Authors:** Kexin Cao, Jianhua Chen, Rongshao Huang, Rumei Lu, Xiao Zhou, Yuanyuan Bu, Liangbo Li, Chun Yao

**Affiliations:** 1 College of Pharmacy, Guangxi University of Chinese Medicine, Nanning, 530200 Guangxi China; 2 College of Agriculture, Guangxi University, Nanning, 530004 Guangxi China

**Keywords:** *Abrus cantoniensis* Hance., *Abrus mollis*, Differentially accumulated metabolites, Chinese medicine; UPLC‒ESI‒MS/MS

## Abstract

**Background:**

*Abrus cantoniensis* Hance. (Ac) and *Abrus mollis* (Am), two edible and medicinal plants with economic value in southern China, belong to the *Abrus* genus. Due to its growth characteristics, Am often replaces Ac in folk medicine. However, the latest National Pharmacopeia of China only recommends Ac. The differences in the metabolite composition of the plants are directly related to the differences in their clinical efficacy.

**Results:**

The difference in metabolites were analyzed using an untargeted metabolomic approach based on ultrahigh-performance liquid chromatography-electrospray ionization-tandem mass spectrometry (UPLC‒ESI‒MS/MS). The roots (R), stems (S) and leaves (L) of the two varieties were examined, and 635 metabolites belonging to 8 classes were detected. A comparative study revealed clear variations in the metabolic profiles of the two plants, and the AmR group had more active ingredients (flavonoids and terpenoids) than the AcR group. The metabolites classified as flavonoids and triterpene saponins showed considerable variations among the various samples. Both Ac and Am had unique metabolites. Two metabolites (isovitexin-2''-xyloside and soyasaponin V) specifically belong to Ac, and nine metabolites (vitexin-2"-O-galactoside, ethyl salicylate, 6-acetamidohexanoic acid, rhein-8-O-glucoside, hederagenin-3-O-glucuronide-28-O-glucosyl(1,2)-glucoside, methyl dioxindole-3-acetate, veratric acid, isorhamnetin-3-O-sophoroside-7-O-rhamnoside, and isorhamnetin-3-O-sophoroside) specifically belong to Am.

**Conclusions:**

The metabolite differences between Ac and Am cause the differences in their clinical efficacy. Our findings serve as a foundation for further investigation of biosynthesis pathways and associated bioactivities and provide guidance for the clinical application of traditional Chinese medicine.

**Supplementary Information:**

The online version contains supplementary material available at 10.1186/s12870-023-04372-y.

## Introduction

Chinese herbs are effective in treating diseases and are natural sources of herbal medicine for novel bioactive compounds. Moreover, each herb contains unique active ingredients [17]. At present, many medicinal plants are facing problems, such as an increase in resource scarcity, unreasonable utilization and difficult quality control processes [7, 30], and the mechanism of action of many medicinal plants needs to be studied in depth [41].

*Abrus cantoniensis* Hance. (Ac), belonging to the genus *Abrus* of the subfamily Papilionoideae (Leguminous), is an economically valuable medicinal plant in southern China and was officially recorded in the Chinese Pharmacopoeia [32]. As an edible and medicinal plant, Ac can be used to make soup or herbal tea along with other ingredients and is commonly used as a folk medicinal supplement to prevent hepatitis and other chronic liver diseases. The dried whole plant without pods is the medicinal component, but mainly the roots and stems, which exhibit antioxidant, antiviral, immunomodulatory, hepato-protective and hypolipidemic effects, are used. Numerous studies have revealed that flavonoids, phenolic acids, alkaloids and terpenoids are the components responsible for these activities [9, 21, 37, 44].

*Abrus mollis* (Am) is a similar species within the same *Abrus* genus. Both Am and Ac contain alkaloids, flavonoids, amides and saponins [47, 48], but the content of each component is different [20], which may affect the pharmacological efficacy [21, 45]. Although Am was not recommended in the latest National Pharmacopeia of China, the species is also used in healthcare soups and Chinese patent medicine in folk medicine [45]. Moreover, Am is widely cultivated in the Guangdong and Guangxi provinces of China as an alternative species of Ac because its growth rate is relatively high [24]. Although much literature has been reported on the chemical composition of Ac and Am extracts, a systematic comparative study on the phytochemical constituents present in Ac and Am is lacking. Thus, a comprehensive comparative study of the metabolic components of the two species may provide a better understanding of species selection and resource utilization.

A rapid and extremely sensitive approach for identifying as many plant metabolites as possible is liquid chromatography-tandem mass spectrometry (LC‒MS/MS)-based extensively focused metabolomics analysis, which considers all the data in the database [38]. Thus, this method was used in this study to identify and quantify metabolites in three tissues of cultivated Ac and Am. The aim of our work was to elucidate the metabolic differences among cultivated Ac and Am and provide useful data for evaluating substances with medicinal value as a reference for future rational development and utilization.

## Results

### Morphological differences

**Fig. 1:**
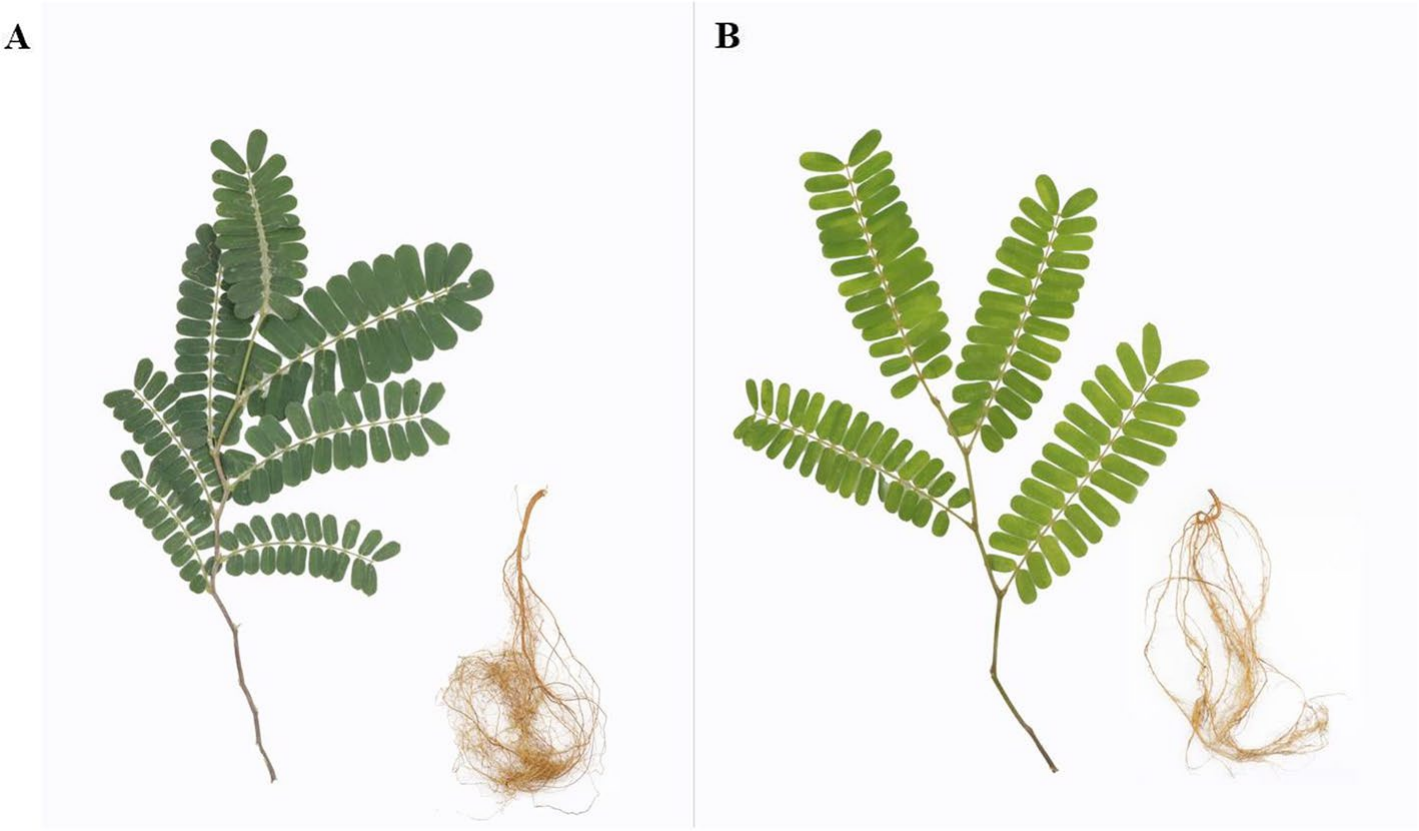
Phenotype features of two-month-old Abrus cantoniensis Hance. (**A**) and Abrus mollis Hance. (**B**)

Ac and Am were raised in the same environment and during the same growing season (Fig. 1). There are obvious differences in the morphology of the two plants, particularly in the stems and leaves. The height of Ac was usually 100-150 cm, whereas Am was usually over 150 cm tall. The stem of Am was brown and 2-5 cm in diameter, but that of Am was light green and 5-8 cm in diameter. In addition, Ac leaves show less pubescence on the surface and more pubescence abaxially, while Am leaves show more pubescence on both sides.

### Metabolite detection

**Fig. 2:**
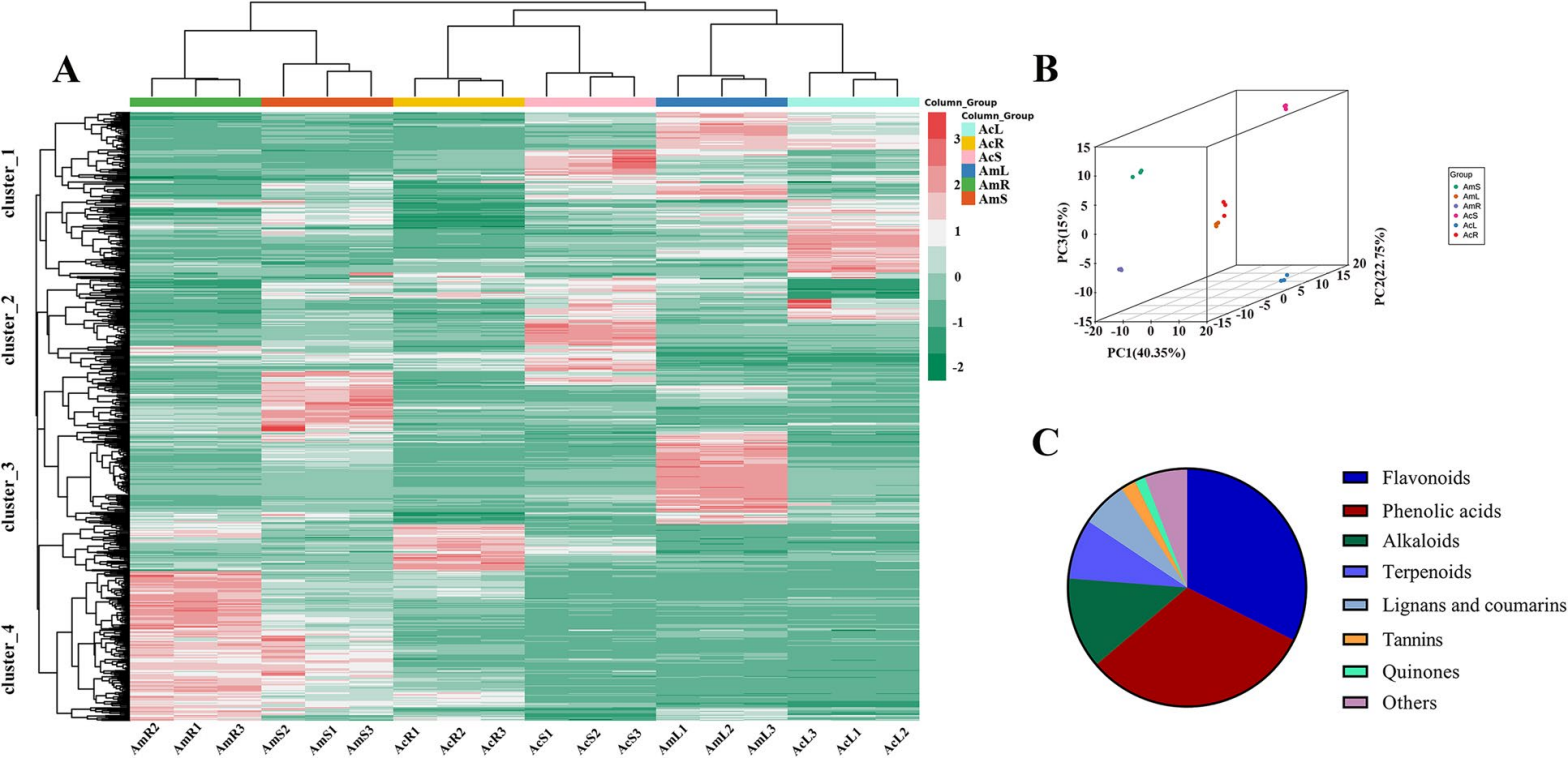
Heatmap of the HCA and PCA results showing the relative differences in the metabolite profiles between different varieties and parts (**A**), incorporated PCA plot (**B**) and pie chart of the metabolite information of all the samples (**C**)

To identify and better understand the detailed metabolite differences in the roots, stems and leaves between Ac and Am, we performed UPLC-QQQ-MS analysis for the three tissue parts of Ac and Am. The metabolites were quantitatively analyzed using software analyst under the multiple reaction monitoring modes (Supplementary Figure 1). A total of 635 metabolites were detected, including 205 flavonoids, 200 phenolic acids, 79 alkaloids, 52 terpenoids, 40 lignans and coumarins, 13 tannins, 9 quinones, and other compounds (Fig. 2C). A total of 584, 626 and 598 metabolites were detected in the roots, stems and leaves of two plants, respectively. In general, this finding suggested that different tissues displayed different metabolic profiles. Flavonoids and alkaloids were the main medicinal compounds in the two *Abrus* species. A total of 205 flavonoids were detected, and these included 61 flavonoids, 56 isoflavones, 27 flavonols, 16 dihydroflavones, 12 flavanols, 12 flavonoid carbonosides, 6 chalcones, 5 dihydroisoflavones, 2 sinensetin compounds and 8 other flavonoids (Supplementary Table 1). Approximately 79 alkaloids, including 51 alkaloids, 11 phenolamines, 7 quinolizidine alkaloids, 7 plumerane alkaloids, 1 isoquinoline alkaloid, 1 piperidine alkaloid and 1 sesquiterpene alkaloid, were detected (Supplementary Table 2).

### Identification of differences among the metabolite profiles by multivariate analysis

The metabolites with significant differences were standardized by processing, and a clustering heatmap was created for identification of the rules regulating changes in metabolites (Fig. 2A). The HCA results showed significant differences among the various groups, which were split into four groups. The metabolites in cluster 1 exhibited the highest accumulation in AmL and AcL, those in cluster 2 were found at their highest level in AmS and AcS, those in cluster 3 were most highly accumulated in AcR and AmL, and those in cluster 4 exhibited their highest accumulation in AmR and AmS. The biological replicates were also grouped together, indicating good consistency between the biological replicates and the good reliability of the data.

PCA has been used to discover the internal structure of multiple variables using several principal components. In the PCA diagram (Fig. 2B), the QC sample is a mixture of sample extracts that are projected into the same area, and some even overlap. This phenomenon indicates that the analysis was stable and reproducible. The results showed that the tissue sites were separated by the first principal component and that the varieties were clearly separated by the second principal component. A clear trend toward clustering was found between different tissues of different varieties.

Overall, HCA and PCA showed significant differences in metabolites between the various groups, and AmR and AcS exhibited the greatest differences.

### Different metabolite analysis

**Fig. 3:**
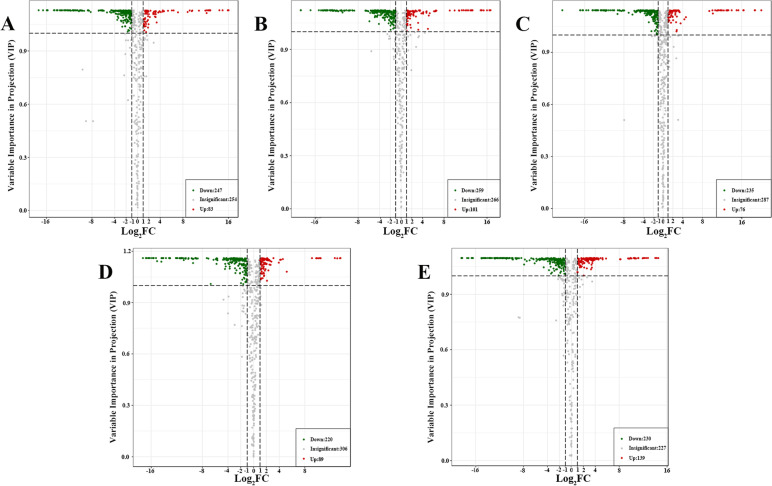
Volcano plot of differentially accumulated metabolites identified from the AmR vs. AcR (**A**), AmS vs. AcS (**B**), AmL vs. AcL (**C**), AmS vs. AmR (**D**) and AcS vs. AcR (**E**) comparisons. The colors of the scatter points indicate the final screening results: red indicates metabolites that were significantly upregulated; green indicates metabolites that were significantly downregulated; and gray indicates metabolites with no significant difference

**Fig. 4:**
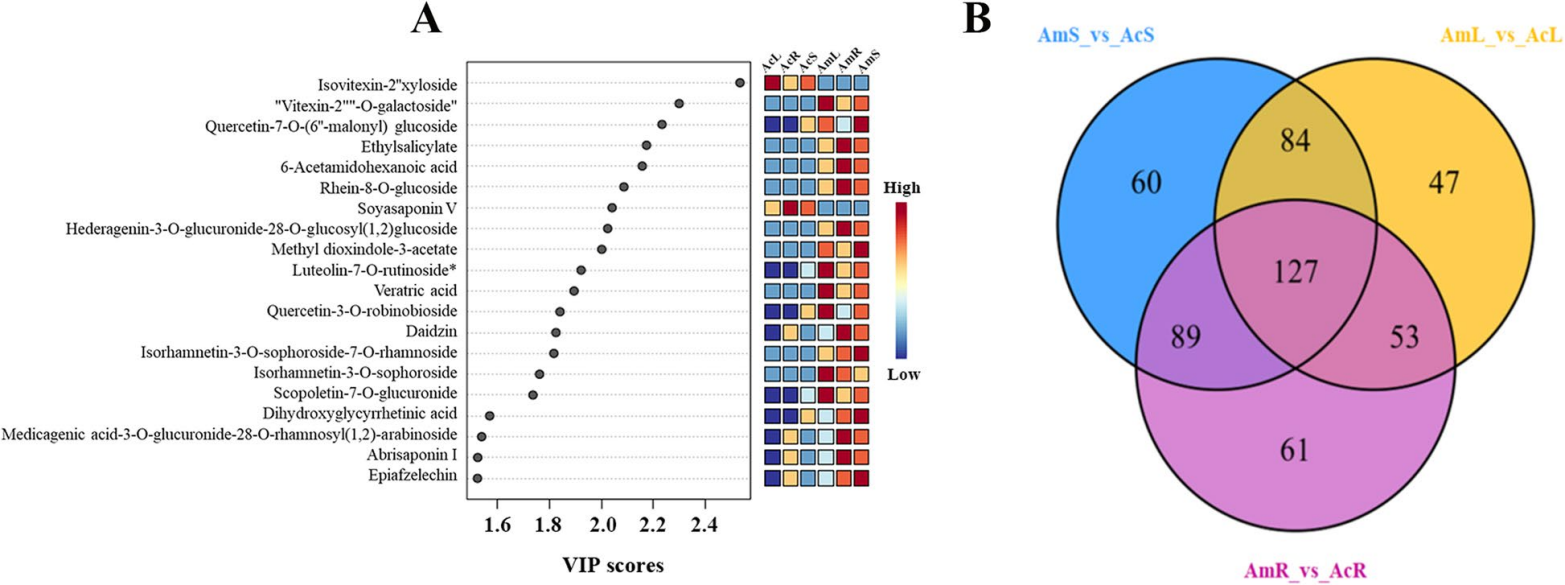
Chemical markers with VIP values larger than 1.5 (**A**) and Venn diagram between different tissues showing the overlapping and accession-specific differentially accumulated metabolites (**B**)

Orthogonal partial least squares discriminant analysis (OPLS-DA) combines orthogonal signal correction (OSC) and PLS-DA methods to filter out differential variables by removing irrelevant differences [14]. Differentially accumulated metabolites were screened based on the fold change (FC ≥ 2 or ≤ 0.5), and the variables were identified based on the variable importance in projection scores (VIP > 1) [38]. The screening results are presented as volcano plots (Fig. 3) and Venn diagrams (Fig. 4B). In the OPLS-DA models, significant segregation was found in all comparisons (Supplementary Figure 2). In this model, R^2^X and R^2^Y were used to represent the interpretation rate to the X and Y matrices, respectively, and Q^2^ represented the prediction ability. The differences between AmR and AcR (R^2^X = 0.85, R^2^Y = 1, Q^2^ = 0.995), AmS and AcS (R^2^X = 0.87, R^2^Y = 1, Q^2^ = 0.994), AmL and AcL (R^2^X = 0.833, R^2^Y = 1, Q^2^ = 0.994), AmS and AmR (R^2^X = 0.849, R^2^Y = 1, Q^2^ = 0.996), and AcS and AcR (R^2^X = 0.896, R^2^Y = 1, Q^2^ = 0.998) can clearly be observed. All the pairwise comparison results showed that the R^2^Y and Q^2^ scores were higher than 0.9, indicating that the models are stable and reliable.

A comparison of the AmR and AcR groups identified a total of 330 differentially accumulated metabolites (Fig. 3A Table 1), which included 247 downregulated metabolites and 82 upregulated metabolites, and flavonoids comprised the highest proportion of the differentially accumulated metabolites (Supplementary Table 3). The differentially accumulated metabolites included 143 flavonoids, which accounted for approximately 43.33%. In the AmR and AcR groups, 7 of the top 10 downregulated metabolites were flavonoids, namely, oroxin A, aureusidin-4-O-glucoside, kaempferol-4'-O-glucoside, biorobin, astragalin, luteolin-3'-O-glucoside and kaempferol-3-O-(6''-malonyl) glucoside. The top 10 downregulated metabolites included alkaloids, phenolic acids, terpenoids and flavonoids. Compared with AcS, 360 metabolites showed significant changes in AmS (Fig. 3B Table 2), and these included 259 downregulated metabolites and 101 upregulated metabolites. The differentially accumulated metabolites also included 148 flavonoids, which accounted for approximately 41.11%. A comparison of the AmL and AcL groups revealed a total of 311 differentially accumulated metabolites, including 235 downregulated metabolites and 76 upregulated metabolites (Fig. 3C, Table 3 and Supplementary Table 4), and flavonoids and phenolic acids comprised the highest proportion of the differentially accumulated metabolites.

**Table 1:** Classification and quantity of differentially accumulated metabolites between the AmR and AcR groups

Type	Number	percentage
Flavonoids	143	43.33%
Phenolic acids	81	24.55%
Terpenoids	32	9.70%
Alkaloids	28	8.48%
Lignans and Coumarins	17	5.15%
Others	11	3.33%
Tannins	10	3.03%
Quinones	8	2.42%

**Table 2:** Classification and quantity of differentially accumulated metabolites between the AmS and AcS groups

Type	Number	percentage
Flavonoids	148	41.11%
Phenolic acids	89	24.72%
Terpenoids	42	11.67%
Alkaloids	37	10.28%
Lignans and Coumarins	17	4.72%
Others	15	4.17%
Tannins	5	1.39%
Quinones	7	1.94%

**Table 3:** Classification and quantity of differentially accumulated metabolites between the AmL and AcL groups

Type	Number	percentage
Flavonoids	126	40.51%
Phenolic acids	82	26.37%
Terpenoids	27	8.68%
Alkaloids	31	9.97%
Lignans and Coumarins	19	6.11%
Others	9	2.89%
Tannins	12	3.86%
Quinones	5	1.61%

The roots and stems of Ac and Am are the main medicinal components, as the leaves are easily shed and hardly preserved. Therefore, the differentially accumulated metabolites between the roots and stems of Ac and Am also deserve attention. In contrast, 220 metabolites, including 76 flavonoids, were downregulated in the AmS and AmR groups (Fig. 3D and Supplementary Table 5). In Am, the stems contained higher amounts of phenolic acids and flavonoids, but the roots contained more triterpene saponin components. In contrast, (Fig. 3E and Supplementary Table 6), 230 metabolites, including 85 flavonoids, were downregulated in AcS and AcR. In Ac, the stems contained more phenolic acids and alkaloids, but the roots contained more triterpene saponin components. Therefore, we can process Am and Ac stems to use their active ingredients, such as phenolic acids. In addition, more triterpenoid saponins were observed in the underground components than in the aboveground parts.

**Fig. 5:**
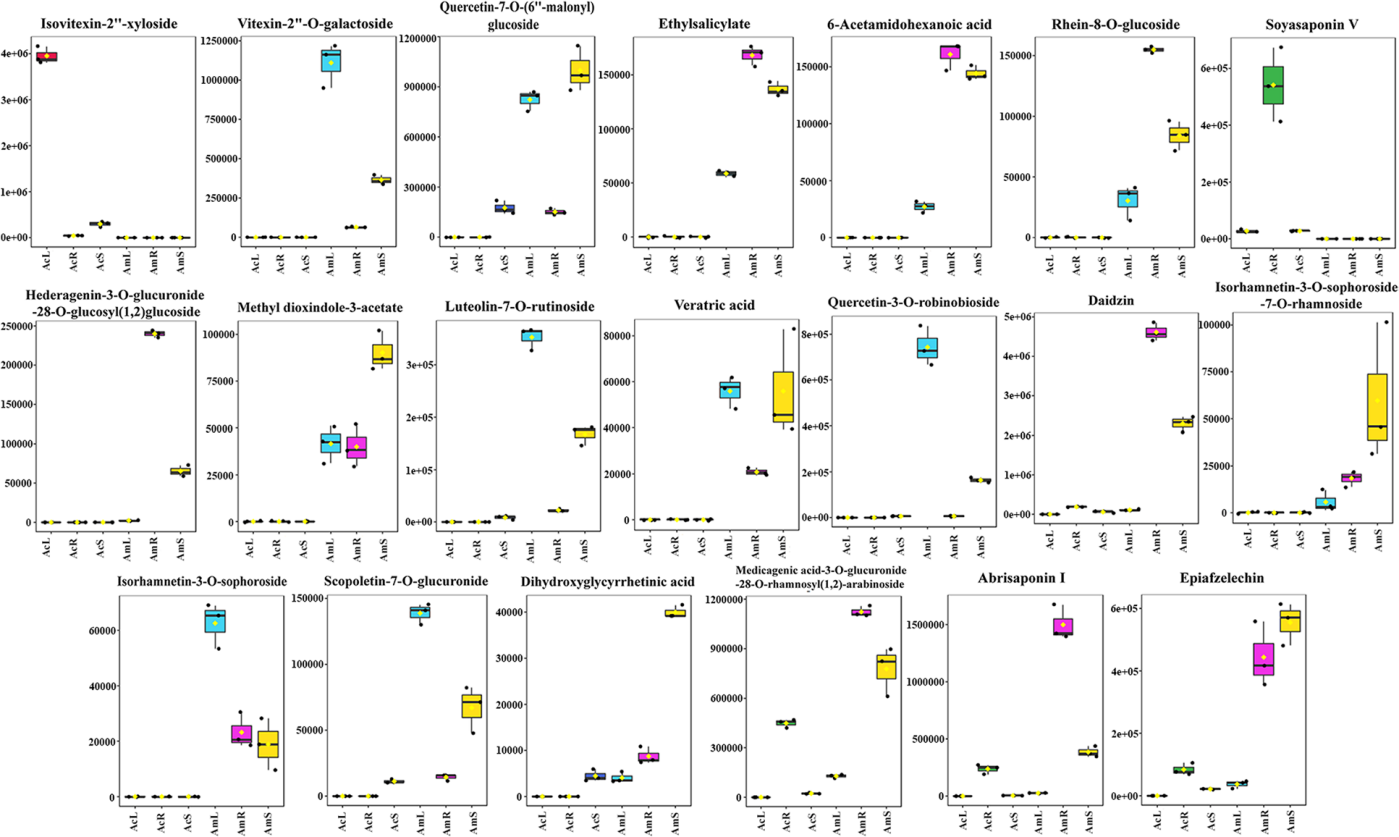
Differences in the content of nine metabolites in different tissues. The Y-scale represents the integral value of the chromatographic peak area

Based on Venn diagrams, 127 common differentially accumulated metabolites were identified. The common differentially accumulated metabolites revealed differences among the roots, stems and leaves of Am and Ac, indicating interspecific differentially accumulated metabolites. These metabolites include a number of functional substances, including flavonoids, phenolic acids, terpenoids and other types of substances. For example, acacetin, a flavonoid compound, exhibits anti-peroxidative and anti-inflammatory effects [40]. Furthermore, calycosin-7-O-glucoside (calycosin) is a highly valued herb used in traditional Chinese medicine for the treatment of cardiovascular and renal diseases [39]. The VIP of 127 features was then calculated, and 20 chemical markers were selected according to a VIP value > 1.5 (Fig. 4A and Supplementary Table 7). Through accurate mass measurements and diagnostic fragment ion production based on high-resolution tandem mass spectrometry, these chemical markers were identified into six classes, including 9 flavonoids, 2 phenolic acids, 2 alkaloids, 1 quinone, 5 terpenoids and 1 coumarin (Fig. 5). It has been reported that methyl dioxindole-3-acetate, as an alkaloid, exhibits strong DPPH radical scavenging activity [12]. Ajaghaku et al. [1] have shown that quercetin-3-O-robinobioside, a flavonoid, exhibits a potent immunopotentiating effect. Notably, two metabolites (isovitexin-2''-xyloside and soyasaponin V) specifically belong to Ac, and nine metabolites (vitexin-2"-O-galactoside, ethyl salicylate, 6-acetamidohexanoic acid, rhein-8-O-glucoside, hederagenin-3-O-glucuronide-28-O-glucosyl(1,2)-glucoside, methyl dioxindole-3-acetate, veratric acid, isorhamnetin-3-O-sophoroside-7-O-rhamnoside, and isorhamnetin-3-O-sophoroside) specifically belong to Am.

### Kyoto encyclopedia of genes and genomes annotation and enrichment analysis

**Fig. 6:**
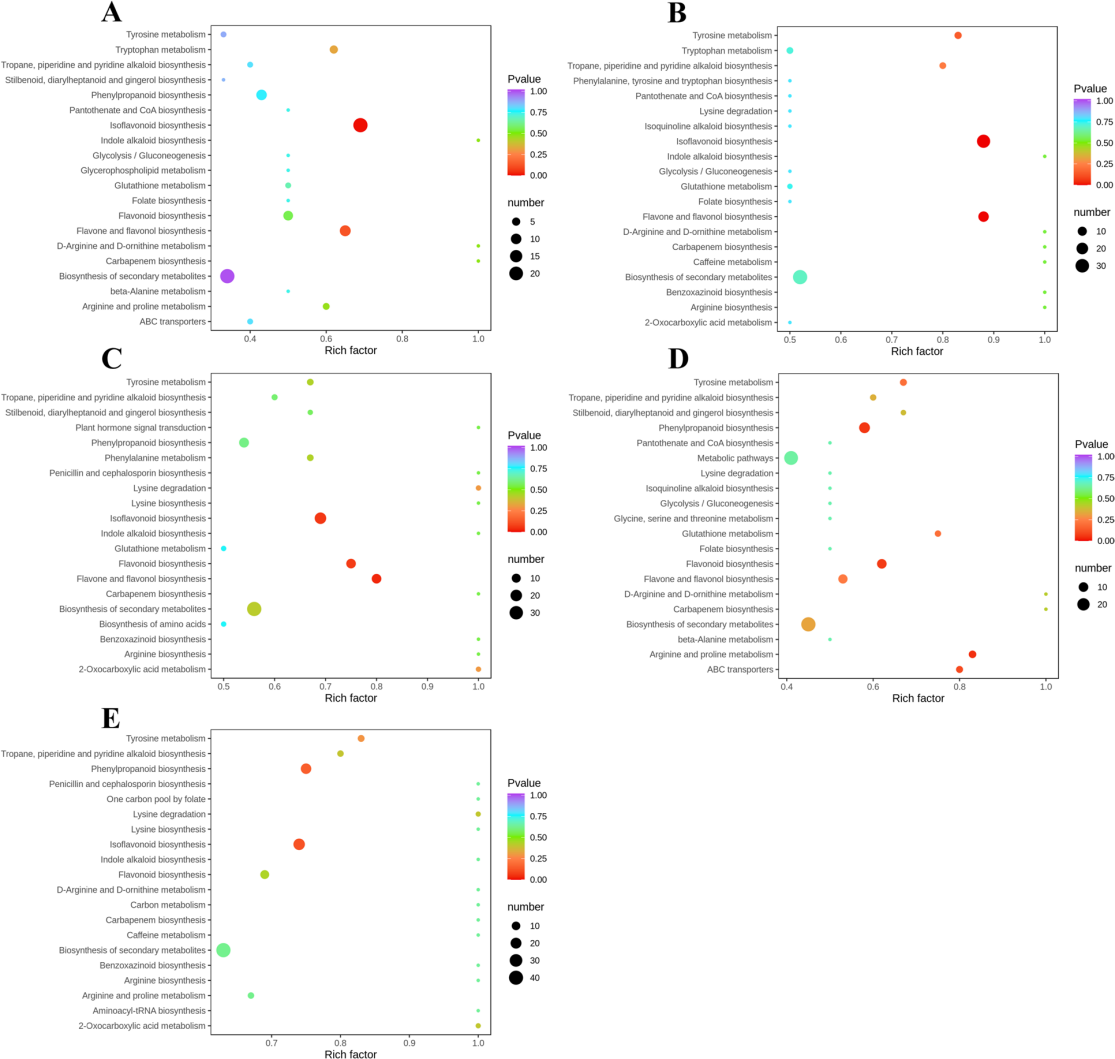
Bubble maps of the pathways enriched in the differentially accumulated metabolites. (**A**) AmR and AcR. (**B**) AmS and AcS. (**C**) AmL and AcL. (**D**) AmS and AmR. (**E**) AcS and AcR

Differentially accumulated metabolites interact in vivo to form different pathways. The differentially accumulated metabolites in each comparison group were annotated using the Kyoto Encyclopedia of Genes and Genomes (KEGG) database. The annotated results were enriched to obtain pathways with more differentially accumulated metabolites (Fig. 6). In the AmR and AcR groups, the differentially accumulated metabolites were predominantly enriched in isoflavonoid biosynthesis and flavone and flavonol biosynthesis. In the AmS and AcS groups, the differentially accumulated metabolites were predominantly enriched in isoflavonoid biosynthesis and flavone and flavonol biosynthesis. In the AmL and AcL groups, the differentially accumulated metabolites were predominantly enriched in flavone and flavonol biosynthesis, isoflavonoid biosynthesis and flavonoid biosynthesis. Some overlapping pathways, such as isoflavonoid biosynthesis and flavone and flavonol biosynthesis, were identified from the various comparisons. Flavonoid biosynthesis synthesizes upstream substances and provides the foundation for further pathways. These metabolic pathways are closely related to ongoing research.

In the AmS and AmR groups, the differentially accumulated metabolites were mainly enriched in phenylpropanoid biosynthesis, arginine and proline metabolism, flavonoid biosynthesis and ABC transporters. In the AcS and AcR groups, the differentially accumulated metabolites were mainly enriched in phenylpropanoid biosynthesis and isoflavonoid biosynthesis. Among these pathways, the flavonoid biosynthesis pathway and isoflavonoid biosynthesis pathway deserve attention.

## Discussion

Ac, which belongs to the genus *Abrus* of the subfamily Papilionoideae (Leguminous), is an important medicinal plant with economic value in southern China and was officially recorded in the Chinese Pharmacopoeia. Whole plants without pods can be used as Chinese herbal medicines and exhibit a variety of pharmacological effects, such as anti-inflammatory, hepatoprotective, hypolipidemic and immunomodulatory effects [44]. The high demand for *A. cantoniensis* in medicinal markets leads to its overexploitation and scarcity in germplasm resources, so candidate germplasm resources are urgently needed for enrichment and improvement. Within the same genus, Am exhibits a similar composition and efficacy and is often used as an alternative to Ac. Am is an important candidate resource for this objective. Before fully utilizing Am as an alternative resource, we performed widely targeted metabolomics to compare the secondary metabolites between Ac and Am, as the secondary metabolites of these compounds are essential for their pharmacological activity. In this study, we used UPLC‒ESI‒MS/MS to analyze the roots, stems and leaves of Am and Ac and detected a total of 635 metabolites in 8 classes. We performed a statistical analysis of the metabolites, and significant differences were observed between the different groups (AmR, AmS, AmL, AcR, AcS and AcL).

The basic structure of flavonoids includes a C15 benzene ring structure of C6-C3-C6, which is a natural organic compound widely found in plants and is abundant in many Chinese herbal medicines [27, 34]. In our study, we found a significant increase in the amounts of liquiritigenin, catechin, isoquercitrin, and other flavonoids in AcR compared with AmR. Research shows that liquiritigenin protects against ATO-induced hepatotoxicity because it exhibits antioxidant and anti-inflammatory activities and enhances autophagy, which is mediated by the PI3K/AKT/mTOR signaling pathway in mice [52]. Catechins exhibit a strong ability to neutralize reactive oxygen and nitrogen species. Different types of catechin derivatives include epicatechin, epigallocatechin, epicatechin gallate and epigallocatechin gallate [31]. Epicatechin and epigallocatechin were also detected in AcR and AmR. Zhang [50] showed that isoquercetin can improve the ability to exercise, improve energy metabolism, improve antioxidant capacity, reduce injury due to exercise, and produce a good anti-fatigue effect. In addition, we found significantly increased amounts of daidzein, formononetin, maackiain, oroxin A and other flavonoids in AmR compared with AcR. Daidzein plays a significant role in improving hyperglycemia, insulin resistance, dyslipidemia, obesity, inflammation, etc. [10]. Formononetin shows anti-inflammatory, antioxidant, antitumor and other effects [5]. Maackiain is an isoflavanone compound with antioxidant [53], anti-inflammatory [16], antibacterial [15], antitumor [49] and other pharmacological effects. Oroxin A exhibits antioxidant activities [25]. Oroxin A also has the ability to activate PPARγ and inhibit α-glucosidase and is a promising candidate for diabetes intervention [42].

Phenolic acids are a class of natural active products in plants, and their biosynthesis mainly includes the shikimic acid metabolic pathway and phenylpropanoid metabolic pathway [14, 22]. Studies have found that phenolic acids exhibit anti-inflammatory, antioxidant, antitumor, antibacterial and other pharmacological effects [13, 46]. In our study, we found significantly increased amounts of 4-phenylbutyric acid, methyl caffeate, salicin, verbasoside, and other phenolic acids in AcR compared with AmR. 4-Phenylbutyric acid, a short-chain aromatic fatty acid, is a potent inhibitor of endoplasmic reticulum stress [36]. Methyl caffeate is a specific strong inhibitor that can inhibit the production of senescence-associated secretory phenotypes [23]. Salicin is a potent anti-inflammatory agent that can inhibit tumor growth and angiogenesis, providing a new perspective for the effective treatment of hypervascular tumors [19]. Verbasoside (VER) is a phenylethanoid glycoside widely used in traditional medicine. VER showed antiproliferative effects in many types of cancer [4]. In addition, we found significantly increased amounts of caffeic acid, sinapic acid, and other phenolic acids in AmR compared with AcR. Caffeic acid is a natural antioxidant with a variety of pharmacological effects, such as antibacterial, anti-inflammatory and antiviral effects [28, 35]. Sinapic acid has been widely studied for its various pharmacological effects, particularly its anticancer effects [3]. A higher amount of verbascoside was found in AmL than in AcL. Verbascoside possesses activities for human health that are pharmacologically beneficial, including antioxidant, anti-inflammatory and antineoplastic properties in addition to numerous wound-healing and neuroprotective properties [2]. More kaempferide (3,5,7-trihydroxy-4'-methoxyflavone), which shows potent anticancer activity in a number of human tumor cell lines [33], was found in AcL than in AmL. High phenolic and flavonoid contents were found in AcR and AmR, which exhibited high antioxidant capacities. Studies have shown that compared to Am, Ac possesses stronger radical scavenging activities and higher reducing power [45]. Combined with previous studies, we hypothesize that the large differences in the contents of flavonoids and phenolic acids are closely related to clinical effects.

Other types of compounds also exhibit pharmacological activity. The amounts of abrine and hypaphorine in AmS are higher than those in AcS, which are alkaloids with various pharmacological activities, such as anti-inflammatory and antioxidant activities [25, 26]. AmR is rich in soyasaponins, which are triterpenoids with a variety of biological activities, such as antitumor [43], obesity prevention [18] and immunomodulation activity [11]. The different types and levels of these pharmacologically active metabolites may affect the pharmacological action and clinical effectiveness of Ac and Am.

The aboveground components of Ac also show rich biological activity. Phenolic acids were more abundant in AcS than in AcR, while triterpenoid saponins were more abundant in AcR, and the same phenomenon was observed in Am. Some of the findings are consistent with previously reported results [45]. This discovery was attributed to the differential expression of key enzyme genes in various tissues,these genes regulate the synthesis and accumulation of phenolic acids and triterpenoid saponins in different tissues [44]. Moreover, two metabolites (isovitexin-2''-xyloside, soyasaponin V) were unique to Ac and reported for the first time in *A. cantoniensis*. We can use these two compounds as chemical markers to identify Ac and Am.

The active ingredients of medicinal plants are the material basis for the efficacy of traditional Chinese medicines, and their accumulation shows strong spatial and temporal specificity [29]. For example, tanshinone, the main active ingredient of *Salvia miltiorrhiza*, mainly accumulates in the roots [6]. The spatial and temporal regulation of genes has an effect on the synthesis and accumulation of metabolites in different tissues of Am and Ac. Differences in metabolites between Ac and Am lead to differences in clinical efficacy. In this study, metabolomic analysis of different tissue parts of Ac and Am was performed to elucidate the patterns of metabolite accumulation in different tissue parts and the mechanism leading to herbal quality. The latest National Pharmacopeia of China only recommends AC for the use. Our study showed that certain groups of metabolites of AC and AM were significantly different. There are many herbs that come from a variety of source plants, such as *Bupleurum chinense* DC. and *Bupleurum scorzonerifolium* Willd. were included as the two authentic sources of Chinese traditional medicine Bupleuri Radix (BR, chaihu). Qu et al. indicated by metabolomic analysis that the pharmacological metabolites of Bs and Bc were significantly different. The correlation between the type or content of herbal components from different plant sources and the specificity and universality of efficacy still needs further pharmacological verification. So we can't recommend stopping replacing AC with AM based only on metabolite differences. Our study can provide a basis for subsequent studies on biosynthetic pathways and related bioactivities and can provide a reference to guide the clinical application of traditional Chinese medicine.

## Conclusions

The UPLC‒ESI‒MS/MS metabolomics method was employed in this study to assess the differences in metabolites between Ac and Am. This study comprises the metabolomics-based investigation comparing Ac and Am. A total of 635 metabolites (including 205 flavonoids, 200 phenolic acids, 79 alkaloids, 52 terpenoids, 40 lignans and coumarins, 13 tannins, 9 quinones, and other compounds) were detected. Wide differences in flavonoid and phenolic acid metabolites were found between the different samples, and both Ac and Am contain unique metabolites.

## Materials and methods

### Plant materials

In this study, 6 cultivated Ac and 6 cultivated Am (one year old) plants were collected at the same location in Pingnan County, Guangxi Province, China (longitude 110°30′2″ E latitude 23°43′22″ N; altitude 55 m above sea level), in January 2022. The habitat environments of the plants and cultivation measures were similar. The plant materials are kept in the Key Laboratory of Conservation and Utilization of Ethnic Medicine Resources of Guangxi University of Traditional Chinese medicine. After collection, the plants were washed twice—once with sterile water and once with running water. For further investigation, these samples were flash-frozen in liquid nitrogen containers and maintained at -80 ℃. The roots (R), stems (S) and leaves (L) of Ac and Am were named AcR and AcS, AcL and AmR, and AmS and AmL, respectively. Three biological replicates of each sample were separately examined.

### Sample preparation and extraction

The biological samples were freeze-dried with a vacuum freeze dryer (Scientz-100F). A mixer mill (MM 400, Retsch) with zirconia beads was used to crush the freeze-dried samples for 1.5 min at 30 Hz. One hundred milligrams of lyophilized powder was dissolved with 1.2 mL of 70% methanol solution. The samples were vortexed for 30 seconds every 30 min (6 times in total) and placed in a refrigerator at 4 °C overnight. The extracts were centrifuged at 12,000 rpm for 10 min, filtered (SCAA-104, pore size of 0.22 m), and then analyzed by UPLC‒MS/MS.

### UPLC conditions

To evaluate the sample extracts, a UPLC‒ESI‒MS/MS system was used (UPLC, SHIMADZU Nexera X2; MS, Applied Biosystems 4500 Q TRAP). The analytical conditions were as follows: UPLC, Agilent SB-C18 column (1.8 µm, 2.1 mm*100 mm); mobile phase, solvent A (pure water with 0.1% formic acid) and solvent B (acetonitrile with 0.1% formic acid); gradient elution, 95%-5% A at 0-9 min, 5% A at 9-10 min, 5%-95% A at 10 min-11.1 min, and 95% A at 11.1 min-14 min; flow rate, 0.35 mL per minute; temperature, 40 °C; and injection volume, 4 μL. The effluent was alternatively connected to an ESI-triple quadrupole-linear ion trap (QTRAP)-MS [51].

### ESI-Q TRAP-MS/MS

For metabolite detection, the AB4500 Q TRAP LC/MS/MS system with linear ion (LIT) and triple quadrupole (QQQ) sensors was used. The system was fitted with an ESI Turbo Ion-Spray interface, which was run in both the positive and negative ion modes and controlled by Analyst 1.6.3 software (AB Sciex). The ESI source operation parameters were as follows: ion source, turbo spray; source temperature, 550 ℃; ion spray voltage (IS), 5500 V (positive ion mode)/-4500 V (negative ion mode); gas I, gas II and curtain gas (CUR), 50, 60, and 25.0 psi, respectively; and collision-activated dissociation (CAD), high. In the QQQ and LIT modes, instrument tuning and mass calibration were conducted using 10 and 100 μmol/L polypropylene glycol solutions, respectively. QQQ scans were acquired as MRM experiments with the collision gas (nitrogen) set to medium. Through further declustering potential (DP) and collision energy (CE) optimization, the metamorphic potential (DP) and collision energy (CE) of a single MRM transition were determined. Depending on the metabolites eluted in each period, a specific set of MRM transitions was monitored [8].

### Qualitative and quantitative determination of metabolites

For qualitative analysis of metabolites, metabolites are annotated using primary and secondary MS data according to self-built MetWare databases (MWDB) and public metabolite databases. To ensure the accuracy of metabolite annotation, interfering signals, including repetitive signals of K^+^, Na^+^, and NH_4_^+^ ions, isotopic signals, and repetitive signals of fragment ions, were first excluded from the analysis.

For the quantitative analysis of metabolites, the multiple-reaction monitoring (MRM) mode of QQQ MS was employed. In MRM mode, the quadrupole filters the precursor ions of the target substance and excludes the ions corresponding to other molecular weights to eliminate interference. After obtaining the metabolite mass spectrometry data, peak area integration was performed using MultiQuant version 3.0.2 (AB SCIEX, Concord, ON, Canada). Finally, the chromatographic peak area was used to determine the relative metabolite contents.

### Multivariate statistical analysis

The metabolite data were log_2_-transformed for statistical analysis to improve their normality and normalized. Metabolites from 6 samples were used for hierarchical clustering analysis (HCA) within R (ComplexHeatmap 2.8.0). Unsupervised principal component analysis (PCA) was performed by the statistics function prcomp within R v3.5.0 (www.r-project.org). VIP values were extracted from the OPLS-DA results, which also contained score and permutation plots and were generated using the R package MetaboAnalystR. Venn diagrams were used to show the number of differentially accumulated metabolites. The identified metabolites were annotated with the KEGG Compound database, and the annotated metabolites were then mapped to the KEGG Pathway database.

## Supplementary Information

**Additional file 1.**

## Data Availability

All data generated or analyzed during this study are included in this published article.
