# Supplementary material for: Metabolomics analysis reveals the differences between Abrus cantoniensis Hance and Abrus mollis Hance
Source: BMC Plant Biol. 2023 Aug 1;23:375. doi: 10.1186/s12870-023-04372-y (PMC10391822; doi:10.1186/s12870-023-04372-y)
Supplement: Supplementary file 1 — Additional file 1. [file 12870_2023_4372_MOESM1_ESM.pdf]

Supplementary material

Metabolomics Analysis Reveals the Differences Between *Abrus cantoniensis* Hance and *Abrus mollis* Hance

Kexin Cao [a,b], Jianhua Chen [a], Rongshao Huang [a], Rumei Lu [a], Xiao Zhou [a], Yuanyuan Bu [a], Liangbo Li [a*], Chun Yao [a**]

*Corresponding author

E-mail addresses: llb100@126.com (L.-B. Li);

**Corresponding author

E-mail addresses: yaoc@gxtcmu.edu.cn (C. Yao)

Supplementary Figures and Tables
Supplementary Figures

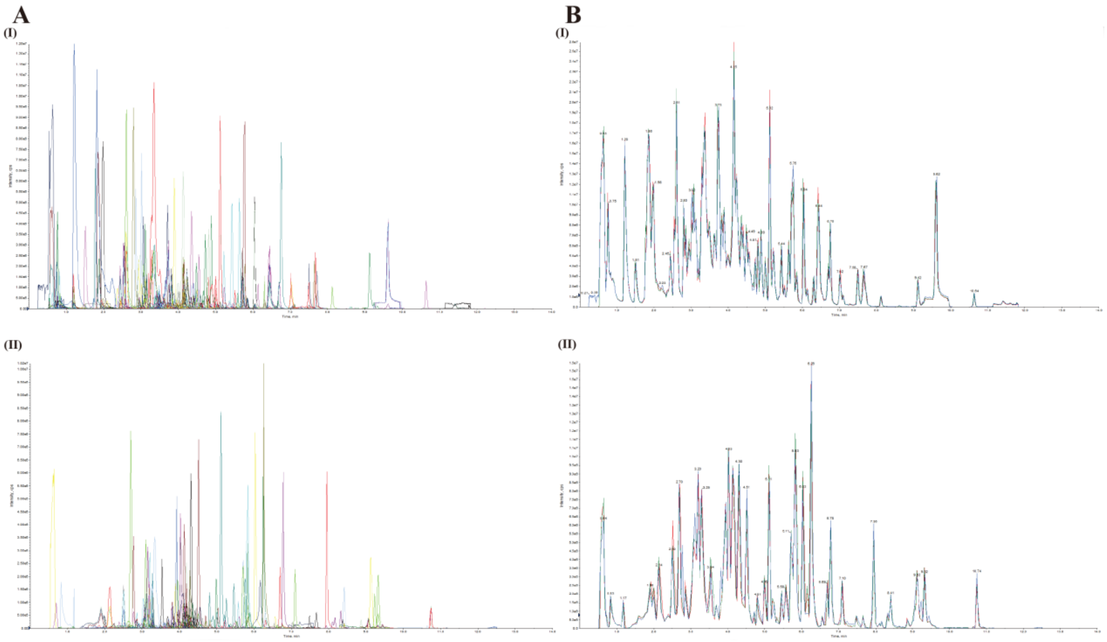

Supplementary Figure 1. (A) MRM metabolite detection multipeak map (multisubstance extraction ion current spectrum, XIC) Note: The abscissa is the retention time of the metabolite detection (retention time, Rt). The ordinate is the ion current intensity of the ion detection (the intensity units are counts per second (cps)). I is the detection in positive ion mode; II is the detection in negative ion mode; (B) Detection of the TIC overlap map by QC sample mass spectrometry.

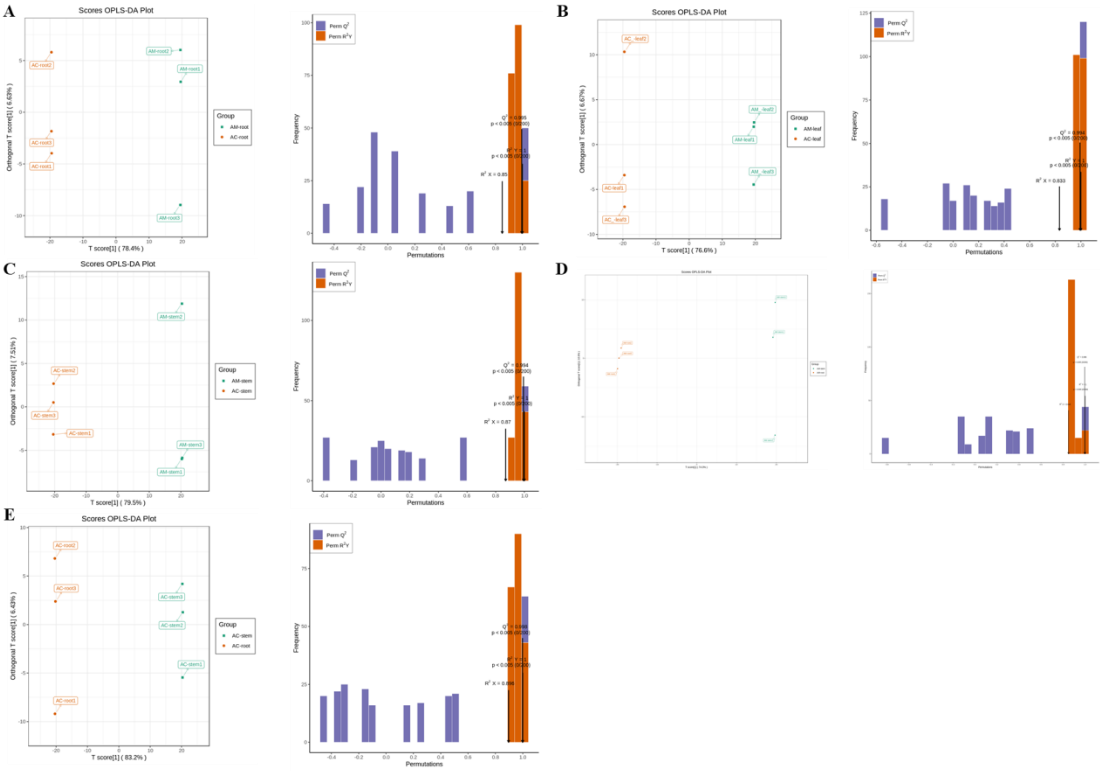

Supplementary Figure 2. The score plots and permutation test of the OPLS-DA model for the comparison of AmR vs AcR (A), AmL vs AcL (B), AmS vs AcS (C), AmS vs AmR (D), AcS vs AcR (E).

Supplementary Tables

Supplementary Table 1. Flavonoids identified from AC and AM

Formula	Q1 (Da)	Q3 (Da)	Molecular Weight (Da)	Ionization model	Compounds	Classification
C15H12O4	257.08	137.00	256.07	$[M+H]^+$	Isoliquiritigenin	Chalcones
C16H14O4	271.10	121.03	270.09	$[M+H]^+$	Echinatin	Chalcones
C16H14O4	271.10	167.03	270.09	$[M+H]^+$	Pinostrobin Chalcone	Chalcones
C21H22O9	419.13	257.09	418.13	$[M+H]^+$	Isoliquiritin	Chalcones
C21H24O10	435.13	315.09	436.14	$[M-H]^-$	Phloretin-4'-O-glucoside (Trilobatin)	Chalcones
C21H24O10	435.13	167.03	436.14	$[M-H]^-$	Phloretin-2'-O-glucoside (Phlorizin)	Chalcones
C15H12O5	271.06	151.00	272.07	$[M-H]^-$	Naringenin (5,7,4'-Trihydroxyflavanone)*	Dihydroflavone
C15H12O5	273.08	153.00	272.07	$[M+H]^+$	Butin	Dihydroflavone
C20H20O4	325.14	149.03	324.14	$[M+H]^+$	Glabranine	Dihydroflavone
C25H28O4	393.21	337.14	392.20	$[M+H]^+$	Glabrol	Dihydroflavone
C21H22O9	419.13	257.08	418.13	$[M+H]^+$	Liquiritigenin-4'-O-Glucoside (Liquiritin)	Dihydroflavone
C21H22O10	433.11	271.06	434.12	$[M-H]^-$	Butin-7-O-glucoside*	Dihydroflavone
C21H22O10	433.11	271.00	434.12	$[M-H]^-$	Naringenin-7-O-glucoside (Prunin)	Dihydroflavone
C21H22O10	433.12	271.06	434.12	$[M-H]^-$	Naringenin-4'-O-glucoside*	Dihydroflavone
C26H30O6	439.21	303.16	438.20	$[M+H]^+$	Leachianone A	Dihydroflavone
C21H22O11	451.12	289.07	450.12	$[M+H]^+$	3',5,5',7-Tetrahydroxyflavanone-7-O-glucoside	Dihydroflavone
C21H22O11	451.12	289.00	450.12	$[M+H]^+$	Eriodictyol-7-O-glucoside	Dihydroflavone
C24H24O13	521.00	273.00	520.12	$[M+H]^+$	Naringenin-7-O-(6"-malonyl)glucoside	Dihydroflavone
C27H32O14	581.19	419.13	580.18	$[M+H]^+$	Liquiritigenin-7,4'-O-diglucoside	Dihydroflavone
C27H32O14	581.19	273.08	580.18	$[M+H]^+$	Naringenin-7-O-Rutinoside(Narirutin)	Dihydroflavone

C28H34O14	595.20	287.09	594.20	$[M+H]^+$	Didymin (Isosakuranetin-7-O-rutinoside)	Dihydroflavone
C27H32O15	595.17	287.06	596.17	$[M-H]^-$	Eriodictyol-7-O-Rutinoside (Eriocitrin)	Dihydroflavone
C15H12O5	271.06	151.00	272.07	$[M-H]^-$	Pinobanksin*	Dihydroflavonol
C22H24O11	463.12	301.00	464.13	$[M-H]^-$	Hesperetin-5-O-glucoside	Dihydroflavonol
C16H16O4	271.10	147.02	272.11	$[M-H]^-$	(3R)-Vestitol	Dihydroisoflavone
C17H14O7	329.07	299.02	330.07	$[M-H]^-$	5,7-Dihydroxy-2'-methoxy-3',4'-methyleneoxydihydroisoflavone	Dihydroisoflavone
C18H18O7	345.10	315.05	346.11	$[M-H]^-$	5,7-Dihydroxy-2',3',4'-trimethoxydihydroisoflavone	Dihydroisoflavone
C15H14O5	275.09	107.00	274.08	$[M+H]^+$	Epiafzelechin	Flavanols
C15H14O6	291.09	139.04	290.08	$[M+H]^+$	Catechin	Flavanols
C15H14O6	291.09	139.04	290.08	$[M+H]^+$	Epicatechin	Flavanols
C15H14O6	289.07	151.04	290.08	$[M-H]^-$	5,7,3',4',5'-Pentahydroxyflavan (Tricetiflavan)	Flavanols
C17H16O5	299.10	223.10	300.10	$[M-H]^-$	4'-Hydroxy-5,7-dimethoxyflavanone	Flavanols
C16H16O6	305.10	139.04	304.10	$[M+H]^+$	3'-O-Methyl-(-)-epicatechin	Flavanols
C15H14O7	307.08	139.04	306.07	$[M+H]^+$	Epigallocatechin	Flavanols
C24H20O9	451.10	341.06	452.11	$[M-H]^-$	Catechin-(7,8-bc)-4β-(3,4-dihydroxyphenyl)-dihydro-2-(3H)-one	Flavanols
C21H24O11	451.12	289.07	452.13	$[M-H]^-$	Epicatechin glucoside	Flavanols
C30H26O11	561.14	289.20	562.15	$[M-H]^-$	Epicatechin-epiafzelechin	Flavanols
C31H28O12	593.17	441.11	592.16	$[M+H]^+$	8,8'-Methylenebiscatechin	Flavanols
C45H38O18	865.20	407.10	866.21	$[M-H]^-$	Catechin-catechin-catechin	Flavanols
C15H10O4	253.05	117.00	254.06	$[M-H]^-$	7,4'-Dihydroxyflavone	Flavonoid
C15H10O4	255.07	137.02	254.06	$[M+H]^+$	6,7-Dihydroxyflavone	Flavonoid
C15H12O4	257.08	137.02	256.07	$[M+H]^+$	Liquiritigenin	Flavonoid
C15H12O4	257.08	137.02	256.07	$[M+H]^+$	2,4,4'-trihydroxychalcone	Flavonoid
C15H10O5	271.06	243.06	270.05	$[M+H]^+$	3',4',7-Trihydroxyflavone	Flavonoid
C15H10O5	271.06	153.01	270.05	$[M+H]^+$	Apigenin	Flavonoid

C16H10O5	283.06	225.05	282.05	$[M+H]^+$	pseudobaptigenin	Flavonoid
C16H12O5	285.08	270.00	284.07	$[M+H]^+$	Genkwanin (Apigenin 7-methyl ether)	Flavonoid
C16H12O5	285.08	270.00	284.07	$[M+H]^+$	Acacetin	Flavonoid
C15H10O6	285.04	151.00	286.05	$[M-H]^-$	Luteolin (5,7,3',4'-Tetrahydroxyflavone)	Flavonoid
C17H12O5	297.08	267.06	296.07	$[M+H]^+$	7-O-methylpseudobaptigenin	Flavonoid
C16H12O6	301.07	286.08	300.06	$[M+H]^+$	Chrysoeriol*	Flavonoid
C16H12O6	301.07	286.05	300.06	$[M+H]^+$	Hispidulin (5,7,4'-Trihydroxy-6-methoxyflavone)*	Flavonoid
C20H18O5	339.12	283.06	338.12	$[M+H]^+$	Licoflavone C	Flavonoid
C21H18O10	431.10	255.07	430.09	$[M+H]^+$	Chrysin-7-O-Glucuronide	Flavonoid
C21H20O10	431.10	269.10	432.11	$[M-H]^-$	Apigenin-7-O-glucoside(Cosmosiin)	Flavonoid
C21H20O10	433.11	271.07	432.11	$[M+H]^+$	Apigenin-7-O-α-D-glucoside	Flavonoid
C21H20O10	433.11	271.08	432.11	$[M+H]^+$	Oroxin A	Flavonoid
C21H20O10	433.11	271.01	432.11	$[M+H]^+$	Apigenin-5-O-glucoside	Flavonoid
C21H20O11	449.11	287.20	448.10	$[M+H]^+$	Luteolin-7-O-glucoside (Cynaroside)	Flavonoid
C21H20O11	449.11	287.06	448.10	$[M+H]^+$	Luteolin-3'-O-glucoside	Flavonoid
C21H20O11	449.11	287.06	448.10	$[M+H]^+$	Kaempferol-4'-O-glucoside	Flavonoid
C21H22O11	449.10	287.05	450.12	$[M-H]^-$	Aromadendrin-7-O-glucoside	Flavonoid
C22H22O11	461.11	299.20	462.12	$[M-H]^-$	Chrysoeriol-7-O-glucoside*	Flavonoid
C22H22O11	463.12	301.07	462.12	$[M+H]^+$	Diosmetin-7-O-galactoside*	Flavonoid
C22H22O11	463.12	301.07	462.12	$[M+H]^+$	Diosmetin-7-O-glucoside*	Flavonoid
C22H22O11	463.11	343.08	462.12	$[M+H]^+$	Chrysoeriol-8-C-glucoside (Scoparin)	Flavonoid
C22H22O11	463.13	301.07	462.12	$[M+H]^+$	Hispidulin-7-O-Glucoside*	Flavonoid
C21H20O12	465.10	303.05	464.10	$[M+H]^+$	Isoquercitrin	Flavonoid
C23H22O11	475.12	271.06	474.12	$[M+H]^+$	Apigenin-7-O-(6"-acetyl)glucoside	Flavonoid
C22H22O12	479.12	317.06	478.11	$[M+H]^+$	Nepetin-7-O-glucoside	Flavonoid
C22H24O12	481.13	319.05	480.13	$[M+H]^+$	3',5',5,7-Tetrahydroxy-4'-methoxyflavanone-3'-O-glucoside	Flavonoid

C23H24O12	493.13	331.08	492.13	$[M+H]^+$	Tricin-7-O-Glucoside	Flavonoid
C24H24O12	505.12	257.08	504.13	$[M+H]^+$	Pinocembrin-7-O-(6"-O-malonyl)glucoside	Flavonoid
C23H24O13	509.13	347.08	508.12	$[M+H]^+$	5,6,3',4'-Tetrahydroxy-3,7-dimethoxyflavone-6-O-glucoside	Flavonoid
C24H22O13	519.11	433.11	518.11	$[M+H]^+$	Apigenin-7-O-(6"-malonyl)glucoside	Flavonoid
C23H22O14	521.09	329.20	522.10	$[M-H]^-$	Tricin-7-O-saccharic acid	Flavonoid
C24H22O14	535.11	449.10	534.10	$[M+H]^+$	Luteolin-7-O-(6"-malonyl)glucoside	Flavonoid
C25H24O14	549.12	301.07	548.12	$[M+H]^+$	Diosmetin-7-O-(6"-malonyl)glucoside*	Flavonoid
C25H24O14	549.12	301.40	548.12	$[M+H]^+$	Chrysoeriol-7-O-(6"-malonyl)glucoside*	Flavonoid
C25H24O14	549.12	301.07	548.12	$[M+H]^+$	Kaempferide-3-O-(6"-malonyl)glucoside	Flavonoid
C25H24O15	565.12	317.08	564.09	$[M+H]^+$	Isorhamnetin-3-O-(6"-malonylglucoside)	Flavonoid
C26H28O14	565.16	271.60	564.15	$[M+H]^+$	Apigenin-7-O-(2"-glucosyl)arabinoside	Flavonoid
C30H26O12	579.15	271.06	578.14	$[M+H]^+$	Apigenin-7-O-(6"-p-Coumaryl)glucoside	Flavonoid
C27H30O14	579.17	271.01	578.16	$[M+H]^+$	Apigenin-7-O-rutinoside (Isorhoifolin)	Flavonoid
C26H28O15	581.15	287.06	580.14	$[M+H]^+$	Kaempferol-3-O-sambubioside	Flavonoid
C27H30O15	595.17	449.10	594.16	$[M+H]^+$	Luteolin-7-O-rutinoside*	Flavonoid
C27H30O15	595.17	449.10	594.16	$[M+H]^+$	Luteolin-7-O-neohesperidoside (Lonicerin)*	Flavonoid
C27H30O15	595.17	457.20	594.16	$[M+H]^+$	Apigenin-6,8-di-C-glucoside (Vicenin-2)	Flavonoid
C27H30O15	593.15	285.04	594.16	$[M-H]^-$	Kaempferol-3-O-rutinoside(Nicotiflorin)*	Flavonoid
C28H32O15	609.18	301.07	608.17	$[M+H]^+$	Diosmetin-7-O-rutinoside (Diosmin)*	Flavonoid
C28H32O15	609.18	301.07	608.17	$[M+H]^+$	Diosmetin-7-O-Neohesperidoside (Neodiosmin)*	Flavonoid
C31H28O13	609.18	463.08	608.18	$[M+H]^+$	Hispidulin-7-O-(6"-O-p-Coumaroyl)Glucoside	Flavonoid
C27H30O16	611.16	287.06	610.15	$[M+H]^+$	Luteolin-7,3'-di-O-glucoside	Flavonoid
C27H30O16	611.16	287.05	610.15	$[M+H]^+$	Luteolin-7-O-gentiobioside	Flavonoid
C27H30O16	611.16	449.11	610.15	$[M+H]^+$	Orientin-2"-O-galactoside	Flavonoid
C28H32O16	625.18	317.06	624.16	$[M+H]^+$	Isorhamnetin-3-O-rutinoside (Narcissin)	Flavonoid
C28H32O16	625.17	301.07	624.17	$[M+H]^+$	Chrysoeriol-5,7-di-O-glucoside	Flavonoid

C29H34O18	671.18	347.07	670.18	$[M+H]^+$	Limocitrin-3,7-di-O-glucoside	Flavonoid
C30H32O18	681.17	287.05	680.16	$[M+H]^+$	Luteolin-7-O-(6"-malonyl)glucoside-5-O-rhamnoside	Flavonoid
C33H40O21	773.22	611.17	772.21	$[M+H]^+$	Kaempferol-6,8-di-C-glucoside-7-O-glucoside	Flavonoid
C20H18O10	419.10	383.07	418.09	$[M+H]^+$	Luteolin-8-C-arabinoside	Flavonoid carbonoside
C26H28O14	565.16	529.13	564.12	$[M+H]^+$	Apigenin-6-C-glucoside-8-C-arabinoside (Schaftoside)	Flavonoid carbonoside
C26H28O14	565.16	409.09	564.12	$[M+H]^+$	Apigenin-6-C-arabinoside-8-C-glucoside (Isoschaftoside)	Flavonoid carbonoside
C26H28O14	565.16	433.11	564.15	$[M+H]^+$	"Vitexin-2""-O-xyloside"	Flavonoid carbonoside
C26H28O14	565.15	433.11	564.15	$[M+H]^+$	Isovitexin-2"xyloside	Flavonoid carbonoside
C26H28O14	565.16	433.11	564.15	$[M+H]^+$	Apigenin-6-C-(2"-xylosyl)glucoside	Flavonoid carbonoside
C27H30O14	579.17	433.00	578.16	$[M+H]^+$	Vitexin-2"-O-rhamnoside	Flavonoid carbonoside
C27H30O15	595.17	577.16	594.16	$[M+H]^+$	Vitexin-2"-O-glucoside	Flavonoid carbonoside
C27H30O15	595.17	433.12	594.16	$[M+H]^+$	"Vitexin-2""-O-galactoside"	Flavonoid carbonoside
C27H30O16	611.16	593.14	610.15	$[M+H]^+$	Luteolin-6,8-di-C-glucoside	Flavonoid carbonoside
C27H30O16	611.16	465.10	610.15	$[M+H]^+$	Orientin-7-O-glucoside	Flavonoid carbonoside
C36H36O17	741.20	433.11	740.20	$[M+H]^+$	Isovitexin-2"-O-(6'''-p-coumaroyl)glucoside	Flavonoid

						carbonoside
C16H12O6	301.07	258.05	300.06	$[M+H]^+$	Kaempferide (3,5,7-Trihydroxy-4'-methoxyflavone)	Flavonols
C16H12O7	315.05	271.02	316.06	$[M-H]^-$	3-O-Methylquercetin	Flavonols
C21H20O11	449.11	287.06	448.10	$[M+H]^+$	Amoenin	Flavonols
C21H20O11	449.11	287.06	448.10	$[M+H]^+$	Kaempferol-3-O-glucoside (Astragalin)*	Flavonols
C21H20O11	447.09	285.00	448.10	$[M-H]^-$	Kaempferol-3-O-galactoside (Trifolin)	Flavonols
C21H20O11	447.09	285.04	448.10	$[M-H]^-$	Kaempferol-7-O-glucoside*	Flavonols
C21H20O12	463.09	300.03	464.10	$[M-H]^-$	Quercetin-3-O-glucoside (Isoquercitrin)*	Flavonols
C21H20O12	463.09	300.70	464.10	$[M-H]^-$	Quercetin-7-O-glucoside*	Flavonols
C22H22O12	479.12	317.06	478.11	$[M+H]^+$	Isorhamnetin-7-O-glucoside (Brassicin)	Flavonols
C23H22O12	491.12	287.05	490.11	$[M+H]^+$	Kaempferol-3-O-(2"-acetyl)glucoside	Flavonols
C23H22O12	489.11	285.04	490.11	$[M-H]^-$	Kaempferol-3-O-(6"-acetyl)glucoside	Flavonols
C23H20O13	503.08	459.10	504.09	$[M-H]^-$	Kaempferol-3-O-(2"-O-acetyl)glucuronide	Flavonols
C24H22O14	535.11	287.05	534.10	$[M+H]^+$	Kaempferol-3-O-(6"-malonyl)galactoside*	Flavonols
C24H22O14	535.11	287.05	534.10	$[M+H]^+$	Kaempferol-3-O-(6"-malonyl)glucoside*	Flavonols
C24H22O15	551.10	303.05	550.10	$[M+H]^+$	Quercetin-7-O-(6"-malonyl)glucoside	Flavonols
C26H28O14	565.15	287.05	564.15	$[M+H]^+$	Kaempferol-3-O-arabinoside-7-O-rhamnoside	Flavonols
C27H30O15	595.17	287.06	594.16	$[M+H]^+$	Kaempferol-3-O-glucoside-7-O-rhamnoside	Flavonols
C27H30O15	593.15	285.00	594.16	$[M-H]^-$	Kaempferol-3-O-robinobioside(Biorobin)	Flavonols
C27H30O15	595.17	287.05	594.16	$[M+H]^+$	Kaempferol-3-O-neohesperidoside*	Flavonols
C27H30O16	609.15	301.00	610.15	$[M-H]^-$	Quercetin-3-O-rutinoside (Rutin)	Flavonols
C27H30O16	609.15	300.00	610.15	$[M-H]^-$	Quercetin-3-O-robinobioside	Flavonols
C27H30O17	627.16	303.04	626.15	$[M+H]^+$	Quercetin-3-O-sophoroside (Baimaside)	Flavonols
C28H32O17	641.17	317.07	640.16	$[M+H]^+$	Isorhamnetin-3-O-sophoroside	Flavonols
C33H40O20	757.22	287.06	756.21	$[M+H]^+$	Kaempferol-3-O-sophoroside-7-O-rhamnoside	Flavonols
C33H40O20	757.22	287.06	756.21	$[M+H]^+$	Kaempferol-3-O-neohesperidoside-7-O-glucoside	Flavonols

C34H42O21	787.23	317.06	786.22	$[M+H]^+$	Isorhamnetin-3-O-rutinoside-4'-O-glucoside	Flavonols
C34H42O21	787.23	317.07	786.22	$[M+H]^+$	Isorhamnetin-3-O-sophoroside-7-O-rhamnoside	Flavonols
C15H14O3	243.10	133.06	242.09	$[M+H]^+$	(±)-Equol; 7,4'-Homoisoflavane	Isoflavones
C15H10O4	255.07	199.08	254.06	$[M+H]^+$	Daidzein	Isoflavones
C15H12O4	255.07	119.05	256.07	$[M-H]^-$	3,9-Dihydroxypterocarpan	Isoflavones
C16H12O4	267.07	252.00	268.07	$[M-H]^-$	Formononetin (7-Hydroxy-4'-methoxyisoflavone)	Isoflavones
C16H12O4	269.08	254.05	268.07	$[M+H]^+$	Isoformononetin	Isoflavones
C15H10O5	271.06	215.07	270.05	$[M+H]^+$	Genistein	Isoflavones
C15H10O5	271.06	201.00	270.05	$[M+H]^+$	2'-Hydroxydaidzein	Isoflavones
C17H14O4	283.10	268.07	282.09	$[M+H]^+$	7,4'-Di-O-methyldaidzein	Isoflavones
C16H12O5	285.08	270.00	284.07	$[M+H]^+$	Biochanin A*	Isoflavones
C16H12O5	283.06	268.00	284.07	$[M-H]^-$	Prunetin (5,4'-Dihydroxy-7-methoxyisoflavone)*	Isoflavones
C16H12O5	285.08	229.08	284.07	$[M+H]^+$	3'-Methoxydaidzein	Isoflavones
C16H12O5	285.08	270.00	284.07	$[M+H]^+$	Calycosin	Isoflavones
C16H12O5	283.06	268.00	284.07	$[M-H]^-$	Glycitein	Isoflavones
C16H12O5	283.06	253.80	284.07	$[M-H]^-$	Maackiain	Isoflavones
C15H10O6	287.06	153.02	286.05	$[M+H]^+$	2'-Hydroxygenistein	Isoflavones
C15H10O6	287.06	241.00	286.05	$[M+H]^+$	Isoluteolin (Orobol)(5,7,3',4'-tetrahydroxyisoflavone)	Isoflavones
C15H12O6	287.06	125.02	288.06	$[M-H]^-$	2-Hydroxy-2,3-dihydrogenistein	Isoflavones
C15H12O6	289.07	243.07	288.06	$[M+H]^+$	2,6,7,4'-Tetrahydroxyisoflavanone	Isoflavones
C17H14O5	299.09	284.07	298.08	$[M+H]^+$	Afrormosin (6,4'-Dimethoxy-7-Hydroxyisoflavone)	Isoflavones
C17H14O5	297.08	282.05	298.08	$[M-H]^-$	Pterocarpine	Isoflavones
C16H12O6	299.06	284.02	300.06	$[M-H]^-$	Aracarpene 2	Isoflavones
C16H12O6	299.06	284.02	300.06	$[M-H]^-$	Aracarpene 1	Isoflavones
C16H12O6	301.07	286.04	300.06	$[M+H]^+$	Pratensein	Isoflavones
C16H12O6	301.07	258.05	300.06	$[M+H]^+$	Cajanin	Isoflavones

$C_{17}H_{16}O_6$	317.10	167.03	316.09	$[M+H]^+$	Cajanol	Isoflavones
$C_{20}H_{16}O_4$	321.11	137.02	320.11	$[M+H]^+$	Corylin	Isoflavones
$C_{20}H_{16}O_5$	337.11	281.08	336.10	$[M+H]^+$	Glabrone	Isoflavones
$C_{20}H_{18}O_5$	339.12	321.10	338.12	$[M+H]^+$	Psoralenol	Isoflavones
$C_{21}H_{20}O_5$	351.12	292.99	352.13	$[M-H]^-$	Gancaonin G	Isoflavones
$C_{18}H_{16}O_8$	361.09	346.07	360.09	$[M+H]^+$	5,7,4'-trihydroxy-6,3',5'-trimethoxyisoflavone	Isoflavones
$C_{21}H_{20}O_6$	369.13	313.07	368.13	$[M+H]^+$	Glisoflavone	Isoflavones
$C_{21}H_{20}O_9$	417.12	297.10	416.11	$[M+H]^+$	Puerarin	Isoflavones
$C_{21}H_{20}O_9$	417.12	255.07	416.11	$[M+H]^+$	Daidzein-7-O-glucoside(Daidzin)	Isoflavones
$C_{22}H_{22}O_9$	431.13	269.08	430.13	$[M+H]^+$	Formononetin-7-O-glycoside (Ononin)	Isoflavones
$C_{21}H_{20}O_{10}$	431.10	269.00	432.11	$[M-H]^-$	Genistein-7-O-Glucoside (Genistin)	Isoflavones
$C_{21}H_{20}O_{10}$	433.11	271.06	432.11	$[M+H]^+$	Sophoricoside	Isoflavones
$C_{21}H_{20}O_{10}$	433.11	283.06	432.11	$[M+H]^+$	Genistein-8-C-glucoside	Isoflavones
$C_{22}H_{22}O_{10}$	447.13	285.08	446.12	$[M+H]^+$	Glycitin	Isoflavones
$C_{22}H_{22}O_{10}$	447.13	285.07	446.12	$[M+H]^+$	Calycosin-7-O-glucoside	Isoflavones
$C_{22}H_{22}O_{10}$	447.13	285.07	446.12	$[M+H]^+$	3'-Methoxydaidzin	Isoflavones
$C_{22}H_{22}O_{10}$	447.13	285.00	446.12	$[M+H]^+$	Biochanin A-7-O-glucoside (Sissotrin)	Isoflavones
$C_{22}H_{22}O_{10}$	447.13	285.07	446.12	$[M+H]^+$	Prunetin-5-O-glucoside	Isoflavones
$C_{22}H_{22}O_{10}$	445.11	283.00	446.12	$[M-H]^-$	Trifolirhizin (Maackiain-3-O-glucoside)	Isoflavones
$C_{23}H_{22}O_{10}$	459.13	255.07	458.12	$[M+H]^+$	6''-O-Acetyldaidzin	Isoflavones
$C_{23}H_{24}O_{10}$	461.14	299.09	460.14	$[M+H]^+$	6,4'-Dimethoxyisoflavone-7-O-glucoside (Wistin)	Isoflavones
$C_{23}H_{22}O_{11}$	475.12	271.06	474.12	$[M+H]^+$	6''-O-Acetylgenistin	Isoflavones
$C_{24}H_{24}O_{11}$	489.14	285.09	488.13	$[M+H]^+$	6''-O-Acetylglycitin	Isoflavones
$C_{24}H_{22}O_{12}$	503.12	255.06	502.11	$[M+H]^+$	6''-O-Malonyldaidzin	Isoflavones
$C_{28}H_{24}O_9$	505.16	255.07	504.14	$[M+H]^+$	Daidzein-7-O-(2''-benzoyl)rhamnoside	Isoflavones
$C_{25}H_{24}O_{12}$	517.13	269.09	516.13	$[M+H]^+$	Formononetin-7-O-(6''-Malonyl)glucoside	Isoflavones

C24H22O13	519.11	271.06	518.11	$[M+H]^+$	Genistein-7-O-(6"-malonyl)glucoside	Isoflavones
C24H22O13	519.11	271.05	518.11	$[M+H]^+$	6"-O-Malonylgenistin	Isoflavones
C25H24O13	533.13	285.07	532.12	$[M+H]^+$	6"-O-Malonylglycitin	Isoflavones
C26H28O13	549.16	255.07	548.15	$[M+H]^+$	Daidzein-7-O-apiosyl(1→6)glucoside	Isoflavones
C26H28O13	549.16	255.07	548.15	$[M+H]^+$	Daidzein-7-O-Glucoside-4'-O-Apioside	Isoflavones
C27H30O13	563.18	269.08	562.17	$[M+H]^+$	Glycyroside	Isoflavones
C20H20O5	341.14	267.07	340.13	$[M+H]^+$	Kushenol S	Other Flavonoids
C25H26O4	391.19	335.13	390.18	$[M+H]^+$	Isolicoflavone B	Other Flavonoids
C25H26O6	423.18	299.06	422.17	$[M+H]^+$	Kushenol F	Other Flavonoids
C26H30O5	423.22	303.16	422.21	$[M+H]^+$	Kushenol U	Other Flavonoids
C26H30O6	439.21	315.09	438.20	$[M+H]^+$	Kushenol D	Other Flavonoids
C26H30O6	439.21	315.09	438.20	$[M+H]^+$	Kushenol Ca	Other Flavonoids
C27H32O6	453.23	329.10	452.22	$[M+H]^+$	Kushenol Da	Other Flavonoids
C27H30O13	563.18	431.13	562.17	$[M+H]^+$	Kushenol O	Other Flavonoids
C15H10O6	287.06	153.01	286.05	$[M+H]^+$	Aureusidin	Sinensetin
C21H20O11	449.11	287.06	448.10	$[M+H]^+$	Aureusidin-4-O-glucoside	Sinensetin

Supplementary Table 2. Alkaloids identified from AC and AM

Formula	Q1 (Da)	Q3 (Da)	Molecular Weight (Da)	Ionization model	Compounds	Classification
C15H24N2O3	281.19	263.17	280.18	$[M+H]^+$	(+)-5α,9α-Dihydroxymatrine	Alkaloids
C20H23N7O7	474.17	327.00	473.17	$[M+H]^+$	10-Formyltetrahydrofuran	Alkaloids
C11H12N2O2	205.10	146.06	204.09	$[M+H]^+$	1-Methoxy-indole-3-acetamide	Alkaloids
C5H9N3O2	144.08	84.06	143.07	$[M+H]^+$	2-Amino-4,5-dihydro-1H-imidazole-4-acetic acid	Alkaloids
C8H9NO	136.08	91.06	135.07	$[M+H]^+$	2'-Aminoacetophenone	Alkaloids
C10H9NO3	192.07	146.06	191.06	$[M+H]^+$	2-Oxo-3,4-dihydro-1H-quinoline-3-carboxylic acid	Alkaloids
C8H12N2O2	169.10	134.00	168.09	$[M+H]^+$	4-(Aminomethyl)-5-(hydroxymethyl)-2-methylpyridin-3-ol	Alkaloids
$C14H20NO3^+$	250.14	147.00	250.14	$[M]^+$	4-Coumaroylcholine	Alkaloids
C6H9NOS	144.05	113.20	143.04	$[M+H]^+$	4-Methyl-5-thiazoleethanol	Alkaloids
C11H21NO2	200.17	140.14	199.16	$[M+H]^+$	5-(2-Hydroxypropyl)-hygrine	Alkaloids
C15H22N2O	247.18	179.15	246.17	$[M+H]^+$	5,6-dehydrolupanine	Alkaloids
C15H24N2O3	281.19	245.17	280.18	$[M+H]^+$	5,9-dihydroxymatrine	Alkaloids
C15H22N2O2	263.18	245.16	262.17	$[M+H]^+$	5α-Hydroxysophocarpine	Alkaloids
C8H15NO3	174.11	114.09	173.11	$[M+H]^+$	6-Acetamidohexanoic acid	Alkaloids
C6H13NO2	132.10	57.10	131.10	$[M+H]^+$	6-Deoxyfagomine	Alkaloids
C15H22N2O2	263.18	245.17	262.17	$[M+H]^+$	9α-Hydroxysophocarpine	Alkaloids
C15H20N2O2	261.16	243.15	260.15	$[M+H]^+$	9α-Hydroxysophoramine	Alkaloids
C12H14N2O2	219.11	132.08	218.11	$[M+H]^+$	Abrine	Alkaloids
C36H34N2O10	655.23	474.15	654.22	$[M+H]^+$	Abrusamide A	Alkaloids
C36H34N2O10	655.23	262.07	654.22	$[M+H]^+$	Abrusamide B	Alkaloids
C6H6N2O	123.05	81.04	122.05	$[M+H]^+$	Acetylpyrazine	Alkaloids
C5H11NO2	118.09	58.07	117.08	$[M+H]^+$	Betaine	Alkaloids

C5H13NO	104.10	60.10	103.09	$[M+H]^+$	Choline	Alkaloids
C6H11NO4	162.08	98.00	161.07	$[M+H]^+$	DL-2-Aminoadipic acid	Alkaloids
C6H11N3O	142.10	124.09	141.09	$[M+H]^+$	Histidinol	Alkaloids
C14H18N2O2	247.14	146.06	246.14	$[M+H]^+$	Hypaphorine	Alkaloids
C16H14O4	271.10	166.90	270.09	$[M+H]^+$	Isomethacin	Alkaloids
C15H22N2O	247.18	179.16	246.17	$[M+H]^+$	Isosophocarpine	Alkaloids
C11H11NO4	222.08	130.06	221.07	$[M+H]^+$	Methyl dioxindole-3-acetate	Alkaloids
C9H11NO3	182.08	91.05	181.07	$[M+H]^+$	N-(2-Hydroxy-4-methoxyphenyl)acetamide	Alkaloids
C13H22N2	205.17	189.13	206.18	$[M-H]^-$	N,N'-dicyclohexylcarbodiimide	Alkaloids
C15H21NO7	328.13	310.12	327.13	$[M+H]^+$	N-benzoyl-2-aminoethyl-β-D-glucoside	Alkaloids
C8H9NO	136.07	91.05	135.07	$[M+H]^+$	N-benzylformamide	Alkaloids
C8H9N	120.08	103.05	119.07	$[M+H]^+$	N-Benzylmethylene isomethylamine	Alkaloids
C6H11NO3	146.08	70.06	145.07	$[M+H]^+$	N-Hydroxypipecolic acid	Alkaloids
C7H7NO2	138.05	78.00	137.05	$[M+H]^+$	Nicotinic Acid Methyl Ester(Methyl Nicotinate)	Alkaloids
C20H39NO2	326.31	62.06	325.30	$[M+H]^+$	N-Oleoylethanolamine	Alkaloids
$C5H15NO4P^+$	184.00	125.00	184.07	$[M]^+$	O-Phosphocholine	Alkaloids
C8H11NO	138.09	121.10	137.08	$[M+H]^+$	Phenylethanolamine	Alkaloids
C17H17NO3	284.13	147.04	283.12	$[M+H]^+$	P-Hydroxycinnamic acid p-hydroxyphenethylamine	Alkaloids
C5H11N	86.10	69.20	85.09	$[M+H]^+$	Piperidine	Alkaloids
C16H16O5	287.09	155.00	288.10	$[M-H]^-$	Shikonin	Alkaloids
C15H22N2O	247.18	136.00	246.17	$[M+H]^+$	Sophocarpine	Alkaloids
C15H20N2O	246.42	98.10	244.16	$[M+H]^+$	Sophoramine	Alkaloids
C15H24N2O	249.20	150.00	248.19	$[M+H]^+$	Sophoridine	Alkaloids
C15H24N2O2	265.04	148.11	264.18	$[M+H]^+$	Sophoridine N-oxide	Alkaloids
C7H8N4O2	181.07	138.07	180.07	$[M+H]^+$	Theobromine	Alkaloids
C7H7NO2	138.06	94.07	137.05	$[M+H]^+$	Trigonelline	Alkaloids

C8H17NO	144.14	57.07	143.13	$[M+H]^+$	Valpromide	Alkaloids
C10H16O7N4	305.11	143.06	304.10	$[M+H]^+$	Vicine	Alkaloids
C3H6N2	71.06	54.03	70.05	$[M+H]^+$	β-Aminopropionitrile	Alkaloids
C9H7N	130.06	103.05	129.06	$[M+H]^+$	Isoquinoline	Isoquinoline alkaloids
C11H9O2N	188.07	118.06	187.06	$[M+H]^+$	3-amino-2-naphthoic acid	Phenolamine
C5H14N2	103.12	86.10	102.12	$[M+H]^+$	Cadaverine	Phenolamine
C14H20N4O3	293.16	163.04	292.15	$[M+H]^+$	Caffeoylagmatine	Phenolamine
C13H20N2O3	253.15	165.05	252.15	$[M+H]^+$	Dihydrocaffeoylputrescine	Phenolamine
C29H39N3O8	558.28	207.07	557.27	$[M+H]^+$	N1,N8-Bis(sinapoyl)spermidine	Phenolamine
C6H14N2O	131.12	114.09	130.11	$[M+H]^+$	N-Acetylputrescine	Phenolamine
C14H20N2O3	265.15	177.06	264.15	$[M+H]^+$	N-Feruloylputrescine	Phenolamine
C13H18N2O2	235.14	147.04	234.14	$[M+H]^+$	p-Coumaroylputrescine	Phenolamine
C4H12N2	89.11	72.08	88.10	$[M+H]^+$	Putrescine	Phenolamine
C7H19N3	146.17	72.20	145.16	$[M+H]^+$	Spermidine	Phenolamine
C10H26N4	203.22	83.00	202.22	$[M+H]^+$	Spermine	Phenolamine
C5H9NO	100.08	56.05	99.07	$[M+H]^+$	2-Piperidone	Piperidine alkaloids
C10H8N2	157.00	130.00	156.07	$[M+H]^+$	3-Indoleacetonitrile	Plumerane
C11H11NO2	190.09	118.07	189.07	$[M+H]^+$	3-Indolepropionic acid	Plumerane
C8H7N	118.07	91.00	117.06	$[M+H]^+$	Indole	Plumerane
C9H7NO	146.06	91.00	145.05	$[M+H]^+$	Indole-3-carboxaldehyde	Plumerane
C9H7NO2	160.04	116.00	161.05	$[M-H]^-$	Indole-3-carboxylic acid	Plumerane
C11H11NO3	206.10	147.50	205.07	$[M+H]^+$	Methoxyindoleacetic acid	Plumerane
C10H12N2	161.11	144.00	160.10	$[M+H]^+$	Tryptamine	Plumerane
C15H22N2O	247.18	179.16	246.17	$[M+H]^+$	7,11-Dehydromatrine	Quinorisidine alkaloids

$C15H24N2O2$	265.19	247.18	264.18	$[M+H]^+$	9α-Hydroxymatrine	Quinorisidine alkaloids
$C11H14N2O$	191.12	148.00	190.11	$[M+H]^+$	Cytisine	Quinorisidine alkaloids
$C15H24N2O$	249.20	152.14	248.19	$[M+H]^+$	Isomatrine	Quinorisidine alkaloids
$C15H24N2O$	249.20	148.00	248.19	$[M+H]^+$	Matrine	Quinorisidine alkaloids
$C15H24N2O2$	265.19	247.18	264.18	$[M+H]^+$	Oxymatrine	Quinorisidine alkaloids
$C15H22N2O2$	263.18	245.17	262.17	$[M+H]^+$	Oxysophocarpine	Quinorisidine alkaloids
$C15H20N2O2$	261.16	243.15	260.15	$[M+H]^+$	Baptifoline	Sesquiterpene alkaloids

Supplementary Table 3. Differential metabolites of AmR and AcR sanples.

Formula	Compounds	VIP	Log2Fold_Change	Type
C5H11N	Piperidine	1.12	-1.01	down
C4H12N2	Putrescine	1.13	4.46	up
C6H6O	Phenol	1.13	-2.48	down
C6H14N2O	N-Acetylputrescine	1.12	4.68	up
C6H13NO2	6-Deoxyfagomine	1.12	-1.13	down
C8H9NO	N-benzylformamide	1.13	1.34	up
C8H9NO	2'-Aminoacetophenone	1.11	-1.67	down
C8H8O2	2-Methylbenzoic acid	1.12	-1.63	down
C7H7NO2	4-Aminobenzoic acid	1.05	1.15	up
C7H7NO2	Nicotinic Acid Methyl Ester(Methyl Nicotinate)	1.12	-1.83	down
C7H6O3	2,4-Dihydroxybenzaldehyde	1.13	-2.36	down
C5H9N3O2	2-Amino-4,5-dihydro-1H-imidazole-4-acetic acid	1.12	-2.13	down
C10H8O	2-Naphthol	1.11	1.33	up
C10H8O	1-Naphthol	1.07	1.69	up
C9H7NO	Indole-3-carboxaldehyde	1.13	-1.49	down
C7H19N3	Spermidine	1.12	3.12	up
C9H8O2	3,4-Dihydrocoumarin	1.11	2.00	up
C9H8O2	p-Coumaraldehyde	1.12	1.95	up
C9H10O2	p-Coumaryl alcohol	1.12	1.44	up
C9H10O2	4'-Hydroxypropiophenone	1.12	1.73	up
C8H8O3	2,5-dihydroxyphenylethanone	1.06	-1.19	down
C8H8O3	3-Methoxybenzoic acid	1.08	-1.26	down
C8H8O3	Anisic acid	1.13	-3.40	down
C8H8O3	2',4'-Dihydroxyacetophenone	1.09	-1.56	down
C7H7NO3	3-Hydroxyanthranilic acid	1.11	-2.94	down
C7H6O4	2,4-Dihydroxybenzoic acid	1.13	14.97	up
C8H10O3	Vanillyl alcohol	1.12	-8.22	down
C10H12N2	Tryptamine	1.11	-1.60	down
C9H7NO2	Indole-3-carboxylic acid	1.13	-1.56	down
C9H6O3	4-Hydroxycoumarin	1.12	-1.49	down
C9H8O3	4-hydroxyphenyl acrylaldehyde	1.05	1.21	up
C10H12O2	4-Phenylbutyric Acid	1.08	1.06	up
C8H7NO3	2-(Formylamino)benzoic acid	1.13	-2.07	down
C9H10O3	2,3-Dimethoxybenzaldehyde	1.04	-2.16	down
C9H10O3	Methyl anisate	1.09	-3.48	down
C9H10O3	Ethylsalicylate	1.13	-14.19	down
C8H9NO3	2-Amino-3-methoxybenzoic acid	1.12	-1.68	down
C8H8O4	3,4-Dihydroxybenzeneacetic acid	1.12	4.44	up

C8H8O4	4-Methoxysalicylic Acid	1.12	-2.13	down
C7H6O5	Gallic acid	1.07	-2.12	down
C8H15NO3	6-Acetamidohexanoic acid	1.13	-14.13	down
C10H8O3	7-Methoxycoumarin	1.13	-11.72	down
C10H8O3	6-Hydroxy-4-methylcoumarin	1.11	-1.66	down
C9H6O4	Daphnetin	1.08	-1.15	down
C9H6O4	Esculetin	1.13	-12.05	down
C9H8O4	Caffeic acid	1.12	-1.68	down
C13H8O	9-Fluorenone	1.11	1.70	up
C10H12O3	Propyl 4-hydroxybenzoate	1.11	1.51	up
C9H11NO3	N-(2-Hydroxy-4-methoxyphenyl)acetamide	1.13	1.51	up
C9H10O4	Vanillic acid methyl ester	1.11	-1.53	down
C9H10O4	2,6-Dimethoxybenzoic acid	1.11	-2.27	down
C9H10O4	3,4-Dimethoxybenzoic acid; Veratric acid	1.13	-11.18	down
$C5H15NO4P^{+}$	O-Phosphocholine	1.13	5.37	up
C11H9O2N	3-amino-2-naphthoic acid	1.11	-1.21	down
C11H11NO2	3-Indolepropionic acid	1.10	-1.35	down
C10H9NO3	2-Oxo-3,4-dihydro-1H-quinoline-3-carboxylic acid	1.11	-1.04	down
C10H10O4	Ferulic acid*	1.10	-1.16	down
C10H10O4	Methyl caffeate	1.06	3.38	up
C11H12N2O2	1-Methoxy-indole-3-acetamide	1.12	-1.33	down
C11H11NO3	Methoxyindoleacetic acid	1.12	-1.47	down
C11H12O4	Sinapinaldehyde	1.13	-3.78	down
C10H10O5	Dimetghyl 4-Hydroxyisophthalate	1.10	-2.61	down
C8H6Cl2O3	2,4-Dichlorophenoxyacetic Acid	1.12	4.11	up
C11H11NO4	Methyl dioxindole-3-acetate	1.13	-12.12	down
C10H8O6	Sideretin (5,7,8-Trihydroxy-6-methoxycoumarin)	1.07	-2.26	down
C11H12O5	Sinapic acid	1.10	-2.07	down
C14H12O3	Benzyl salicylate	1.07	1.28	up
C11H10O6	Benzoylmalic acid	1.12	-1.89	down
C12H14O5	Methyl sinapate	1.08	-2.11	down
C12H14O5	3,4,5-Trimethoxycinnamic acid	1.08	-1.41	down
C15H22N2O	7,11-Dehydromatrine	1.04	1.27	up
C15H22N2O	Sophocarpine	1.05	1.52	up
C15H22N2O	Isosophocarpine	1.04	1.17	up
C15H10O4	Daidzein	1.11	-1.41	down
C15H10O4	7,8-Dihydroxy-4-phenylcoumarin	1.06	-1.91	down
C15H12O4	Liquiritigenin	1.13	1.51	up
C12H16O6	Phenyl-beta-D-glucoside	1.12	7.13	up
C15H20N2O2	Baptifoline	1.12	-1.80	down

C16H12O4	Formononetin (7-Hydroxy-4'-methoxyisoflavone)	1.10	-1.32	down
C16H12O4	Isoformononetin	1.13	-13.64	down
C16H14O4	Echinatin	1.12	-1.62	down
C15H12O5	Pinobanksin*	1.13	1.82	up
C15H12O5	Naringenin (5,7,4'-Trihydroxyflavanone)*	1.12	1.73	up
C15H12O5	Butin	1.12	1.93	up
C16H16O4	(3R)-Vestitol	1.11	-2.59	down
C15H14O5	Epiafzelechin	1.10	-2.40	down
C11H22N2O4S	Pantetheine	1.12	-2.58	down
C16H10O5	pseudobaptigenin	1.12	-1.39	down
C17H14O4	7,4'-Di-O-methyldaidzein	1.13	6.05	up
C16H12O5	Biochanin A*	1.12	-1.70	down
C16H12O5	Prunetin (5,4'-Dihydroxy-7-methoxyisoflavone)*	1.12	-1.37	down
C16H12O5	3'-Methoxydaidzein	1.12	-1.74	down
C16H12O5	5-Hydroxy-3,7dimethoxy-1,4-phenanthrenequinone	1.12	-1.72	down
C16H12O5	Maackiain	1.12	-1.24	down
C16H12O5	1,3-dihydroxy-6-methoxy-7-methylanthraquinone	1.12	-2.21	down
C16H12O5	Genkwanin (Apigenin 7-methyl ether)	1.12	-1.94	down
C16H12O5	Acacetin	1.13	-1.76	down
C16H12O5	Emodin-1-methyl ether	1.13	-1.45	down
C17H16O4	Phenethyl caffeate	1.06	-1.15	down
C15H10O6	2'-Hydroxygenistein	1.11	-1.38	down
C15H10O6	Aureusidin	1.13	-1.59	down
C16H14O5	kushenin	1.12	-1.24	down
C13H18O7	Salicin	1.13	12.59	up
C17H18O4	6,7-cis-Dihydroxy-2-(2-phenylethyl)-5,6,7,8-tetrahydrochromone	1.12	1.31	up
C14H10O7	4-(3,4,5-Trihydroxybenzoxy)benzoic acid	1.12	3.24	up
C15H14O6	Catechin	1.12	2.89	up
C15H14O6	Epicatechin	1.12	3.03	up
C15H14O6	5,7,3',4',5'-Pentahydroxyflavan (Tricetiflavan)	1.12	3.18	up
C13H8O8	Brevifolin carboxylic acid	1.09	3.07	up
C17H12O5	7-O-methylpseudobaptigenin	1.13	6.36	up
C17H14O5	Afrormosin (6,4'-Dimethoxy-7-Hydroxyisoflavone)	1.13	-3.08	down
C17H14O5	Puerol A	1.12	-2.61	down

C17H14O5	Pterocarpine	1.13	-2.48	down
C16H12O6	Aracarpene 2	1.12	-1.25	down
C16H12O6	2-hydroxyemodin-1-methylether	1.13	-1.61	down
C16H12O6	Aracarpene 1	1.13	-1.29	down
C16H12O6	Chrysoeriol*	1.13	-1.36	down
C16H12O6	Pratensein	1.13	-1.82	down
C16H12O6	Kaempferide (3,5,7-Trihydroxy-4'-methoxyflavone)	1.13	-13.35	down
C16H12O6	Hispidulin (5,7,4'-Trihydroxy-6-methoxyflavone)*	1.13	-1.40	down
C16H12O6	Cajanin	1.13	-1.57	down
C13H16O8	4-O-Glucosyl-4-hydroxybenzoic acid	1.07	-1.77	down
C13H16O8	1-O-Salicyloyl-β-D-glucose	1.12	-1.42	down
C16H16O6	3'-O-Methyl-(-)-epicatechin	1.05	1.95	up
C15H14O7	Epigallocatechin	1.12	3.16	up
C13H12O9	2-Caffeoyl-L-tartaric acid (Caftaric acid)	1.08	-1.20	down
C18H16O5	6-Hydroxy-2-[2-(3'-methoxy-4'-hydroxyphenylethyl)]chromone	1.13	-11.98	down
C15H8O8	3-O-Methylellagic acid	1.07	-2.89	down
C16H12O7	3-O-Methylquercetin	1.12	-2.65	down
C16H12O7	Capillarisin	1.12	-2.20	down
C13H16O9	Protocatechuic acid-4-O-glucoside*	1.13	-3.00	down
C13H16O9	1-O-Gentisoyl-D-glucoside*	1.12	-3.36	down
C17H16O6	Cajanol	1.10	-1.41	down
C14H20O8	3,4-dihydroxyphenylethanol-β-D-glucoside	1.13	-13.27	down
C14H20O8	5-(2-Hydroxyethyl)-2-O-glucosylphenol	1.13	12.44	up
C14H20O8	Vanilloloside	1.13	-3.05	down
C16H16O7	Trans-5-O-(p-Coumaroyl)shikimate	1.10	-1.41	down
C14H10O9	Digallic acid	1.13	-9.97	down
C20H39NO2	N-Oleoylethanolamine	1.12	-2.05	down
C15H18O8	1-O-p-Coumaroyl-β-D-glucose	1.13	-3.76	down
C15H21NO7	N-benzoyl-2-aminoethyl-β-D-glucoside	1.13	-1.94	down
C16H10O8	3,3'-O-Dimethylellagic Acid	1.13	-5.92	down
C17H14O7	5,7-Dihydroxy-2'-methoxy-3',4'-methyleneoxydihydroisoflavone	1.13	-3.99	down
C14H18O9	1-O-Vanilloyl-D-Glucose	1.06	-1.03	down
C14H18O9	Vanillic acid-4-O-glucoside	1.11	2.77	up
C14H18O9	2,4,6-trihydroxy-acetophenone-6-O-glucoside	1.13	1.62	up
C15H22O8	3,4,5-Trimethoxyphenyl-1-O-Glucoside	1.13	1.51	up
C13H16O10	Gallic acid-4-O-glucoside	1.12	-1.97	down
C15H12O9	Galloyl Methyl gallate	1.11	12.16	up

$C_{20}H_{16}O_5$	Psoralidin	1.10	-2.11	down
$C_{20}H_{18}O_5$	Licoflavone C	1.08	1.33	up
$C_{20}H_{18}O_5$	Psoralenol	1.12	-1.94	down
$C_{15}H_{16}O_9$	Sinapoyl malate	1.03	-1.10	down
$C_{15}H_{16}O_9$	Esculin (6,7-DihydroxyCoumarin-6-glucoside)	1.03	-1.34	down
$C_{20}H_{20}O_5$	Kushenol S	1.12	-2.19	down
$C_{15}H_{18}O_9$	Vanillic Acid-4-O-Glucuronide	1.10	-2.97	down
$C_{15}H_{18}O_9$	1-O-Caffeoyl-β-D-glucose	1.13	-12.34	down
$C_{17}H_{12}O_8$	3,3',4-O-Trimethylellagic acid	1.11	-3.85	down
$C_{15}H_{20}O_9$	Dihydrocaffeoylglucose	1.12	-1.60	down
$C_{16}H_{18}O_9$	Cryptochlorogenic acid (4-O-Caffeoylquinic acid)	1.02	1.21	up
$C_{16}H_{20}O_9$	Ferulic acid-4-O-glucoside	1.12	-2.07	down
$C_{16}H_{20}O_9$	4-O-β-D-glucosylferulic acid	1.13	-6.19	down
$C_{16}H_{20}O_9$	1-O-Feruloyl-β-D-glucose	1.13	-6.42	down
$C_{20}H_{22}O_6$	Dehydrodiconiferyl alcohol	1.10	1.86	up
$C_{18}H_{16}O_8$	5,7,4'-trihydroxy-6,3',5'-trimethoxyisoflavone	1.12	-4.84	down
$C_{13}H_{14}O_{12}$	2-O-Galloylmucic acid	1.09	1.36	up
$C_{16}H_{16}O_{10}$	Scopoletin-7-O-glucuronide	1.13	-10.64	down
$C_{16}H_{20}O_{10}$	Trihydroxycinnamoylquinic acid	1.13	2.30	up
$C_{20}H_{20}O_7$	(8'R,7'S)-(-)-8-Hydroxy-α-conidendrin	1.12	-1.68	down
$C_{17}H_{22}O_{10}$	4-O-Glucosyl-sinapate	1.13	-11.31	down
$C_{17}H_{22}O_{10}$	1-O-Sinapoyl-D-glucose	1.13	-13.57	down
$C_{19}H_{30}O_8$	Roseoside	1.12	-4.02	down
$C_{25}H_{26}O_4$	Isolicoflavone B	1.05	1.10	up
$C_{22}H_{22}O_8$	1-O-Feruloyl-3-O-p-Coumaroylglycerol	1.13	-2.02	down
$C_{21}H_{20}O_9$	Daidzein-7-O-glucoside(Daidzin)	1.13	-4.63	down
$C_{19}H_{28}O_{10}$	Hydrangeifolin I	1.12	-3.26	down
$C_{20}H_{18}O_{10}$	Luteolin-8-C-arabinoside	1.13	-8.73	down
$C_{21}H_{22}O_9$	Isoliquiritin	1.13	-2.92	down
$C_{21}H_{22}O_9$	Liquiritigenin-4'-O-Glucoside (Liquiritin)	1.13	-3.02	down
$C_{21}H_{18}O_{10}$	Chrysin-7-O-Glucuronide	1.01	1.52	up
$C_{22}H_{22}O_9$	Formononetin-7-O-glycoside (Ononin)	1.13	-4.51	down
$C_{20}H_{16}O_{11}$	Caffeoyl(p-Hydroxybenzoyl)tartaric acid	1.12	1.90	up
$C_{21}H_{20}O_{10}$	Genistein-7-O-Glucoside (Genistin)	1.13	9.65	up
$C_{21}H_{20}O_{10}$	Apigenin-7-O-glucoside(Cosmosiin)	1.11	-3.39	down
$C_{21}H_{20}O_{10}$	Oroxin A	1.13	-14.57	down
$C_{21}H_{20}O_{10}$	Emodin-8-O-glucoside	1.13	-12.57	down
$C_{21}H_{20}O_{10}$	Genistein-8-C-glucoside	1.07	1.84	up
$C_{21}H_{20}O_{10}$	Apigenin-5-O-glucoside	1.13	12.66	up

$C_{18}H_{24}O_{12}$	Licoagroside B	1.12	1.75	up
$C_{21}H_{22}O_{10}$	Butin-7-O-glucoside*	1.06	-1.83	down
$C_{21}H_{22}O_{10}$	6-O-Caffeoylarbutin	1.12	-4.13	down
$C_{21}H_{22}O_{10}$	Naringenin-4'-O-glucoside*	1.09	-2.04	down
$C_{21}H_{24}O_{10}$	Phloretin-4'-O-glucoside (Trilobatin)	1.03	1.86	up
$C_{26}H_{30}O_{6}$	Kushenol Ca	1.13	-8.62	down
$C_{26}H_{30}O_{6}$	Leachianone A	1.13	-8.72	down
$C_{21}H_{18}O_{11}$	Rhein-8-O-glucoside	1.13	-14.07	down
$C_{22}H_{22}O_{10}$	Glycitin	1.12	-1.40	down
$C_{22}H_{22}O_{10}$	Calycosin-7-O-glucoside	1.09	-1.44	down
$C_{22}H_{22}O_{10}$	3'-Methoxydaidzin	1.12	-2.73	down
$C_{22}H_{22}O_{10}$	Biochanin A-7-O-glucoside (Sissotrin)	1.13	-2.86	down
$C_{22}H_{22}O_{10}$	Prunetin-5-O-glucoside	1.13	-2.77	down
$C_{22}H_{22}O_{10}$	Trifolirhizin (Maackiain-3-O-glucoside)	1.13	-6.83	down
$C_{21}H_{20}O_{11}$	Luteolin-7-O-glucoside (Cynaroside)	1.07	-1.53	down
$C_{21}H_{20}O_{11}$	Aureusidin-4-O-glucoside	1.13	-15.78	down
$C_{21}H_{20}O_{11}$	Luteolin-3'-O-glucoside	1.13	-15.93	down
$C_{21}H_{20}O_{11}$	Amoenin	1.12	-4.63	down
$C_{21}H_{20}O_{11}$	Kaempferol-3-O-glucoside (Astragalin)*	1.13	-15.84	down
$C_{21}H_{20}O_{11}$	Kaempferol-4'-O-glucoside	1.13	-15.91	down
$C_{21}H_{20}O_{11}$	Kaempferol-3-O-galactoside (Trifolin)	1.13	-3.97	down
$C_{21}H_{20}O_{11}$	HydroxyAloe-emodin-8-O-glucoside	1.13	-2.96	down
$C_{21}H_{20}O_{11}$	Kaempferol-7-O-glucoside*	1.08	-1.92	down
$C_{21}H_{22}O_{11}$	Eriodictyol-7-O-glucoside	1.13	-9.71	down
$C_{21}H_{22}O_{11}$	Aromadendrin-7-O-glucoside	1.08	1.19	up
$C_{30}H_{46}O_{3}$	Betulonic acid	1.01	-1.72	down
$C_{30}H_{46}O_{3}$	Urs-12(13)-en-3-one-28-oic acid	1.07	-1.79	down
$C_{30}H_{48}O_{3}$	24,30-Dihydroxy-12(13)-enolupinol	1.07	-1.07	down
$C_{23}H_{22}O_{10}$	6"-O-Acetyldaidzin	1.08	-3.56	down
$C_{23}H_{24}O_{10}$	6,4'-Dimethoxyisoflavone-7-O-glucoside (Wistin)	1.13	-6.12	down
$C_{22}H_{22}O_{11}$	Chrysoeriol-7-O-glucoside*	1.01	-1.47	down
$C_{22}H_{22}O_{11}$	Diosmetin-7-O-galactoside*	1.13	-6.76	down
$C_{22}H_{22}O_{11}$	Diosmetin-7-O-glucoside*	1.13	-6.47	down
$C_{22}H_{22}O_{11}$	Chrysoeriol-8-C-glucoside (Scoparin)	1.11	2.97	up
$C_{22}H_{22}O_{11}$	Hispidulin-7-O-Glucoside*	1.13	-3.45	down
$C_{20}H_{30}O_{12}$	Verbasoside	1.13	9.19	up
$C_{21}H_{20}O_{12}$	Isoquercitrin	1.13	12.82	up
$C_{21}H_{20}O_{12}$	Quercetin-7-O-glucoside*	1.13	-12.69	down
$C_{22}H_{24}O_{11}$	Hesperetin-5-O-glucoside	1.11	-2.70	down
$C_{30}H_{48}O_{4}$	2α-Hydroxyursolic acid	1.09	-1.24	down
$C_{30}H_{48}O_{4}$	Maslinic acid*	1.11	-1.20	down
$C_{30}H_{48}O_{4}$	23-Hydroxybetulinic acid	1.13	-1.58	down

C30H48O4	2-Hydroxyoleanolic acid	1.11	-1.28	down
C30H50O4	Soyasapogenol A	1.07	-1.71	down
C22H22O12	Isorhamnetin-7-O-glucoside (Brassicin)	1.10	-2.80	down
C22H22O12	Nepetin-7-O-glucoside	1.12	-2.94	down
C23H20O12	Rhein-8-O-(6'-O-acetyl)glucoside	1.11	-2.35	down
C24H24O11	6''-O-Acetylglycitin	1.12	-2.13	down
C30H48O5	Madasiatic acid	1.11	-1.33	down
C30H48O5	2α,3α,23-trihydroxyolean-12-en-28-oic acid	1.11	-1.49	down
C23H22O12	Kaempferol-3-O-(6''-acetyl)glucoside	1.13	14.41	up
C23H24O12	Tricin-7-O-Glucoside	1.12	-2.39	down
C24H22O12	6''-O-Malonyldaidzin	1.13	-4.26	down
C23H20O13	Kaempferol-3-O-(2''-O-acetyl)glucuronide	1.11	-3.24	down
C28H24O9	Daidzein-7-O-(2''-benzoyl)rhamnoside	1.13	-3.60	down
C21H28O14	1-O-Caffeoyl-(6-O-glucosyl)-β-D-glucose	1.13	-10.52	down
C30H48O6	2α,3α,19α,23-tetrahydroxy-12-ursen-28-oic acid	1.11	-2.13	down
C23H24O13	5,6,3',4'-Tetrahydroxy-3,7-dimethoxyflavone-6-O-glucoside	1.13	-12.34	down
C25H24O12	Formononetin-7-O-(6''-Malonyl)glucoside	1.13	-3.90	down
C24H22O13	Genistein-7-O-(6''-malonyl)glucoside	1.13	-6.29	down
C24H22O13	Apigenin-7-O-(6''-malonyl)glucoside	1.13	-9.70	down
C24H22O13	6''-O-Malonylgenistin	1.13	-3.35	down
C26H32O11	Pinoresinol-4-O-glucoside	1.12	-2.06	down
C26H32O11	Dehydrodiconiferyl alcohol-gamma'-O-glucoside	1.13	-9.97	down
C23H22O14	Tricin-7-O-saccharic acid	1.09	-1.43	down
C24H26O13	Rosmarinic acid-3'-O-glucoside	1.04	-2.67	down
C26H34O11	Lariciresinol-4'-O-glucoside	1.09	-3.37	down
C26H36O11	Secoisolariciresinol 4-O-glucoside	1.05	1.12	up
C25H24O13	6''-O-Malonylglycitin	1.13	-1.56	down
C24H22O14	Kaempferol-3-O-(6''-malonyl)galactoside*	1.12	-2.04	down
C24H22O14	Kaempferol-3-O-(6''-malonyl)glucoside*	1.13	-17.37	down
C26H32O12	1-Hydroxypinoresinol-4'-O-Glucoside	1.13	-11.22	down
C25H24O14	Diosmetin-7-O-(6''-malonyl)glucoside*	1.11	-2.74	down
C25H24O14	Chrysoeriol-7-O-(6''-malonyl)glucoside*	1.13	-2.24	down
C25H24O14	Kaempferide-3-O-(6''-malonyl)glucoside	1.13	-4.55	down

C26H28O13	Daidzein-7-O-apiosyl(1→6)glucoside	1.07	1.26	up
C24H22O15	Quercetin-7-O-(6"-malonyl)glucoside	1.13	-14.04	down
C29H39N3O8	N1,N8-Bis(sinapoyl)spermidine	1.13	-9.92	down
C30H26O11	Epicatechin-epiafzelechin	1.06	1.41	up
C27H30O13	Kushenol O	1.13	10.82	up
C27H30O13	Glycyroside	1.10	-1.07	down
C28H34O12	Pinoresinol-4-O-(6"-acetyl)glucoside	1.13	-3.09	down
C25H24O15	Isorhamnetin-3-O-(6"-malonylglucoside)	1.13	-2.74	down
C26H28O14	Isovitexin-2"xyloside	1.13	12.20	up
C26H28O14	Apigenin-7-O-(2"-glucosyl)arabinoside	1.13	-8.52	down
C26H28O14	Apigenin-6-C-(2"-xylosyl)glucoside	1.13	11.93	up
C30H24O12	Procyanidin A1	1.12	3.66	up
C30H24O12	Procyanidin A2	1.12	3.88	up
C30H26O12	Procyanidin B2	1.11	3.22	up
C30H26O12	Procyanidin B4	1.12	3.74	up
C30H26O12	Procyanidin B3*	1.11	2.79	up
C30H26O12	Apigenin-7-O-(6"-p-Coumaryl)glucoside	1.13	-13.26	down
C27H30O14	Soyamaloside B	1.13	-4.35	down
C26H28O15	Kaempferol-3-O-sambubioside	1.04	1.45	up
C27H32O14	Liquiritigenin-7,4'-O-diglucoside	1.12	-4.29	down
C27H32O14	Naringenin-7-O-Rutinoside(Narirutin)	1.13	-11.41	down
C31H28O12	Procyanidin A6	1.12	8.83	up
C31H28O12	8,8'-Methylenebiscatechin	1.12	9.56	up
C27H30O15	Luteolin-7-O-rutinoside*	1.13	-11.28	down
C27H30O15	Kaempferol-3-O-glucoside-7-O-rhamnoside	1.09	-2.50	down
C27H30O15	Kaempferol-3-O-robinobioside(Biorobin)	1.13	-16.60	down
C27H30O15	Kaempferol-3-O-rutinoside(Nicotiflorin)*	1.11	-3.66	down
C27H30O15	Vitexin-2"-O-glucoside	1.13	-2.31	down
C27H30O15	"Vitexin-2""-O-galactoside"	1.13	-12.82	down
C27H30O15	Kaempferol-3-O-neohesperidoside*	1.07	-2.83	down
C27H32O15	Eriodictyol-7-O-Rutinoside (Eriocitrin)	1.13	-12.92	down
C28H32O15	Diosmetin-7-O-rutinoside (Diosmin)*	1.12	-5.36	down
C28H32O15	Diosmetin-7-O-Neohesperidoside (Neodiosmin)*	1.13	-11.79	down
C31H28O13	Hispidulin-7-O-(6"-O-p-Coumaroyl)Glucoside	1.13	-10.97	down
C27H30O16	Luteolin-7,3'-di-O-glucoside	1.02	-1.61	down
C27H30O16	Luteolin-7-O-gentiobioside	1.11	-2.59	down
C27H30O16	Orientin-2"-O-galactoside	1.13	12.78	up

C27H30O16	Quercetin-3-O-robinobioside	1.13	-9.38	down
C27H30O17	Quercetin-3-O-sophoroside (Baimaside)	1.09	2.18	up
C36H58O9	Soyasapogenol B-3-O-glucuronide	1.13	16.02	up
C28H32O17	Isorhamnetin-3-O-sophoroside	1.13	-11.33	down
C30H32O18	Luteolin-7-O-(6''-malonyl)glucoside-5-O-rhamnoside	1.06	-2.49	down
C36H36O17	Isovitexin-2''-O-(6'''-p-coumaroyl)glucoside	1.13	-10.82	down
C33H40O20	Kaempferol-3-O-sophoroside-7-O-rhamnoside	1.04	-2.18	down
C33H40O20	Kaempferol-3-O-neohesperidoside-7-O-glucoside	1.12	-10.30	down
C41H66O13	Soyasaponin IV	1.12	-1.40	down
C34H42O21	Isorhamnetin-3-O-rutinoside-4'-O-glucoside	1.13	-10.14	down
C34H42O21	Isorhamnetin-3-O-sophoroside-7-O-rhamnoside	1.13	-10.99	down
C42H68O14	Soyasaponin βb'	1.11	-1.37	down
C42H68O14	Soyasaponin III	1.11	-1.96	down
C42H64O16	Ceanothic acid-3-O-glucuronic acid-glucose	1.12	-3.98	down
C42H66O16	Bayogenin-3-O-glucuronide-28-O-glucoside	1.13	-1.47	down
C42H62O18	Dihydroxyglycyrrhetinic acid	1.13	-9.92	down
C45H38O18	Catechin-catechin-catechin	1.09	3.20	up
C48H74O17	Soyasaponin γg	1.12	-2.50	down
C48H76O18	Soyasapogenol E-3-O-rhamnosyl(1,2)glucosyl(1,2)glucuronide	1.13	-2.06	down
C48H76O18	Soyasaponin βe	1.11	-1.90	down
C48H78O18	Abrisaponin A	1.12	-1.09	down
C48H78O18	Soyasaponin βb (Soyasaponin I)	1.11	-1.12	down
C47H72O20	Medicagenic acid-3-O-glucuronide-28-O-rhamnosyl(1,2)-arabinoside	1.13	-1.33	down
C48H76O19	Soyasaponin βd	1.13	-13.57	down
C48H78O19	Soyasaponin βa (Soyasaponin V)	1.13	15.88	up
C48H74O20	Abrisaponin I	1.12	-2.66	down
C48H74O20	Ceanothic acid-3-O-glucuronic acid-rutinose	1.13	-3.83	down
C48H76O20	Hederagenin-3-O-glucuronide-28-O-glucosyl(1,2)glucoside	1.13	-14.70	down
C48H78O20	Abrisaponin L	1.10	-1.93	down
C54H84O21	Soyasaponin βg (Soyasaponin VI)	1.07	-2.52	down

Supplementary Table 4. Differential metabolites of AmL and AcL sanples.

Formula	Compounds	VIP	Log2Fold_Change	Type
$C_3H_6N_2$	β-Aminopropionitrile	1.01	(1.31)	down
$C_5H_{14}N_2$	Cadaverine	1.14	3.12	up
C_8H_7N	Indole	1.07	(1.62)	down
C_7H_7NO	Benzamide	1.14	2.83	up
C_9H_7N	Isoquinoline	1.11	(1.54)	down
$C_6H_{14}N_2O$	N-Acetylputrescine	1.14	2.74	up
C_8H_9NO	N-benzylformamide	1.08	(1.02)	down
$C_7H_7NO_2$	4-Aminobenzoic acid	1.10	1.29	up
$C_7H_6O_3$	Salicylic acid	1.13	(1.68)	down
$C_8H_{10}O_2$	Tyrosol	1.13	(1.77)	down
$C_7H_{19}N_3$	Spermidine	1.07	1.60	up
$C_9H_8O_2$	Cinnamic acid	1.14	1.71	up
$C_8H_6O_3$	Benzoylformic acid	1.14	1.39	up
$C_8H_8O_3$	2-Hydroxy-4-Methoxybenzaldehyde	1.12	1.29	up
$C_7H_7NO_3$	3-Aminosalicylic acid	1.13	1.20	up
$C_7H_6O_4$	2,5-Dihydroxybenzoic acid; Gentisic Acid*	1.14	(1.06)	down
$C_7H_6O_4$	3,4-Dihydroxybenzoic acid (Protocatechuic acid)*	1.14	(1.06)	down
$C_7H_6O_4$	2,4-Dihydroxybenzoic acid	1.14	16.43	up
$C_{10}H_{12}N_2$	Tryptamine	1.11	1.94	up
$C_6H_{11}NO_4$	DL-2-Aminoadipic acid	1.14	1.82	up
$C_9H_6O_3$	Umbelliferone	1.08	1.26	up
$C_9H_{10}O_3$	2-Hydroxy-3-phenylpropanoic acid	1.13	1.47	up
$C_9H_{10}O_3$	Methyl anisate	1.14	(8.17)	down
$C_9H_{10}O_3$	Ethylsalicylate	1.14	(12.67)	down
$C_8H_9NO_3$	2-Amino-3-methoxybenzoic acid	1.14	10.05	up
$C_8H_8O_4$	3,4-Dihydroxybenzeneacetic acid	1.14	16.34	up
$C_8H_8O_4$	Homogentisic acid	1.14	(16.94)	down
$C_8H_8O_4$	2',4',6'-Trihydroxyacetophenone	1.06	(1.35)	down
$C_8H_8O_4$	2,6-Dimethoxy-1,4-benzoquinone	1.14	(1.40)	down
$C_8H_8O_4$	3-Hydroxymandelate	1.05	(1.09)	down
$C_7H_6O_5$	2,4,6-Trihydroxybenzoic acid	1.03	2.78	up
$C_7H_6O_5$	Gallic acid	1.02	2.73	up
$C_8H_{15}NO_3$	6-Acetamidohexanoic acid	1.14	(11.56)	down
$C_7H_{14}N_2O_3$	N-α-Acetyl-L-ornithine	1.13	(1.38)	down
$C_9H_6O_4$	5,7-Dihydroxychromone	1.11	(1.31)	down
$C_9H_6O_4$	Daphnetin	1.12	10.14	up
$C_9H_6O_4$	Esculetin	1.13	(1.23)	down
$C_9H_8O_4$	Caffeic acid	1.14	(2.28)	down

C10H12O3	4-Methoxyphenylpropionic acid	1.10	(1.84)	down
C9H10O4	Syringaldehyde; 4-Hydroxy-3,5-Dimethoxybenzaldehyde	1.10	1.06	up
C9H10O4	Vanillic acid methyl ester	1.14	2.42	up
C9H10O4	3,4-Dimethoxybenzoic acid; Veratric acid	1.14	(12.60)	down
C8H8O5	Methyl gallate	1.09	4.33	up
C8H8O5	3-O-Methylgallic acid	1.07	2.64	up
C11H11NO2	3-Indolepropionic acid	1.06	(1.05)	down
C11H14N2O	Cytisine	1.08	(1.79)	down
C10H8O4	Scopoletin (7-Hydroxy-5-methoxycoumarin)	1.14	15.34	up
C10H8O4	Isoscopoletin (6-Hydroxy-7-Methoxycoumarin)	1.13	1.94	up
C10H10O4	Isoferulic Acid*	1.14	(3.28)	down
C10H10O4	Ferulic acid*	1.14	(3.50)	down
C10H10O4	Methyl caffeate	1.13	3.37	up
C10H12O4	Dihydroferulic Acid	1.12	(3.14)	down
C9H10O5	Syringic acid	1.12	(1.20)	down
C11H21NO2	5-(2-Hydroxypropyl)-hygrine	1.14	(1.03)	down
C11H12O4	Sinapinaldehyde	1.12	(3.03)	down
C10H12O5	Methyl Syringate	1.09	2.04	up
C12H14N2O2	Abrine	1.13	(2.07)	down
C11H11NO4	Methyl dioxindole-3-acetate	1.14	(12.18)	down
C12H14O4	Ethyl ferulate	1.14	(9.99)	down
C10H8O6	Sideretin (5,7,8-Trihydroxy-6-methoxycoumarin)	1.14	(2.67)	down
C11H12O5	Sinapic acid	1.14	(2.75)	down
C11H14O5	3,4'-Dihydroxy-3',5'-dimethoxypropiophe none	1.13	(1.14)	down
C11H14O5	Genipin	1.11	1.70	up
C11H10O6	Benzoylmalic acid	1.14	1.73	up
C15H20N2O	Sophoramine	1.13	1.54	up
C15H24N2O	Sophoridine	1.14	(3.38)	down
C13H20N2O3	Dihydrocaffeoylputrescine	1.03	(1.48)	down
C15H10O4	Daidzein	1.14	(2.39)	down
C12H16O6	Phenyl-beta-D-glucoside	1.11	2.79	up
C15H22N2O2	9α-Hydroxysophocarpine	1.02	(1.14)	down
C15H22N2O2	5α-Hydroxysophocarpine	1.00	(1.28)	down
C15H22N2O2	Oxysophocarpine	1.00	(1.17)	down
C15H24N2O2	Sophoridine N-oxide	1.09	(2.06)	down
C15H24N2O2	9α-Hydroxymatrine	1.08	(1.99)	down
C15H24N2O2	Oxymatrine	1.10	(1.92)	down
C15H10O5	Genistein	1.14	(11.72)	down
C15H10O5	3',4',7-Trihydroxyflavone	1.14	11.78	up

C15H10O5	Apigenin	1.14	(4.01)	down
C16H14O4	Echinatin	1.13	(2.28)	down
C15H12O5	Pinobanksin*	1.14	(2.69)	down
C15H12O5	Naringenin (5,7,4'-Trihydroxyflavanone)*	1.14	(2.72)	down
C15H12O5	Butin	1.13	(2.79)	down
C15H14O5	Epiafzelechin	1.14	(12.01)	down
C11H22N2O4S	Pantetheine	1.09	(1.17)	down
C15H24N2O3	(+)-5α,9α-Dihydroxymatrine	1.13	(2.53)	down
C17H14O4	7,4'-Di-O-methyldaidzein	1.12	(2.64)	down
C17H17NO3	P-Hydroxycinnamic acid p-hydroxyphenethylamine	1.14	(15.69)	down
C16H12O5	Biochanin A*	1.14	(1.71)	down
C16H12O5	Prunetin (5,4'-Dihydroxy-7-methoxyisoflavone)*	1.14	(2.12)	down
C16H12O5	3'-Methoxydaidzein	1.13	(3.16)	down
C16H12O5	Maackiain	1.14	2.54	up
C16H12O5	1,3-dihydroxy-6-methoxy-7-methylanthraquinone	1.14	2.31	up
C16H12O5	Genkwanin (Apigenin 7-methyl ether)	1.14	2.36	up
C16H12O5	Acacetin	1.14	(1.78)	down
C16H12O5	Emodin-1-methyl ether	1.10	1.48	up
C17H16O4	Phenethyl caffeate	1.14	(9.26)	down
C15H10O6	6-Demethoxycapillarisin	1.14	10.62	up
C15H10O6	Aureusidin	1.13	(1.31)	down
C15H10O6	Luteolin (5,7,3',4'-Tetrahydroxyflavone)	1.14	(1.11)	down
C15H10O6	Isoluteolin (Orobol)(5,7,3',4'-tetrahydroxyisoflavone)	1.14	(1.65)	down
C16H14O5	kushenin	1.12	(1.31)	down
C13H18O7	Salicin	1.14	14.97	up
C15H12O6	2-Hydroxy-2,3-dihydrogenistein	1.12	(2.34)	down
C15H12O6	2,6,7,4'-Tetrahydroxyisoflavanone	1.14	(12.12)	down
C14H10O7	4-(3,4,5-Trihydroxybenzoxy)benzoic acid	1.14	(4.13)	down
C15H14O6	Catechin	1.14	(4.60)	down
C15H14O6	Epicatechin	1.14	(4.64)	down
C15H14O6	5,7,3',4',5'-Pentahydroxyflavan (Tricetiflavan)	1.14	(4.24)	down
C13H8O8	Brevifolin carboxylic acid	1.14	(13.78)	down
C14H20N4O3	Caffeoylagmatine	1.14	20.02	up
C17H12O5	7-O-methylpseudobaptigenin	1.14	(3.18)	down
C17H14O5	Afrormosin (6,4'-Dimethoxy-7-Hydroxyisoflavone)	1.14	(3.51)	down

C17H14O5	Puerol A	1.14	(3.62)	down
C17H14O5	Pterocarpine	1.14	(2.89)	down
C16H12O6	Aracarpene 2	1.14	(3.52)	down
C16H12O6	Aracarpene 1	1.14	(3.54)	down
C16H12O6	Pratensein	1.14	(3.78)	down
C16H12O6	Kaempferide (3,5,7-Trihydroxy-4'-methoxyflavone)	1.14	11.98	up
C13H18O8	4-O-Glucosyl-3,4-dihydroxybenzyl alcohol	1.14	(2.62)	down
C16H16O6	3'-O-Methyl-(-)-epicatechin	1.14	(3.58)	down
C10H16O7N4	Vicine	1.14	2.30	up
C15H14O7	Epigallocatechin	1.14	(14.43)	down
C13H12O9	2-Caffeoyl-L-tartaric acid (Caftaric acid)	1.12	(2.82)	down
C14H16O8	1-O-Caffeoyl xylose	1.12	(1.26)	down
C14H18O8	6-O-Acetylarbutin	1.14	1.09	up
C16H12O7	Capillarisin	1.14	1.65	up
C13H16O9	Protocatechuic acid-4-O-glucoside*	1.14	(4.53)	down
C13H16O9	1-O-Gentisoyl-D-glucoside*	1.14	(3.30)	down
C17H16O6	Cajanol	1.10	1.05	up
C14H20O8	3,4-dihydroxyphenylethanol-β-D-glucoside	1.12	(3.76)	down
C14H20O8	5-(2-Hydroxyethyl)-2-O-glucosylphenol	1.14	(2.73)	down
C14H20O8	Vanilloloside	1.14	(4.61)	down
C14H10O9	Digallic acid	1.07	3.56	up
C15H18O8	Phenylpropionic acid-O-β-D-glucoside	1.14	2.03	up
C15H18O8	p-Coumaric acid-4-O-glucoside	1.14	2.30	up
C18H17NO5	N-[3-(4-Hydroxyphenyl)acryloyl]-L-tyrosine	1.14	(8.91)	down
C15H20O8	Demethyl coniferin	1.10	(2.13)	down
C16H24O7	3-Hydroxy-4-isopropylbenzylalcohol-3-O-glucoside	1.11	(2.64)	down
C16H10O8	3,3'-O-Dimethylellagic Acid	1.13	(1.06)	down
C14H18O9	Vanillic acid-4-O-glucoside	1.14	2.13	up
C13H16O10	Gallic acid-4-O-glucoside	1.14	(5.86)	down
C13H16O10	6-O-Galloyl-D-glucose	1.10	4.65	up
C19H12O6	Dicumarol	1.12	2.49	up
C20H16O5	Glabrone	1.02	(1.70)	down
C16H18O8	5-O-p-Coumaroylquinic acid*	1.14	(3.04)	down
C16H18O8	3-O-p-Coumaroylquinic acid*	1.14	(4.28)	down
C20H18O5	Licoflavone C	1.02	(2.38)	down
C15H16O9	Sinapoyl malate	1.10	(1.05)	down
C15H16O9	Esculin (6,7-DihydroxyCoumarin-6-glucoside)	1.11	(1.16)	down
C15H18O9	Vanillic Acid-4-O-Glucuronide	1.13	(1.34)	down

C15H18O9	1-O-Caffeoyl-β-D-glucose	1.13	(1.20)	down
C15H18O9	6-O-Caffeoyl-D-glucose	1.13	(1.41)	down
C16H22O8	Coniferin	1.14	(1.00)	down
C17H12O8	3,3',4-O-Trimethylellagic acid	1.14	(10.31)	down
C15H20O9	Syringaldehyde-4-O-glucoside	1.14	(12.30)	down
C14H18O10	1-O-(3,4-Dihydroxy-5-methoxy-benzoyl) -glucoside	1.14	(11.08)	down
C16H18O9	Chlorogenic acid (3-O-Caffeoylquinic acid)*	1.14	(3.71)	down
C16H18O9	Neochlorogenic acid (5-O-Caffeoylquinic acid)*	1.14	12.37	up
C16H18O9	Scopoletin-7-O-glucoside (Scopolin)	1.09	1.14	up
C16H20O9	Ferulic acid-4-O-glucoside	1.13	(2.07)	down
C16H20O9	4-O-β-D-glucosylferulic acid	1.14	(1.38)	down
C16H20O9	1-O-Feruloyl-β-D-glucose	1.13	(1.30)	down
C16H16O10	Scopoletin-7-O-glucuronide	1.14	(13.91)	down
C17H20O9	1-O-Feruloylquinic acid	1.14	(3.86)	down
C17H20O9	Chlorogenic acid methyl ester	1.14	(3.79)	down
C16H22O10	Swertiamarin	1.09	(1.18)	down
C25H28O4	Glabrol	1.11	(1.71)	down
C21H20O9	Daidzein-7-O-glucoside(Daidzin)	1.14	(13.58)	down
C22H22O9	Formononetin-7-O-glycoside (Ononin)	1.09	(1.68)	down
C20H16O11	Caffeoyl(p-Hydroxybenzoyl)tartaric acid	1.14	(13.26)	down
C21H20O10	Genistein-7-O-Glucoside (Genistin)	1.07	(1.45)	down
C21H20O10	Apigenin-7-O-glucoside(Cosmosiin)	1.13	(2.38)	down
C21H20O10	Apigenin-7-O-α-D-glucoside	1.14	(3.34)	down
C21H20O10	Sophoricoside	1.14	(3.38)	down
C21H20O10	Genistein-8-C-glucoside	1.14	(1.92)	down
C18H24O12	Licoagroside B	1.14	(4.03)	down
C21H22O10	Naringenin-7-O-glucoside (Prunin)	1.14	(2.05)	down
C21H24O10	Phloretin-2'-O-glucoside (Phlorizin)	1.11	(1.94)	down
C26H30O6	Kushenol D	1.13	1.78	up
C26H30O6	Kushenol Ca	1.10	1.94	up
C21H18O11	Rhein-8-O-glucoside	1.14	(11.72)	down
C22H22O10	Glycitin	1.13	(1.82)	down
C22H22O10	Calycosin-7-O-glucoside	1.14	(1.88)	down
C22H22O10	3'-Methoxydaidzin	1.08	(1.62)	down
C22H22O10	Biochanin A-7-O-glucoside (Sissotrin)	1.11	(1.69)	down
C22H22O10	Prunetin-5-O-glucoside	1.10	(1.59)	down
C22H22O10	Trifolirhizin (Maackiain-3-O-glucoside)	1.13	(3.53)	down
C21H20O11	Luteolin-7-O-glucoside (Cynaroside)	1.14	(3.62)	down
C21H20O11	Aureusidin-4-O-glucoside	1.14	(1.92)	down
C21H20O11	Luteolin-3'-O-glucoside	1.14	(1.79)	down
C21H20O11	Amoenin	1.12	1.15	up

C21H20O11	Kaempferol-3-O-glucoside (Astragalin)*	1.14	(1.90)	down
C21H20O11	Kaempferol-4'-O-glucoside	1.14	(1.85)	down
C21H20O11	HydroxyAloe-emodin-8-O-glucoside	1.13	(2.64)	down
C21H20O11	Kaempferol-7-O-glucoside*	1.13	(1.69)	down
C21H22O11	3',5,5',7-Tetrahydroxyflavanone-7-O-glucoside	1.14	(3.15)	down
C21H22O11	Eriodictyol-7-O-glucoside	1.14	(3.19)	down
C21H22O11	Aromadendrin-7-O-glucoside	1.11	1.63	up
C24H20O9	Catechin-(7,8-bc)-4β-(3,4-dihydroxyphenyl)-dihydro-2-(3H)-one	1.14	(14.29)	down
C21H24O11	Epicatechin glucoside	1.13	(5.69)	down
C27H32O6	Kushenol Da	1.14	11.19	up
C23H22O10	6''-O-Acetyldaidzin	1.14	9.41	up
C30H50O3	12,13-Dihydroursolic acid	1.08	1.10	up
C23H24O10	6,4'-Dimethoxyisoflavone-7-O-glucoside (Wistin)	1.13	(5.47)	down
C22H22O11	Chrysoeriol-8-C-glucoside (Scoparin)	1.14	2.23	up
C20H30O12	Verbasoside	1.14	(10.69)	down
C21H20O12	Quercetin-3-O-glucoside (Isoquercitrin)*	1.14	(4.47)	down
C22H24O11	Hesperetin-5-O-glucoside	1.14	(4.85)	down
C30H48O4	2α-Hydroxyursolic acid	1.14	13.30	up
C30H48O4	Maslinic acid*	1.14	13.17	up
C30H48O4	Corosolic acid*	1.14	(11.88)	down
C30H48O4	2,3-dihydroxy-12-ursen-28-oic acid	1.14	(12.01)	down
C23H22O11	6''-O-Acetylgenistin	1.12	(2.52)	down
C23H22O11	Apigenin-7-O-(6''-acetyl)glucoside	1.12	(2.43)	down
C30H50O4	Soyasapogenol A	1.14	2.21	up
C22H22O12	Isorhamnetin-7-O-glucoside (Brassicin)	1.14	3.23	up
C22H22O12	Nepetin-7-O-glucoside	1.14	3.24	up
C21H26O13	Scopoletin-7-O-xylosyl(1→6)glucoside	1.14	10.97	up
C30H48O5	Madasiatic acid	1.12	(1.64)	down
C30H48O5	2α,3α,23-trihydroxyolean-12-en-28-oic acid	1.10	(1.76)	down
C23H22O12	Kaempferol-3-O-(2''-acetyl)glucoside	1.05	(1.29)	down
C30H50O5	Cycloastragenol	1.14	11.27	up
C23H24O12	Tricin-7-O-Glucoside	1.14	(3.33)	down
C25H32O10	Isolariciresinol-9-O-xyloside	1.13	(3.13)	down
C24H22O12	6''-O-Malonyldaidzin	1.11	(3.51)	down
C23H20O13	Kaempferol-3-O-(2''-O-acetyl)glucuronide	1.14	(1.13)	down
C21H28O14	1-O-Caffeoyl-(6-O-glucosyl)-β-D-glucose	1.14	(2.21)	down
C30H48O6	2α,3α,19α,23-tetrahydroxy-12-ursen-28-oic acid	1.13	(2.38)	down

C23H24O13	5,6,3',4'-Tetrahydroxy-3,7-dimethoxyflavone-6-O-glucoside	1.14	(4.27)	down
C25H24O12	Formononetin-7-O-(6''-Malonyl)glucoside	1.14	(2.33)	down
C24H22O13	Apigenin-7-O-(6''-malonyl)glucoside	1.14	(3.12)	down
C24H22O13	6''-O-Malonylgenistin	1.14	13.12	up
C24H24O13	Naringenin-7-O-(6''-malonyl)glucoside	1.10	(2.30)	down
C26H32O11	Pinoresinol-4-O-glucoside	1.10	(1.30)	down
C26H32O11	Dehydrodiconiferyl alcohol-gamma'-O-glucoside	1.12	(3.36)	down
C23H22O14	Tricin-7-O-saccharic acid	1.13	(2.92)	down
C24H26O13	Rosmarinic acid-3'-O-glucoside	1.01	(1.27)	down
C26H34O11	Isolariciresinol-9'-O-glucoside	1.13	(2.33)	down
C26H34O11	Dihydrodehydrodiconiferyl alcohol-4-O-glucoside	1.14	(13.37)	down
C25H24O13	6''-O-Malonylglycitin	1.13	2.50	up
C24H22O14	Kaempferol-3-O-(6''-malonyl)galactoside*	1.12	(2.51)	down
C24H22O14	Kaempferol-3-O-(6''-malonyl)glucoside*	1.14	(2.00)	down
C24H22O14	Luteolin-7-O-(6''-malonyl)glucoside	1.14	(2.56)	down
C26H32O12	1-Hydroxypinoresinol-4'-O-Glucoside	1.12	(3.22)	down
C26H32O12	1-Hydroxypinoresinol-1-O-Glucoside	1.14	(12.69)	down
C25H24O14	Kaempferide-3-O-(6''-malonyl)glucoside	1.11	(1.63)	down
C24H22O15	Quercetin-7-O-(6''-malonyl)glucoside	1.14	(16.48)	down
C29H39N3O8	N1,N8-Bis(sinapoyl)spermidine	1.12	(1.66)	down
C30H26O11	Epicatechin-epiafzelechin	1.14	(13.53)	down
C27H30O13	Kushenol O	1.14	13.97	up
C27H30O13	Glycyroside	1.14	(3.30)	down
C28H34O12	Pinoresinol-4-O-(6''-acetyl)glucoside	1.11	(1.56)	down
C26H28O14	Kaempferol-3-O-arabinoside-7-O-rhamnoside	1.14	1.47	up
C26H28O14	Isovitexin-2''xyloside	1.14	18.74	up
C26H28O14	Apigenin-6-C-(2''-xylosyl)glucoside	1.14	18.68	up
C30H24O12	Procyanidin A1	1.14	(4.34)	down
C30H24O12	Procyanidin A2	1.14	(4.40)	down
C30H26O12	Procyanidin B2	1.14	(5.45)	down
C30H26O12	Procyanidin B4	1.14	(4.97)	down
C30H26O12	Procyanidin B1*	1.14	(13.55)	down
C30H26O12	Procyanidin B3*	1.14	(5.28)	down
C30H26O12	Apigenin-7-O-(6''-p-Coumaryl)glucoside	1.14	(2.59)	down
C27H30O14	Apigenin-7-O-rutinoside (Isorhoifolin)	1.14	(2.65)	down
C27H32O14	Liquiritigenin-7,4'-O-diglucoside	1.14	12.88	up
C27H32O14	Naringenin-7-O-Rutinoside(Narirutin)	1.14	(3.18)	down
C31H28O12	Procyanidin A6	1.14	(12.61)	down

C31H28O12	8,8'-Methylenebiscatechin	1.14	(12.58)	down
C27H30O15	Luteolin-7-O-rutinoside*	1.14	(15.26)	down
C27H30O15	Kaempferol-3-O-glucoside-7-O-rhamnoside	1.14	(5.30)	down
C27H30O15	Kaempferol-3-O-robinobioside(Biorobin)	1.14	(4.74)	down
C27H30O15	Luteolin-7-O-neohesperidoside (Lonicerin)*	1.12	(2.82)	down
C27H30O15	Apigenin-6,8-di-C-glucoside (Vicenin-2)	1.14	(5.36)	down
C27H30O15	Kaempferol-3-O-rutinoside(Nicotiflorin)*	1.14	(11.76)	down
C27H30O15	Vitexin-2"-O-glucoside	1.14	(3.98)	down
C27H30O15	"Vitexin-2""-O-galactoside"	1.14	(16.91)	down
C27H30O15	Kaempferol-3-O-neohesperidoside*	1.14	(5.04)	down
C28H32O15	Diosmetin-7-O-rutinoside (Diosmin)*	1.14	(4.71)	down
C28H32O15	Diosmetin-7-O-Neohesperidoside (Neodiosmin)*	1.14	(5.22)	down
C31H28O13	Hispidulin-7-O-(6"-O-p-Coumaroyl)Glucoside	1.14	(4.41)	down
C27H30O16	Luteolin-6,8-di-C-glucoside	1.14	(5.07)	down
C27H30O16	Quercetin-3-O-rutinoside (Rutin)	1.14	(8.15)	down
C27H30O16	Luteolin-7-O-gentiobioside	1.14	13.06	up
C27H30O16	Orientin-7-O-glucoside	1.14	(12.65)	down
C27H30O16	Quercetin-3-O-robinobioside	1.14	(16.33)	down
C30H38O14	Syringaresinol-4'-O-(6"-acetyl)glucoside	1.12	(1.23)	down
C28H32O16	Chrysoeriol-5,7-di-O-glucoside	1.14	(2.14)	down
C29H36O15	Verbascoside	1.14	(14.38)	down
C28H32O17	Isorhamnetin-3-O-sophoroside	1.14	(12.76)	down
C36H34N2O10	Abrusamide B	1.14	(8.91)	down
C36H34N2O10	Abrusamide A	1.14	(20.56)	down
C29H34O18	Limocitrin-3,7-di-O-glucoside	1.09	(1.88)	down
C33H40O21	Kaempferol-6,8-di-C-glucoside-7-O-glucoside	1.04	(1.21)	down
C42H68O13	Azukisaponin I	1.07	(1.02)	down
C34H42O21	Isorhamnetin-3-O-sophoroside-7-O-rhamnoside	1.12	(9.31)	down
C42H64O16	Ceanothic acid-3-O-glucuronic acid-glucose	1.14	(8.53)	down
C42H66O16	Bayogenin-3-O-glucuronide-28-O-glucoside	1.07	(2.92)	down
C42H62O18	Dihydroxyglycyrrhetinic acid	1.14	(8.82)	down
C45H38O18	Procyanidin C1	1.14	(13.53)	down
C45H38O18	Catechin-catechin-catechin	1.13	(4.65)	down

C45H38O18	Procyanidin C2	1.12	(6.25)	down
C48H78O17	Kaikasaponin II	1.07	(1.34)	down
C47H72O20	Medicagenic acid-3-O-glucuronide-28-O-rhamnosyl(1, 2)-arabinoside	1.14	(13.80)	down
C48H78O19	Soyasaponin βa (Soyasaponin V)	1.14	11.61	up
C48H74O20	Abrisaponin I	1.14	(11.49)	down
C48H74O20	Ceanothic acid-3-O-glucuronic acid-rutinose	1.14	(10.04)	down
C48H76O20	Hederagenin-3-O-glucuronide-28-O-glucosyl(1,2)glucoside	1.14	(7.94)	down
C48H76O20	Azukisaponin IV	1.14	(3.11)	down
C48H76O20	Bayogenin-3-O-glucuronide-28-O-(2"-O-Rhamnosyl)glucoside	1.14	(3.17)	down
C48H78O20	Abrisaponin L	1.14	(8.23)	down
C60H92O27	AcetylSoyasaponin A3	1.14	(8.99)	down
C60H92O27	Soyasaponin Ah	1.13	(7.02)	down

Supplementary Table 5. Differential metabolites of AmS and AmR sanples.

Formula	Compounds	VIP	Log2Fold_Change	Type
C6H6O	Phenol	1.16	(1.07)	down
C7H7NO	Benzamide	1.15	(1.47)	down
C8H8O2	4'-Hydroxyacetophenone	1.08	(1.21)	down
C7H7NO2	4-Aminobenzoic acid	1.11	(1.03)	down
C8H8O3	O-Anisic acid (2-Methoxybenzoic acid)	1.13	(5.78)	down
C8H8O3	Phenoxyacetic acid	1.16	(7.05)	down
C8H8O3	3-Methoxybenzoic acid	1.12	(1.51)	down
C7H6O4	2,5-Dihydroxybenzoic acid; Gentisic Acid*	1.14	(1.43)	down
C7H6O4	3,4-Dihydroxybenzoic acid (Protocatechuic acid)*	1.15	(1.40)	down
C7H6O4	2,4-Dihydroxybenzoic acid	1.16	(16.37)	down
C9H8O3	2-Hydroxycinnamic acid	1.15	(3.57)	down
C9H8O3	p-Coumaric acid	1.03	(1.76)	down
C9H8O3	Caffeic aldehyde	1.15	(1.63)	down
C10H12O2	4-Phenylbutyric Acid	1.14	(2.17)	down
C8H6O4	Phthalic acid	1.10	(1.36)	down
C8H9NO3	2-Amino-3-methoxybenzoic acid	1.14	(1.09)	down
C8H8O4	Homogentisic acid	1.15	(2.00)	down
C9H10O4	Methyl 2,4-dihydroxyphenylacetate	1.09	(1.13)	down
C9H10O4	Hydroxyphenyllactic acid	1.10	(1.28)	down
C9H10O4	3,4-Dimethoxybenzoic acid; Veratric acid	1.04	(1.42)	down
C11H12O3	p-Coumaric acid ethyl ester	1.16	(13.04)	down
C10H10O4	Isoferulic Acid*	1.15	(2.33)	down
C10H10O4	Ferulic acid*	1.14	(2.39)	down
C9H10O5	Syringic acid	1.15	(2.25)	down
C13H18O2	4-Hydroxy-3,5-diisopropylbenzaldehyde	1.14	(2.69)	down
C10H12O5	Methyl Syringate	1.09	(1.03)	down
C8H6Cl2O3	2,4-Dichlorophenoxyacetic Acid	1.07	(1.22)	down
C12H14O4	Ethyl ferulate	1.15	(4.26)	down
C11H12O5	Sinapic acid	1.11	(1.26)	down
C11H14O5	3,4'-Dihydroxy-3',5'-dimethoxypropiophenone	1.16	(2.68)	down
C13H16O6	1-Feruloyl-sn-glycerol	1.12	(1.91)	down
C13H18O7	Salicin	1.16	(8.91)	down
C12H16O8	Phloroglucinol-1-O-β-D-glucoside	1.15	(1.29)	down
C14H10O7	4-(3,4,5-Trihydroxybenzoxy)benzoic acid	1.16	(3.45)	down
C13H8O8	Brevifolin carboxylic acid	1.13	(3.44)	down
C13H18O8	3-methoxy-5-hydroxy-1-O-β-D-glucoside	1.12	(2.39)	down
C13H18O8	4-O-Glucosyl-3,4-dihydroxybenzyl	1.16	(1.22)	down

	alcohol			
C13H12O9	2-Caffeoyl-L-tartaric acid (Caftaric acid)	1.10	(1.43)	down
C14H18O8	6-O-Acetylarbutin	1.08	(1.29)	down
C14H18O8	Methyl salicylate-2-O-glucoside	1.15	(2.68)	down
C13H16O9	1-O-Galloyl-rhamnose	1.16	(10.88)	down
C13H16O9	1-O-Gentisoyl-D-glucoside*	1.14	(1.02)	down
C14H20O8	3,4-dihydroxyphenylethanol-β-D-glucoside	1.10	(1.52)	down
C14H20O8	5-(2-Hydroxyethyl)-2-O-glucosylphenol	1.16	(14.34)	down
C15H18O8	Phenylpropionic acid-O-β-D-glucoside	1.15	(3.24)	down
C15H18O8	p-Coumaric acid-4-O-glucoside	1.12	(3.82)	down
C14H18O9	Vanillic acid-4-O-glucoside	1.12	(1.54)	down
C13H16O10	Gallic acid-4-O-glucoside	1.15	(1.75)	down
C13H16O10	1-O-galloyl-β-D-glucose	1.16	(13.19)	down
C13H16O10	6-O-Galloyl-D-glucose	1.14	(14.40)	down
C15H12O9	Galloyl Methyl gallate	1.14	(15.06)	down
C16H18O8	5-O-p-Coumaroylquinic acid*	1.11	(4.35)	down
C16H18O8	3-O-p-Coumaroylquinic acid*	1.15	(2.16)	down
C15H16O9	Sinapoyl malate	1.07	(1.10)	down
C16H22O8	Coniferin	1.15	(1.12)	down
C15H20O9	Syringaldehyde-4-O-glucoside	1.10	(1.05)	down
C15H22O9	3,4,5-Trimethoxyphenyl-1-O-β-D-glucoside	1.16	(13.13)	down
C16H18O9	Chlorogenic acid (3-O-Caffeoylquinic acid)*	1.07	(1.20)	down
C16H18O9	Neochlorogenic acid (5-O-Caffeoylquinic acid)*	1.16	(13.67)	down
C16H18O9	Cryptochlorogenic acid (4-O-Caffeoylquinic acid)	1.16	(5.76)	down
C13H14O12	2-O-Galloylmucic acid	1.13	(2.18)	down
C17H20O9	1-O-Feruloylquinic acid	1.15	(2.87)	down
C17H20O9	Chlorogenic acid methyl ester	1.09	(2.67)	down
C16H20O10	Trihydroxycinnamoylquinic acid	1.16	(3.63)	down
C19H18O8	Feruloyl syringic acid	1.06	(1.25)	down
C18H26O10	Benzyl-(2"-O-xylosyl)glucoside	1.14	(1.74)	down
C20H16O11	Caffeoyl(p-Hydroxybenzoyl)tartaric acid	1.12	(1.31)	down
C20H30O12	Verbasoside	1.16	(11.88)	down
C20H20O14	2,3-Di-O-Galloyl-D-Glucose	1.13	(9.44)	down
C26H32O11	Dehydrodiconiferyl alcohol-gamma'-O-glucoside	1.16	(5.67)	down
C26H26O12	3,5-O-Dicaffeoylquinic Acid Methyl Ester	1.13	(4.43)	down
C29H36O15	Verbascoside	1.16	(14.17)	down
C31H36O18	Syringoylcaffeoylquinic acid-D-glucose	1.16	(11.12)	down

C16H16O4	(3R)-Vestitol	1.08	(1.68)	down
C15H10O6	Aureusidin	1.15	(2.24)	down
C15H10O6	Luteolin (5,7,3',4'-Tetrahydroxyflavone)	1.16	(2.43)	down
C15H10O6	Isoluteolin (Orobol)(5,7,3',4'-tetrahydroxyisoflavone)	1.15	(2.42)	down
C15H12O6	2-Hydroxy-2,3-dihydrogenistein	1.09	(1.39)	down
C15H14O6	Catechin	1.16	(3.17)	down
C15H14O6	Epicatechin	1.16	(3.17)	down
C15H14O6	5,7,3',4',5'-Pentahydroxyflavan (Tricetiflavan)	1.16	(3.47)	down
C17H14O5	Pterocarpine	1.16	(1.38)	down
C16H16O6	3'-O-Methyl-(-)-epicatechin	1.14	(3.33)	down
C15H14O7	Epigallocatechin	1.16	(3.28)	down
C21H20O6	Glisoflavone	1.16	(11.55)	down
C21H20O9	Puerarin	1.07	(2.84)	down
C20H18O10	Luteolin-8-C-arabinoside	1.13	(2.96)	down
C25H26O6	Kushenol F	1.16	(8.99)	down
C26H30O5	Kushenol U	1.16	(11.34)	down
C21H20O10	Genistein-7-O-Glucoside (Genistin)	1.16	(11.99)	down
C21H20O10	Apigenin-7-O-α-D-glucoside	1.16	(15.88)	down
C21H20O10	Sophoricoside	1.16	(15.65)	down
C21H20O10	Genistein-8-C-glucoside	1.15	(5.70)	down
C21H20O10	Apigenin-5-O-glucoside	1.16	(15.23)	down
C21H24O10	Phloretin-4'-O-glucoside (Trilobatin)	1.13	(2.20)	down
C26H30O6	Kushenol D	1.01	(1.61)	down
C26H30O6	Kushenol Ca	1.05	(1.52)	down
C26H30O6	Leachianone A	1.03	(1.11)	down
C21H20O11	Luteolin-7-O-glucoside (Cynaroside)	1.12	(1.75)	down
C21H20O11	Aureusidin-4-O-glucoside	1.16	(2.79)	down
C21H20O11	Luteolin-3'-O-glucoside	1.14	(2.65)	down
C21H20O11	Kaempferol-3-O-glucoside (Astragalin)*	1.15	(2.84)	down
C21H20O11	Kaempferol-4'-O-glucoside	1.15	(2.60)	down
C21H20O11	Kaempferol-7-O-glucoside*	1.14	(2.70)	down
C21H22O11	3',5,5',7-Tetrahydroxyflavanone-7-O-glucoside	1.16	(14.08)	down
C21H22O11	Eriodictyol-7-O-glucoside	1.15	(3.57)	down
C24H20O9	Catechin-(7,8-bc)-4β-(3,4-dihydroxyphenyl)-dihydro-2-(3H)-one	1.16	(9.64)	down
C21H24O11	Epicatechin glucoside	1.16	(13.07)	down
C27H32O6	Kushenol Da	1.16	(9.00)	down
C22H22O11	Chrysoeriol-8-C-glucoside (Scoparin)	1.16	(7.30)	down
C21H20O12	Quercetin-3-O-glucoside (Isoquercitrin)*	1.11	(3.73)	down
C23H22O11	6''-O-Acetylgenistin	1.16	(8.71)	down

C23H22O11	Apigenin-7-O-(6"-acetyl)glucoside	1.16	(9.10)	down
C22H22O12	Nepetin-7-O-glucoside	1.13	(1.00)	down
C24H22O13	Apigenin-7-O-(6"-malonyl)glucoside	1.07	(2.12)	down
C23H22O14	Tricin-7-O-saccharic acid	1.07	(1.09)	down
C24H22O14	Luteolin-7-O-(6"-malonyl)glucoside	1.10	(2.90)	down
C24H22O15	Quercetin-7-O-(6"-malonyl)glucoside	1.15	(2.72)	down
C30H26O11	Epicatechin-epiafzelechin	1.02	(1.17)	down
C26H28O14	Apigenin-6-C-glucoside-8-C-arabinoside (Schaftoside)	1.16	(4.05)	down
C26H28O14	Apigenin-6-C-arabinoside-8-C-glucoside (Isoschaftoside)	1.16	(4.25)	down
C26H28O14	"Vitexin-2""-O-xyloside"	1.16	(4.26)	down
C26H28O14	Kaempferol-3-O-arabinoside-7-O-rhamn oside	1.04	(1.07)	down
C30H26O12	Apigenin-7-O-(6"-p-Coumaryl)glucoside	1.16	(3.56)	down
C27H30O14	Apigenin-7-O-rutinoside (Isorhoifolin)	1.16	(16.81)	down
C26H28O15	Kaempferol-3-O-sambubioside	1.15	(5.91)	down
C27H32O14	Naringenin-7-O-Rutinoside(Narirutin)	1.15	(2.75)	down
C27H30O15	Luteolin-7-O-rutinoside*	1.16	(2.90)	down
C27H30O15	Kaempferol-3-O-glucoside-7-O-rhamnosi de	1.16	(3.11)	down
C27H30O15	Luteolin-7-O-neohesperidoside (Lonicerin)*	1.14	(2.79)	down
C27H30O15	Apigenin-6,8-di-C-glucoside (Vicenin-2)	1.15	(4.57)	down
C27H30O15	Vitexin-2"-O-glucoside	1.16	(4.65)	down
C27H30O15	"Vitexin-2""-O-galactoside"	1.16	(2.49)	down
C27H30O15	Kaempferol-3-O-neohesperidoside*	1.16	(3.08)	down
C27H32O15	Eriodictyol-7-O-Rutinoside (Eriocitrin)	1.15	(1.76)	down
C28H32O15	Diosmetin-7-O-rutinoside (Diosmin)*	1.16	(3.07)	down
C28H32O15	Diosmetin-7-O-Neohesperidoside (Neodiosmin)*	1.16	(3.06)	down
C31H28O13	Hispidulin-7-O-(6"-O-p-Coumaroyl)Gluc oside	1.14	(3.03)	down
C27H30O16	Luteolin-6,8-di-C-glucoside	1.16	(16.38)	down
C27H30O16	Quercetin-3-O-rutinoside (Rutin)	1.13	(3.96)	down
C27H30O16	Orientin-7-O-glucoside	1.16	(10.60)	down
C27H30O16	Orientin-2"-O-galactoside	1.16	(17.28)	down
C27H30O16	Quercetin-3-O-robinobioside	1.15	(4.76)	down
C28H32O16	Isorhamnetin-3-O-rutinoside (Narcissin)	1.01	(6.69)	down
C28H32O16	Chrysoeriol-5,7-di-O-glucoside	1.15	(3.16)	down
C36H36O17	Isovitexin-2"-O-(6"'-p-coumaroyl)glucosi de	1.12	(1.55)	down
C33H40O21	Kaempferol-6,8-di-C-glucoside-7-O-gluc oside	1.16	(12.13)	down

C34H42O21	Isorhamnetin-3-O-rutinoside-4'-O-glucoside	1.07	(2.11)	down
C45H38O18	Catechin-catechin-catechin	1.15	(3.55)	down
C9H6O4	Esculetin	1.13	(1.56)	down
C10H8O4	Scopoletin (7-Hydroxy-5-methoxycoumarin)	1.16	(16.19)	down
C10H8O4	Isoscopoletin (6-Hydroxy-7-Methoxycoumarin)	1.15	(4.09)	down
C10H8O5	Fraxetin (7,8-Dihydroxy-6-methoxycoumarin)	1.12	(2.37)	down
C10H8O6	Sideretin (5,7,8-Trihydroxy-6-methoxycoumarin)	1.10	(1.43)	down
C19H12O6	Dicumarol	1.10	(2.14)	down
C15H16O9	Esculin (6,7-DihydroxyCoumarin-6-glucoside)	1.15	(3.08)	down
C16H18O9	Scopoletin-7-O-glucoside (Scopolin)	1.15	(5.65)	down
C16H16O10	Scopoletin-7-O-glucuronide	1.12	(2.22)	down
C25H32O10	Isolariciresinol-9-O-xyloside	1.16	(11.27)	down
C26H34O11	Dihydrodehydrodiconiferyl alcohol-4-O-glucoside	1.16	(11.50)	down
C26H36O11	Secoisolariciresinol 4-O-glucoside	1.12	(1.80)	down
C26H32O12	1-Hydroxypinoresinol-1-O-Glucoside	1.16	(11.34)	down
C30H38O14	Syringaresinol-4'-O-(6''-acetyl)glucoside	1.01	(1.96)	down
C7H8N2	Benzamidine	1.15	(10.89)	down
C10H8O	2-Naphthol	1.06	(1.01)	down
C10H8O	1-Naphthol	1.06	(1.43)	down
C8H8O3	2-Hydroxy-4-Methoxybenzaldehyde	1.16	(11.28)	down
C9H8O3	4-hydroxyphenyl acrylaldehyde	1.12	(1.83)	down
C11H22O	2-Undecanone	1.16	(1.16)	down
C11H10O3	2,5-dimethyl-7-hydroxy-chromone	1.16	(10.14)	down
C10H8O4	Noreugenin; 5,7-Dihydroxy-2-Methylchromone	1.16	(2.13)	down
C12H10O5	2-methyl-5-carboxy methyl-7-Hydroxy-chromone	1.14	(3.31)	down
C16H12O7	Capillarisin	1.12	(1.02)	down
C18H17NO5	N-[3-(4-Hydroxyphenyl)acryloyl]-L-tyrosine	1.14	(3.68)	down
C21H22O5	3'-Hydroxy-4'-O-methylglabridin	1.16	(1.84)	down
C21H18O6	Hedysarimcoumestan D	1.15	(3.22)	down
C17H24O10	Majoroside	1.13	(1.57)	down
C17H24O11	Secoxyloganin	1.16	(14.37)	down
C18H24O12	Licoagroside B	1.15	(1.53)	down
C30H24O12	Procyanidin A1	1.13	(2.84)	down
C30H24O12	Procyanidin A2	1.16	(2.61)	down

C30H26O12	Procyanidin B2	1.16	(4.52)	down
C30H26O12	Procyanidin B4	1.15	(2.76)	down
C30H26O12	Procyanidin B1*	1.10	(3.73)	down
C30H26O12	Procyanidin B3*	1.16	(3.83)	down
C31H28O12	Procyanidin A6	1.16	(10.18)	down
C45H38O18	Procyanidin C1	1.15	(3.14)	down
C45H38O18	Procyanidin C2	1.10	(4.46)	down
C4H12N2	Putrescine	1.15	(1.84)	down
C5H14N2	Cadaverine	1.16	(2.93)	down
C5H11NO2	Betaine	1.15	(2.07)	down
C6H14N2O	N-Acetylputrescine	1.13	(1.53)	down
C8H9NO	N-benzylformamide	1.15	(1.19)	down
C5H9N3O2	2-Amino-4,5-dihydro-1H-imidazole-4-acetic acid	1.16	(2.46)	down
C8H17NO	Valpromide	1.16	(9.39)	down
C7H19N3	Spermidine	1.15	(1.86)	down
C9H11NO3	N-(2-Hydroxy-4-methoxyphenyl)acetamide	1.15	(1.20)	down
C11H11NO4	Methyl dioxindole-3-acetate	1.07	(1.17)	down
C13H18N2O2	p-Coumaroylputrescine	1.14	(3.56)	down
C15H20N2O2	9α-Hydroxysophoramine	1.14	(1.23)	down
C14H20N2O3	N-Feruloylputrescine	1.16	(16.65)	down
C15H24N2O3	5,9-dihydroxymatrine	1.16	(9.49)	down
C17H17NO3	P-Hydroxycinnamic acid p-hydroxyphenethylamine	1.16	(9.66)	down
C29H39N3O8	N1,N8-Bis(sinapoyl)spermidine	1.15	(6.26)	down
C36H34N2O10	Abrusamide B	1.15	(3.94)	down
C11H16O3	Isololiolide	1.15	(2.19)	down
C16H22O9	Sweroside	1.16	(2.12)	down
C16H22O10	Swertiamarin	1.16	(11.27)	down
C30H48O2	Ursolaldehyde	1.16	(11.19)	down
C30H50O3	12,13-Dihydroursolic acid	1.16	(10.58)	down
C30H48O4	2α-Hydroxyursolic acid	1.15	(1.68)	down
C30H48O4	Maslinic acid*	1.15	(1.81)	down
C30H48O4	Corosolic acid*	1.14	(1.06)	down
C30H48O4	2,3-dihydroxy-12-ursen-28-oic acid	1.15	(1.03)	down
C30H48O4	2-Hydroxyoleanolic acid	1.16	(1.77)	down
C30H50O4	Soyasapogenol A	1.02	(1.28)	down
C30H48O5	Madasiatic acid	1.15	(2.75)	down
C30H48O5	2α,3α,23-trihydroxyolean-12-en-28-oic acid	1.15	(2.63)	down
C36H58O9	Soyasapogenol B-3-O-glucuronide	1.16	(15.58)	down
C42H62O18	Dihydroxyglycyrrhetinic acid	1.14	(2.19)	down

Supplementary Table 6. Differential metabolites of AcS and AcR sanples.

Formula	Compounds	VIP	Log2Fold_Change	Type
C6H6O	Phenol	1.09	(2.16)	down
C7H7NO	Benzamide	1.08	(1.56)	down
C8H8O2	Phenyl acetate	1.07	(1.67)	down
C8H8O2	4'-Hydroxyacetophenone	1.07	(1.99)	down
C7H7NO2	4-Aminobenzoic acid	1.05	(1.34)	down
C7H6O3	2,4-Dihydroxybenzaldehyde	1.09	(1.93)	down
C8H8O3	O-Anisic acid (2-Methoxybenzoic acid)	1.08	(3.50)	down
C8H8O3	Phenoxyacetic acid	1.03	(3.74)	down
C8H8O3	2,5-Dihydroxyacetophenone	1.07	(2.31)	down
C8H8O3	3-Methoxybenzoic acid	1.09	(3.56)	down
C7H7NO3	3-Hydroxyanthranilic acid	1.07	(2.38)	down
C7H6O4	2,5-Dihydroxybenzoic acid; Gentisic Acid*	1.06	(2.21)	down
C7H6O4	3,4-Dihydroxybenzoic acid (Protocatechuic acid)*	1.06	(2.30)	down
C7H6O4	2,4-Dihydroxybenzoic acid	1.09	(3.13)	down
C8H10O3	Vanillyl alcohol	1.09	(9.20)	down
C9H8O3	2-Hydroxycinnamic acid	1.01	(3.40)	down
C9H8O3	p-Coumaric acid	1.09	(2.78)	down
C9H8O3	Caffeic aldehyde	1.09	(2.93)	down
C8H6O4	Phthalic acid	1.07	(1.31)	down
C8H6O4	Terephthalic acid	1.06	(2.61)	down
C9H10O3	3-(4-Hydroxyphenyl)-propionic acid	1.04	(2.98)	down
C8H9NO3	2-Amino-3-methoxybenzoic acid	1.01	(1.17)	down
C8H8O4	3,4-Dihydroxybenzeneacetic acid	1.08	(1.75)	down
C8H8O4	2',4',6'-Trihydroxyacetophenone	1.08	(2.33)	down
C8H8O4	Protocatechuic Acid Methyl Ester	1.05	(2.12)	down
C7H6O5	2,4,6-Trihydroxybenzoic acid	1.09	(2.86)	down
C9H8O4	Caffeic acid	1.09	(2.07)	down
C9H10O4	Hydroxyphenyllactic acid	1.04	(1.28)	down
C9H10O4	Vanillic acid methyl ester	1.09	(1.27)	down
C6H4N2O5	2,4-Dinitrophenol	1.06	(1.36)	down
C8H8O5	Methyl gallate	1.07	(6.13)	down
C8H8O5	3-O-Methylgallic acid	1.08	(3.92)	down
C9H12O4	Antiarol; 3,4,5-Trimethoxyphenol	1.03	(1.26)	down
C10H10O4	Isoferulic Acid*	1.09	(2.74)	down
C10H10O4	Ferulic acid*	1.10	(2.77)	down
C10H10O4	Vanillin acetate	1.09	(1.80)	down
C9H10O5	Syringic acid	1.09	(3.47)	down
C11H12O5	Sinapic acid	1.09	(3.57)	down

$C_{11}H_{14}O_5$	3,4'-Dihydroxy-3',5'-dimethoxypropiophenone	1.07	(1.75)	down
$C_{12}H_{14}O_5$	Methyl sinapate	1.07	(2.71)	down
$C_{12}H_{16}O_6$	Phenyl-beta-D-glucoside	1.03	(2.84)	down
$C_{13}H_{18}O_7$	Salicin	1.09	(2.45)	down
$C_{12}H_{16}O_8$	Phloroglucinol-1-O-β-D-glucoside	1.09	(4.46)	down
$C_{13}H_{18}O_8$	3-methoxy-5-hydroxy-1-O-β-D-glucoside	1.09	(5.04)	down
$C_{13}H_{18}O_8$	4-O-Glucosyl-3,4-dihydroxybenzyl alcohol	1.09	(3.30)	down
$C_{13}H_{12}O_9$	2-Caffeoyl-L-tartaric acid (Caftaric acid)	1.05	(1.14)	down
$C_{14}H_{16}O_8$	1-O-Caffeoyl xylose	1.10	(10.77)	down
$C_{14}H_{18}O_8$	6-O-Acetylarbutin	1.08	(1.44)	down
$C_{14}H_{18}O_8$	Methyl salicylate-2-O-glucoside	1.09	(1.25)	down
$C_{13}H_{16}O_9$	1-O-Galloyl-rhamnose	1.10	(12.82)	down
$C_{13}H_{16}O_9$	1-O-Gentisoyl-D-glucoside*	1.05	(1.01)	down
$C_{14}H_{20}O_8$	3,4-dihydroxyphenylethanol-β-D-glucoside	1.10	(9.94)	down
$C_{14}H_{20}O_8$	5-(2-Hydroxyethyl)-2-O-glucosylphenol	1.09	(4.22)	down
$C_{14}H_{10}O_9$	Digallic acid	1.09	(14.99)	down
$C_{15}H_{18}O_8$	1-O-p-Coumaroyl-β-D-glucose	1.07	(3.12)	down
$C_{15}H_{18}O_8$	Phenylpropionic acid-O-β-D-glucoside	1.09	(6.31)	down
$C_{15}H_{18}O_8$	p-Coumaric acid-4-O-glucoside	1.09	(6.70)	down
$C_{15}H_{20}O_8$	Demethyl coniferin	1.02	(1.74)	down
$C_{14}H_{18}O_9$	Vanillic acid-4-O-glucoside	1.09	(5.24)	down
$C_{13}H_{16}O_{10}$	Gallic acid-4-O-glucoside	1.05	(1.09)	down
$C_{13}H_{16}O_{10}$	1-O-galloyl-β-D-glucose	1.10	(14.64)	down
$C_{13}H_{16}O_{10}$	6-O-Galloyl-D-glucose	1.10	(17.19)	down
$C_{15}H_{12}O_9$	Galloyl Methyl gallate	1.06	(7.89)	down
$C_{16}H_{18}O_8$	5-O-p-Coumaroylquinic acid*	1.09	(4.51)	down
$C_{15}H_{16}O_9$	Sinapoyl malate	1.07	(1.89)	down
$C_{15}H_{18}O_9$	Vanillic Acid-4-O-Glucuronide	1.05	(2.14)	down
$C_{15}H_{18}O_9$	1-O-Caffeoyl-β-D-glucose	1.10	(11.75)	down
$C_{15}H_{18}O_9$	6-O-Caffeoyl-D-glucose	1.09	(2.42)	down
$C_{16}H_{22}O_8$	Coniferin	1.10	(2.99)	down
$C_{14}H_{18}O_{10}$	1-O-(3,4-Dihydroxy-5-methoxy-benzoyl)-glucoside	1.10	(16.67)	down
$C_{15}H_{22}O_9$	3,4,5-Trimethoxyphenyl-1-O-β-D-glucoside	1.10	(13.58)	down
$C_{16}H_{18}O_9$	Neochlorogenic acid (5-O-Caffeoylquinic acid)*	1.10	(14.80)	down
$C_{16}H_{18}O_9$	Cryptochlorogenic acid (4-O-Caffeoylquinic acid)	1.09	(5.18)	down

C16H20O9	4-O-β-D-glucosylferulic acid	1.09	(3.41)	down
C16H20O9	1-O-Feruloyl-β-D-glucose	1.09	(3.64)	down
C13H14O12	2-O-Galloylmucic acid	1.09	(1.49)	down
C16H20O10	Trihydroxycinnamoylquinic acid	1.09	(1.86)	down
C17H22O10	1-O-Eudesmoylquinic acid	1.10	(11.96)	down
C17H22O10	4-O-Glucosyl-sinapate	1.10	(11.47)	down
C17H22O10	1-O-Sinapoyl-D-glucose	1.10	(13.96)	down
C21H14O13	Trigallic acid	1.08	(7.46)	down
C20H20O14	2,3-Di-O-Galloyl-D-Glucose	1.09	(12.78)	down
C26H32O11	Dehydrodiconiferyl alcohol-gamma'-O-glucoside	1.10	(12.34)	down
C26H26O12	3,5-O-Dicaffeoylquinic Acid Methyl Ester	1.10	(4.92)	down
C31H36O18	Syringoylcaffeoylquinic acid-D-glucose	1.10	(12.58)	down
C15H14O3	(±)-Equol; 7,4'-Homoisoflavane	1.09	(9.06)	down
C16H12O5	Genkwanin (Apigenin 7-methyl ether)	1.08	(1.56)	down
C15H10O6	Aureusidin	1.08	(1.30)	down
C15H10O6	Isoluteolin (Orobol)(5,7,3',4'-tetrahydroxyisoflavone)	1.08	(1.16)	down
C16H16O6	3'-O-Methyl-(-)-epicatechin	1.07	(2.09)	down
C20H16O5	Glabrone	1.06	(2.17)	down
C20H18O5	Licoflavone C	1.07	(1.80)	down
C21H20O6	Glisoflavone	1.10	(10.63)	down
C25H26O4	Isolicoflavone B	1.07	(2.34)	down
C25H28O4	Glabrol	1.08	(2.17)	down
C20H18O10	Luteolin-8-C-arabinoside	1.10	(11.98)	down
C25H26O6	Kushenol F	1.10	(9.13)	down
C26H30O5	Kushenol U	1.10	(11.56)	down
C21H20O10	Apigenin-7-O-α-D-glucoside	1.10	(14.57)	down
C21H20O10	Sophoricoside	1.10	(14.42)	down
C21H20O10	Genistein-8-C-glucoside	1.09	(4.83)	down
C21H20O10	Apigenin-5-O-glucoside	1.08	(3.25)	down
C21H24O10	Phloretin-4'-O-glucoside (Trilobatin)	1.04	(1.93)	down
C26H30O6	Kushenol Ca	1.10	(8.36)	down
C21H20O11	Luteolin-7-O-glucoside (Cynaroside)	1.06	(1.70)	down
C21H20O11	Aureusidin-4-O-glucoside	1.10	(18.32)	down
C21H20O11	Luteolin-3'-O-glucoside	1.10	(18.34)	down
C21H20O11	Amoenin	1.09	(5.63)	down
C21H20O11	Kaempferol-3-O-glucoside (Astragalin)*	1.10	(18.37)	down
C21H20O11	Kaempferol-4'-O-glucoside	1.10	(18.24)	down
C21H20O11	Kaempferol-7-O-glucoside*	1.09	(3.56)	down
C21H22O11	3',5,5',7-Tetrahydroxyflavanone-7-O-gl	1.10	(12.90)	down

	ucoside			
C21H22O11	Eriodictyol-7-O-glucoside	1.10	(12.25)	down
C21H24O11	Epicatechin glucoside	1.10	(10.81)	down
C22H22O11	Chrysoeriol-8-C-glucoside (Scoparin)	1.09	(5.43)	down
C22H22O11	Hispidulin-7-O-Glucoside*	1.09	(2.81)	down
C21H20O12	Isoquercitrin	1.08	(1.99)	down
C21H20O12	Quercetin-7-O-glucoside*	1.10	(13.25)	down
C22H22O12	Isorhamnetin-7-O-glucoside (Brassicin)	1.08	(3.58)	down
C22H22O12	Nepetin-7-O-glucoside	1.09	(3.90)	down
C23H20O13	Kaempferol-3-O-(2"-O-acetyl)glucuronide	1.09	(4.75)	down
C24H22O13	Apigenin-7-O-(6"-malonyl)glucoside	1.09	(9.85)	down
C24H22O14	Kaempferol-3-O-(6"-malonyl)glucoside*	1.10	(16.59)	down
C24H22O14	Luteolin-7-O-(6"-malonyl)glucoside	1.03	(2.56)	down
C25H24O14	Diosmetin-7-O-(6"-malonyl)glucoside*	1.08	(2.86)	down
C25H24O14	Chrysoeriol-7-O-(6"-malonyl)glucoside*	1.09	(1.78)	down
C24H22O15	Quercetin-7-O-(6"-malonyl)glucoside	1.10	(14.25)	down
C26H28O14	Apigenin-6-C-glucoside-8-C-arabinoside (Schaftoside)	1.09	(4.74)	down
C26H28O14	Apigenin-6-C-arabinoside-8-C-glucoside (Isoschaftoside)	1.09	(3.89)	down
C26H28O14	"Vitexin-2""-O-xyloside"	1.09	(4.29)	down
C26H28O14	Kaempferol-3-O-arabinoside-7-O-rhamnoside	1.05	(3.45)	down
C26H28O14	Isovitexin-2"xyloside	1.08	(2.82)	down
C26H28O14	Apigenin-7-O-(2"-glucosyl)arabinoside	1.09	(9.76)	down
C30H26O12	Apigenin-7-O-(6"-p-Coumaryl)glucoside	1.10	(13.63)	down
C27H30O14	Apigenin-7-O-rutinoside (Isorhoifolin)	1.10	(13.71)	down
C27H30O14	Vitexin-2"-O-rhamnoside	1.05	(1.61)	down
C26H28O15	Kaempferol-3-O-sambubioside	1.09	(4.18)	down
C27H30O15	Luteolin-7-O-rutinoside*	1.09	(9.86)	down
C27H30O15	Kaempferol-3-O-robinobioside(Biorobin)	1.10	(11.00)	down
C27H30O15	Vitexin-2"-O-glucoside	1.10	(4.06)	down
C28H34O14	Didymin (Isosakuranetin-7-O-rutinoside)	1.10	(10.47)	down
C28H32O15	Diosmetin-7-O-rutinoside (Diosmin)*	1.08	(5.28)	down
C28H32O15	Diosmetin-7-O-Neohesperidoside (Neodiosmin)*	1.10	(11.81)	down
C31H28O13	Hispidulin-7-O-(6"-O-p-Coumaroyl)Glucoside	1.09	(10.85)	down

C27H30O16	Luteolin-6,8-di-C-glucoside	1.10	(12.28)	down
C27H30O16	Luteolin-7,3'-di-O-glucoside	1.02	(1.85)	down
C27H30O16	Luteolin-7-O-gentiobioside	1.08	(2.71)	down
C27H30O16	Orientin-2''-O-galactoside	1.09	(4.61)	down
C27H30O16	Quercetin-3-O-robinobioside	1.10	(9.34)	down
C28H32O16	Isorhamnetin-3-O-rutinoside (Narcissin)	1.10	(12.64)	down
C28H32O16	Chrysoeriol-5,7-di-O-glucoside	1.02	(3.25)	down
C27H30O17	Quercetin-3-O-sophoroside (Baimaside)	1.04	(1.65)	down
C33H40O21	Kaempferol-6,8-di-C-glucoside-7-O-glucoside	1.10	(12.55)	down
C34H42O21	Isorhamnetin-3-O-rutinoside-4'-O-glucoside	1.09	(7.77)	down
C16H12O5	1,3-dihydroxy-6-methoxy-7-methylanthraquinone	1.06	(1.54)	down
C9H6O2	Coumarin	1.04	(1.27)	down
C9H6O4	Esculetin	1.10	(13.46)	down
C10H8O4	Scopoletin (7-Hydroxy-5-methoxycoumarin)	1.10	(14.33)	down
C10H8O4	Isoscopoletin (6-Hydroxy-7-Methoxycoumarin)	1.05	(4.62)	down
C10H8O5	Fraxetin (7,8-Dihydroxy-6-methoxycoumarin)	1.09	(1.90)	down
C10H8O6	Sideretin (5,7,8-Trihydroxy-6-methoxycoumarin)	1.07	(3.55)	down
C19H12O6	Dicumarol	1.08	(2.63)	down
C15H16O9	Esculin (6,7-DihydroxyCoumarin-6-glucoside)	1.09	(4.81)	down
C16H18O9	Scopoletin-7-O-glucoside (Scopolin)	1.09	(6.40)	down
C16H16O10	Scopoletin-7-O-glucuronide	1.10	(10.28)	down
C17H20O10	Isofraxidin-7-O-glucoside	1.10	(11.29)	down
C21H26O13	Scopoletin-7-O-xylosyl(1→6)glucoside	1.10	(15.02)	down
C25H32O10	Isolariciresinol-9-O-xyloside	1.09	(10.90)	down
C26H34O11	Isolariciresinol-9'-O-glucoside	1.07	(1.24)	down
C26H34O11	Dihydrodehydrodiconiferyl alcohol-4-O-glucoside	1.10	(12.28)	down
C26H36O11	Secoisolariciresinol 4-O-glucoside	1.09	(1.39)	down
C26H32O12	1-Hydroxypinoresinol-4'-O-Glucoside	1.10	(8.74)	down
C30H38O14	Syringaresinol-4'-O-(6''-acetyl)glucoside	1.08	(1.79)	down
C8H8O3	2-Hydroxy-4-Methoxybenzaldehyde	1.10	(11.18)	down
C10H10O2	5-Hydroxy-1-tetralone	1.09	(3.64)	down
C9H8O3	4-hydroxyphenyl acrylaldehyde	1.09	(2.93)	down
C11H10O3	2,5-dimethyl-7-hydroxy-chromone	1.10	(12.65)	down
C10H8O4	Noreugenin;	1.09	(5.85)	down

C12H10O5	5,7-Dihydroxy-2-Methylchromone 2-methyl-5-carboxy methyl-7-Hydroxy-chromone	1.08	(5.84)	down
C17H12O6	Hedysarimcoumestan A	1.07	(1.17)	down
C16H12O7	Capillarisin	1.09	(3.05)	down
C21H22O5	3'-Hydroxy-4'-O-methylglabridin	1.08	(1.66)	down
C21H18O6	Hedysarimcoumestan D	1.09	(2.56)	down
C19H30O8	Roseoside	1.09	(4.15)	down
C17H24O10	Majoroside	1.07	(1.33)	down
C7H6O5	Gallic acid	1.08	(6.98)	down
C15H8O8	3-O-Methylellagic acid	1.04	(2.40)	down
C45H38O18	Procyanidin C1	1.00	(2.14)	down
C5H11N	Piperidine	1.09	(1.16)	down
C5H14N2	Cadaverine	1.10	(3.36)	down
C6H13NO2	6-Deoxyfagomine	1.09	(1.31)	down
C7H7NO2	Nicotinic Acid Methyl Ester(Methyl Nicotinate)	1.07	(1.34)	down
C5H9N3O2	2-Amino-4,5-dihydro-1H-imidazole-4-a cetic acid	1.09	(3.34)	down
C10H12N2	Tryptamine	1.06	(1.12)	down
C6H11NO4	DL-2-Aminoadipic acid	1.09	(1.26)	down
C7H8N4O2	Theobromine	1.01	(1.01)	down
C11H9O2N	3-amino-2-naphthoic acid	1.09	(1.81)	down
C11H14N2O	Cytisine	1.06	(1.57)	down
C11H12N2O2	1-Methoxy-indole-3-acetamide	1.09	(1.84)	down
C11H11NO3	Methoxyindoleacetic acid	1.07	(1.81)	down
C13H18N2O2	p-Coumaroylputrescine	1.08	(3.62)	down
C15H20N2O	Sophoramine	1.08	(2.49)	down
C15H22N2O	7,11-Dehydromatrine	1.07	(2.34)	down
C15H22N2O	Sophocarpine	1.06	(2.54)	down
C15H22N2O	5,6-dehydrolupanine	1.07	(2.89)	down
C15H22N2O	Isosophocarpine	1.07	(2.39)	down
C15H24N2O	Isomatrine	1.08	(2.28)	down
C15H24N2O	Matrine	1.07	(2.34)	down
C15H20N2O2	9α-Hydroxysophoramine	1.10	(1.36)	down
C15H22N2O2	9α-Hydroxysophocarpine	1.07	(2.48)	down
C15H22N2O2	5α-Hydroxysophocarpine	1.08	(2.28)	down
C15H22N2O2	Oxysophocarpine	1.08	(2.57)	down
C14H20N2O3	N-Feruloylputrescine	1.10	(17.43)	down
C15H24N2O2	Sophoridine N-oxide	1.08	(2.12)	down
C15H24N2O2	9α-Hydroxymatrine	1.06	(2.06)	down
C15H24N2O2	Oxymatrine	1.07	(2.11)	down
C15H24N2O3	5,9-dihydroxymatrine	1.10	(9.81)	down
C15H24N2O3	(+)-5α,9α-Dihydroxymatrine	1.05	(1.95)	down

C14H20N4O3	Caffeoylagmatine	1.10	(16.45)	down
C20H39NO2	N-Oleoylethanolamine	1.09	(2.19)	down
C15H21NO7	N-benzoyl-2-aminoethyl-β-D-glucoside	1.09	(1.37)	down
C20H23N7O7	10-Formyltetrahydrofuran	1.08	(1.09)	down
C29H39N3O8	N1,N8-Bis(sinapoyl)spermidine	1.10	(11.53)	down
C11H16O3	Isololiolide	1.07	(3.57)	down
C16H22O9	Sweroside	1.09	(2.05)	down
C16H22O10	Swertiamarin	1.10	(11.40)	down
C30H48O2	Ursolaldehyde	1.10	(12.38)	down
C30H50O3	12,13-Dihydroursolic acid	1.10	(10.30)	down
C30H50O4	Soyasapogenol A	1.07	(1.55)	down
C42H62O18	Dihydroxyglycyrrhetinic acid	1.09	(8.96)	down
C8H8O2	2-Methylbenzoic acid	1.07	1.05	up
C9H8O2	p-Coumaraldehyde	1.06	1.45	up
C8H6O3	Benzoylformic acid	1.08	1.02	up
C9H10O2	p-Coumaryl alcohol	1.07	1.17	up
C8H10O3	3,4-Dimethoxyphenol	1.07	1.15	up
C9H10O3	Methyl anisate	1.09	8.15	up
C8H8O4	Homogentisic acid	1.10	14.26	up
C8H8O4	4-Methoxysalicylic Acid	1.09	2.02	up
C11H12O4	Sinapinaldehyde	1.08	2.71	up
C14H12O3	Benzyl salicylate	1.07	1.38	up
C17H14O5	Puerol A	1.07	2.96	up
C13H16O8	Salicylic acid-2-O-glucoside	1.10	1.65	up
C16H16O7	Trans-5-O-(p-Coumaroyl)shikimate	1.09	3.11	up
C14H18O9	1-O-Vanilloyl-D-Glucose	1.10	12.02	up
C16H18O8	3-O-p-Coumaroylquinic acid*	1.02	1.01	up
C18H26O10	Benzyl-(2''-O-xylosyl)glucoside	1.08	1.65	up
C22H22O8	1-O-Feruloyl-3-O-p-Coumaroylglycerol	1.08	1.46	up
C19H28O10	Hydrangeifolin I	1.10	11.17	up
C15H10O4	Daidzein	1.09	2.69	up
C15H10O4	7,4'-Dihydroxyflavone	1.09	4.13	up
C15H10O4	6,7-Dihydroxyflavone	1.06	3.37	up
C15H12O4	Isoliquiritigenin	1.10	3.20	up
C15H12O4	Liquiritigenin	1.10	4.26	up
C15H12O4	3,9-Dihydroxypterocarpan	1.10	3.04	up
C15H12O4	2,4,4'-trihydroxychalcone	1.10	3.21	up
C16H12O4	Formononetin (7-Hydroxy-4'-methoxyisoflavone)	1.09	3.07	up
C15H10O5	Genistein	1.09	3.14	up
C15H10O5	2'-Hydroxydaidzein	1.09	1.99	up
C15H10O5	3',4',7-Trihydroxyflavone	1.08	3.61	up
C15H10O5	Apigenin	1.06	1.85	up
C16H14O4	Echinatin	1.09	3.00	up

C15H12O5	Pinobanksin*	1.10	3.83	up
C15H12O5	Naringenin (5,7,4'-Trihydroxyflavanone)*	1.10	3.85	up
C15H12O5	Butin	1.10	3.87	up
C15H14O5	Epiafzelechin	1.08	1.96	up
C16H10O5	pseudobaptigenin	1.10	3.04	up
C17H14O4	7,4'-Di-O-methyldaidzein	1.10	4.89	up
C16H12O5	Biochanin A*	1.09	3.31	up
C16H12O5	Prunetin (5,4'-Dihydroxy-7-methoxyisoflavone)*	1.10	3.69	up
C16H12O5	3'-Methoxydaidzein	1.09	3.73	up
C16H12O5	Calycosin	1.09	1.29	up
C16H12O5	Glycitein	1.10	1.70	up
C16H12O5	Acacetin	1.09	3.44	up
C15H12O6	2,6,7,4'-Tetrahydroxyisoflavanone	1.07	1.10	up
C17H12O5	7-O-methylpseudobaptigenin	1.10	5.21	up
C16H12O6	Aracarpene 2	1.09	2.91	up
C16H12O6	Aracarpene 1	1.09	2.85	up
C16H12O6	Pratensein	1.10	2.51	up
C17H16O6	Cajanol	1.08	2.75	up
C17H14O7	5,7-Dihydroxy-2'-methoxy-3',4'-methyl eneoxydihydroisoflavone	1.10	9.89	up
C20H18O5	Psoralenol	1.10	14.03	up
C21H18O10	Chrysin-7-O-Glucuronide	1.09	10.64	up
C22H22O9	Formononetin-7-O-glycoside (Ononin)	1.08	2.50	up
C21H22O10	Naringenin-7-O-glucoside (Prunin)	1.04	2.59	up
C22H22O10	Glycitin	1.09	2.18	up
C22H22O10	Calycosin-7-O-glucoside	1.09	2.16	up
C22H22O10	3'-Methoxydaidzin	1.05	1.73	up
C22H22O10	Prunetin-5-O-glucoside	1.05	1.53	up
C22H22O10	Trifolirhizin (Maackiain-3-O-glucoside)	1.10	9.87	up
C21H20O11	Kaempferol-3-O-galactoside (Trifolin)	1.07	2.19	up
C22H22O11	Diosmetin-7-O-galactoside*	1.10	12.23	up
C22H22O11	Diosmetin-7-O-glucoside*	1.10	12.51	up
C24H24O11	6''-O-Acetylglycitin	1.07	1.78	up
C23H22O12	Kaempferol-3-O-(6''-acetyl)glucoside	1.06	1.26	up
C23H24O12	Tricin-7-O-Glucoside	1.05	1.39	up
C24H22O12	6''-O-Malonyldaidzin	1.08	2.52	up
C24H24O12	Pinocembrin-7-O-(6''-O-malonyl)glucos ide	1.09	3.29	up
C25H24O12	Formononetin-7-O-(6''-Malonyl)glucosi de	1.09	2.48	up
C24H22O13	6''-O-Malonylgenistin	1.02	1.05	up
C24H24O13	Naringenin-7-O-(6''-malonyl)glucoside	1.08	3.40	up

$C_{25}H_{24}O_{13}$	6''-O-Malonylglycitin	1.09	1.72	up
$C_{26}H_{28}O_{13}$	Daidzein-7-O-apiosyl(1→6)glucoside	1.09	3.22	up
$C_{26}H_{28}O_{13}$	Daidzein-7-O-Glucoside-4'-O-Apioside	1.09	3.43	up
$C_{27}H_{30}O_{13}$	Kushenol O	1.09	10.82	up
$C_{27}H_{30}O_{13}$	Glycyroside	1.09	2.76	up
$C_{26}H_{28}O_{14}$	Apigenin-6-C-(2''-xylosyl)glucoside	1.09	11.93	up
$C_{27}H_{30}O_{15}$	Luteolin-7-O-neohesperidoside (Lonicerin)*	1.09	10.07	up
$C_{29}H_{34}O_{18}$	Limocitrin-3,7-di-O-glucoside	1.09	4.55	up
$C_{33}H_{40}O_{20}$	Kaempferol-3-O-sophoroside-7-O-rhamnoside	1.09	8.23	up
$C_{16}H_{12}O_5$	5-Hydroxy-3,7dimethoxy-1,4-phenanthrenequinone	1.10	12.81	up
$C_{16}H_{12}O_5$	Emodin-1-methyl ether	1.10	2.46	up
$C_{23}H_{20}O_{12}$	Rhein-8-O-(6'-O-acetyl)glucoside	1.08	2.82	up
$C_9H_8O_2$	3,4-Dihydrocoumarin	1.07	1.61	up
$C_9H_6O_3$	4-Hydroxycoumarin	1.09	2.87	up
$C_9H_6O_3$	Umbelliferone	1.09	3.67	up
$C_{10}H_8O_3$	6-Hydroxy-4-methylcoumarin	1.08	2.31	up
$C_9H_6O_4$	Daphnetin	1.05	1.60	up
$C_{20}H_{16}O_5$	Psoralidin	1.10	10.55	up
$C_{21}H_{20}O_5$	1-Methoxyphaseollin	1.02	1.02	up
$C_9H_6O_3$	7-Hydroxy-4-chromone	1.08	2.66	up
$C_7H_{14}N_2O_3$	N-α-Acetyl-L-ornithine	1.09	1.19	up
$C_{11}H_{22}N_2O_4S$	Pantetheine	1.07	3.51	up
$C_{16}H_{14}O_5$	kushenin	1.09	2.62	up
$C_{17}H_{18}O_4$	6,7-cis-Dihydroxy-2-(2-phenylethyl)-5,6,7,8-tetrahydrochromone	1.09	1.87	up
$C_{18}H_{17}NO_5$	N-[3-(4-Hydroxyphenyl)acryloyl]-L-tyrosine	1.09	9.66	up
$C_{17}H_{12}O_8$	3,3',4-O-Trimethylellagic acid	1.09	9.49	up
$C_4H_{12}N_2$	Putrescine	1.09	1.12	up
C_8H_7N	Indole	1.09	2.07	up
C_9H_7N	Isoquinoline	1.07	2.12	up
$C_6H_{14}N_2O$	N-Acetylputrescine	1.09	2.51	up
C_8H_9NO	2'-Aminoacetophenone	1.10	10.44	up
C_9H_7NO	Indole-3-carboxaldehyde	1.10	3.41	up
$C_9H_7NO_2$	Indole-3-carboxylic acid	1.10	2.76	up
$C_5H_{15}NO_4P+$	O-Phosphocholine	1.09	3.37	up
$C_{10}H_9NO_3$	2-Oxo-3,4-dihydro-1H-quinoline-3-carboxylic acid	1.08	1.72	up
$C_{12}H_{14}N_2O_2$	Abrine	1.09	1.73	up
$C_{14}H_{18}N_2O_2$	Hypaphorine	1.08	1.70	up
$C_{14}H_{20}NO_3^+$	4-Coumaroylcholine	1.08	1.55	up

$C_{30}H_{46}O_3$	Betulonic acid	1.00	2.07	up
$C_{30}H_{46}O_3$	Urs-12(13)-en-3-one-28-oic acid	1.05	2.15	up
$C_{30}H_{48}O_3$	Ursolic acid	1.08	1.28	up
$C_{30}H_{48}O_3$	3-Epiursolic acid	1.09	1.24	up
$C_{30}H_{48}O_5$	Madasiatic acid	1.06	1.44	up
$C_{30}H_{48}O_5$	2α,3α,23-trihydroxyolean-12-en-28-oic acid	1.07	1.26	up
$C_{30}H_{48}O_6$	2α,3α,19α,23-tetrahydroxy-12-ursen-28-oic acid	1.10	11.22	up
$C_{36}H_{58}O_9$	Soyasapogenol B-3-O-glucuronide	1.09	3.38	up
$C_{41}H_{66}O_{13}$	Soyasaponin IV	1.09	2.48	up
$C_{42}H_{68}O_{13}$	Azukisaponin I	1.09	4.25	up
$C_{42}H_{68}O_{14}$	Soyasaponin βb'	1.09	4.45	up
$C_{42}H_{68}O_{14}$	Soyasaponin III	1.09	4.40	up
$C_{42}H_{64}O_{16}$	Ceanothic acid-3-O-glucuronic acid-glucose	1.08	3.56	up
$C_{42}H_{66}O_{16}$	Bayogenin-3-O-glucuronide-28-O-glucoside	1.09	3.98	up
$C_{48}H_{74}O_{17}$	Soyasaponin γg	1.09	4.54	up
$C_{48}H_{78}O_{17}$	Kaikasaponin II	1.09	3.87	up
$C_{48}H_{76}O_{18}$	Soyasapogenol E-3-O-rhamnosyl(1,2)glucosyl(1,2)glucuronide	1.09	4.09	up
$C_{48}H_{76}O_{18}$	Soyasaponin βe	1.09	4.16	up
$C_{48}H_{78}O_{18}$	Abrisaponin A	1.09	3.61	up
$C_{48}H_{78}O_{18}$	Soyasaponin βb (Soyasaponin I)	1.09	3.55	up
$C_{47}H_{72}O_{20}$	Medicagenic acid-3-O-glucuronide-28-O-rhamnosyl(1,2)-arabinoside	1.09	4.29	up
$C_{48}H_{78}O_{19}$	Soyasaponin βa (Soyasaponin V)	1.09	4.27	up
$C_{48}H_{74}O_{20}$	Abrisaponin I	1.09	5.80	up
$C_{48}H_{74}O_{20}$	Ceanothic acid-3-O-glucuronic acid-rutinose	1.04	3.28	up
$C_{48}H_{76}O_{20}$	Azukisaponin IV	1.09	4.41	up
$C_{48}H_{76}O_{20}$	Bayogenin-3-O-glucuronide-28-O-(2''-O-Rhamnosyl)glucoside	1.09	4.56	up
$C_{48}H_{78}O_{20}$	Abrisaponin L	1.10	14.54	up
$C_{54}H_{84}O_{21}$	Soyasaponin βg (Soyasaponin VI)	1.04	3.43	up
$C_{54}H_{88}O_{23}$	Abrisaponin SB	1.09	5.22	up
$C_{60}H_{92}O_{27}$	AcetylSoyasaponin A3	1.10	10.64	up
$C_{60}H_{92}O_{27}$	Soyasaponin Ah	1.09	9.87	up

Supplementary Table 7. 20 chemical makers with VIP values larger than 1.5

Formulate	Compounds	Classification	VIP
$C_{26}H_{28}O_{14}$	Isovitexin-2"xyloside	Flavonoid carbonoside	2.53
$C_{27}H_{30}O_{15}$	"Vitexin-2""-O-galactoside"	Flavonoid carbonoside	2.30
$C_{24}H_{22}O_{15}$	Quercetin-7-O-(6"-malonyl)glucoside	Flavonols	2.23
$C_{9}H_{10}O_{3}$	Ethylsalicylate	Phenolic acids	2.17
$C_{8}H_{15}NO_{3}$	6-Acetamidohexanoic acid	Alkaloids	2.16
$C_{21}H_{18}O_{11}$	Rhein-8-O-glucoside	Anthraquinone	2.09
$C_{48}H_{78}O_{19}$	Soyasaponin V	Triterpene Saponin	2.04
$C_{48}H_{76}O_{20}$	Hederagenin-3-O-glucuronide-28-O-glucosyl(1,2)glucoside	Triterpene Saponin	2.02
$C_{11}H_{11}NO_{4}$	Methyl dioxindole-3-acetate	Alkaloids	2.00
$C_{27}H_{30}O_{15}$	Luteolin-7-O-rutinoside*	Flavonoid	1.92
$C_{9}H_{10}O_{4}$	Veratric acid	Phenolic acids	1.90
$C_{27}H_{30}O_{16}$	Quercetin-3-O-robinobioside	Flavonols	1.84
$C_{21}H_{20}O_{9}$	Daidzin	Isoflavones	1.83
$C_{34}H_{42}O_{21}$	Isorhamnetin-3-O-sophoroside-7-O-rhamnoside	Flavonols	1.82
$C_{28}H_{32}O_{17}$	Isorhamnetin-3-O-sophoroside	Flavonols	1.76
$C_{16}H_{16}O_{10}$	Scopoletin-7-O-glucuronide	Coumarins	1.74
$C_{42}H_{62}O_{18}$	Dihydroxyglycyrrhetinic acid	Triterpene	1.57
$C_{47}H_{72}O_{20}$	Medicagenic acid-3-O-glucuronide-28-O-rhamnosyl(1,2)-arabinoside	Triterpene Saponin	1.54
$C_{48}H_{74}O_{20}$	Abrisaponin I	Triterpene Saponin	1.53
$C_{15}H_{14}O_{5}$	Epiafzelechin	Flavanols	1.52
